# Targeting the Phosphatidylserine-Immune Checkpoint with a Small-Molecule Maytansinoid Conjugate

**DOI:** 10.1021/acs.jmedchem.2c00631

**Published:** 2022-09-26

**Authors:** Chen-Fu Lo, Tai-Yu Chiu, Yu-Tzu Liu, Pei-Yun Pan, Kuan-Liang Liu, Chia-Yu Hsu, Ming-Yu Fang, Yu-Chen Huang, Teng-Kuang Yeh, Tsu-An Hsu, Chiung-Tong Chen, Li-Rung Huang, Lun Kelvin Tsou

**Affiliations:** † Institute of Biotechnology and Pharmaceutical Research, National Health Research Institutes, Miaoli35053, Taiwan, ROC; ‡ Institute of Molecular and Genomic Medicine, National Health Research Institutes, Miaoli35053, Taiwan, ROC

## Abstract

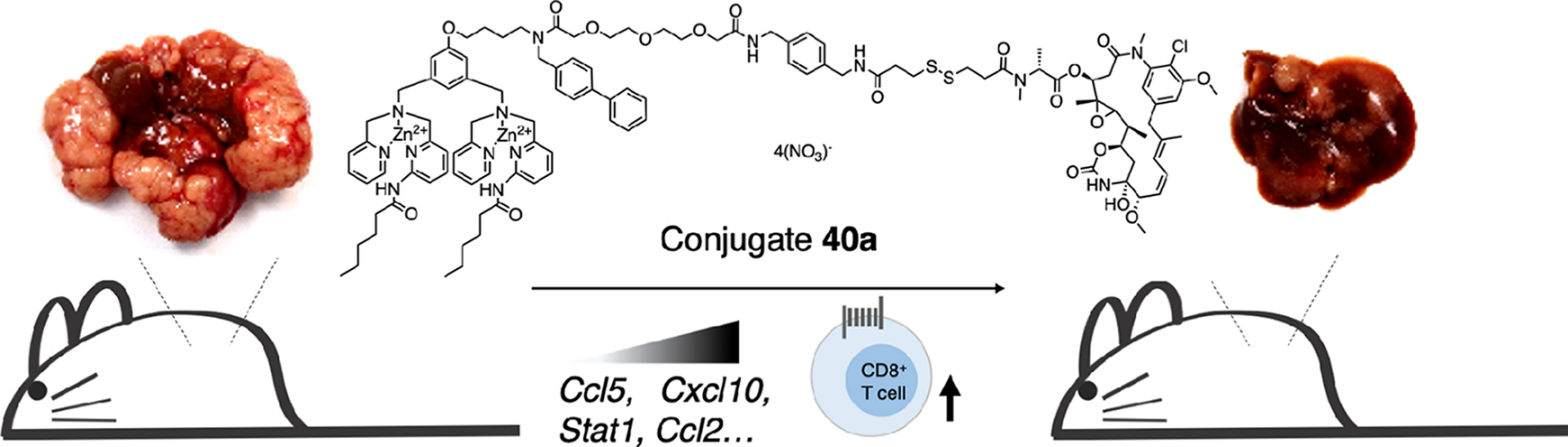

Ligand-targeting drug delivery systems have made significant strides for disease treatments with numerous clinical approvals in this era of precision medicine. Herein, we report a class of small molecule-based immune checkpoint-targeting maytansinoid conjugates. From the ligand targeting ability, pharmacokinetics profiling, *in vivo* anti-pancreatic cancer, triple-negative breast cancer, and sorafenib-resistant liver cancer efficacies with quantitative mRNA analysis of treated-tumor tissues, we demonstrated that conjugate **40a** not only induced lasting regression of tumor growth, but it also rejuvenated the once immunosuppressive tumor microenvironment to an “inflamed hot tumor” with significant elevation of gene expressions that were not accessible in the vehicle-treated tumor. In turn, the immune checkpoint-targeting small molecule drug conjugate from this work represents a new pharmacodelivery strategy that can be expanded with combination therapy with existing immune-oncology treatment options.

## Introduction

Ligand-targeted therapeutic conjugates have circumvented the pharmacokinetic limitation of the conventional chemotherapeutic agents and facilitated selective association with disease biomarkers to allow site-specific dose escalation.^1−3^ To reduce the collateral toxicity to normal cells and improve the therapeutic index of cancer chemotherapy, small molecule ligands and monoclonal antibodies have been used as a delivery moiety of cytotoxic compounds in the forms of small molecule drug conjugates (SMDCs) and antibody-drug conjugates (ADCs). Spurred by the eight marketing authorizations of ADCs for cancer therapy since 2017, ADCs have become one of the fastest-growing drug classes in oncology. However, limited penetration into solid tumor masses, high cost of goods, and premature drug release of the ADCs have motivated the SMDC development for pharmacodelivery applications as several are under clinical investigations.^4,5^ A comparative analysis between chemically defined, cell-surface antigen carbonic anhydrase IX (CAIX)-targeting ADCs and SMDCs has suggested that SMDC technology allowed efficient targeting and accumulation in the tumor mass to mediate potent antitumor effects dosing with the same drug molar ratio to that of ADCs.^6^ Concurrently, cancer immunotherapy, eradicating tumor cells via enhancing patient’s immunity, has been deployed for mono- or combination therapeutic options in treating malignant tumors.^7^ Indeed, various types of immunotherapy, including immune checkpoint inhibitors,^8^ T cell transfer therapy,^9^ and chimeric antigen receptor T-cell immunotherapy (CAR-T)^10,11^ have been successfully used for treatment. Yet, clinical evidence has provided insight into the poor prognosis associated with the degree of immune activation effects of the tumors.^12−14^ In turn, developing new immunostimulatory agents is thus essential to compensate for such deficiencies in cancer treatment. Here, we devise a new therapeutic module of the *immuno*-SMDC (*i*SMDC) that targets an immune checkpoint ligand in the tumor microenvironment (TME) and simultaneously delivers a cytotoxic compound in the form of chemically defined SMDCs.

Externalized phosphatidylserine (PS) on the tumor cells^15,16^ or tumor-derived exosomes in the TME have become a cancer diagnostic biomarker. Its inherent immunosuppressive properties have also propelled the development of several PS-targeting agents under clinical investigations.^17−20^ In addition to the conventional apoptotic function of PS,^21,22^ PS exposure on the surface of the tumor cells, through binding to TIM and TAM family proteins and Stabilin 1 or 2 receptors,^23−25^ readily suppressed immune activation of dendritic cells, macrophages, and T-cells.^26^ PS-targeting molecules, including antibodies,^27−29^ liposomes,^30^ ADCs,^31^ targeting peptide carrying paclitaxel,^32^ and SMDCs,^33−35^ have shown sound antitumor effects in preclinical mouse models and undergone evaluations in clinical trials. Herein, the development of *i*SMDCs was based on zinc(II) bis-dipicolylamine (Zn-DPA) and its derivative conjugating to maytansinoid with a hydrophilic linker. Leveraging specific interaction between the coordinated zinc ions within Zn-DPA and the anionic phosphate moieties, Zn-DPA has been employed in numerous biomedical applications.^36−41^ This particular strategy offers a multitude of antitumor growth properties: (1) highly potent maytansinoid can readily initiate apoptosis of tumor, (2) newly triggered PS externalization from tumor cell death can provide an additional homing signal to recruit circulating conjugates, and (3) binding of Zn-DPA to PS on the tumor cells can modulate PS binding to receptors of the immune cells and lead to the rejuvenation of the functionalities of the immune cells in the TME.

Moreover, since PS overexpression was found in many solid tumors, we have profiled the new maytansinoid *i*SMDC against the growth of pancreatic cancer, triple-negative breast cancer, and liver cancer. We assessed the PS-targeting ability and structure-activity relationship study via linker modifications with subsequent pharmacokinetics profiling to improve the stability and tolerability of the conjugates *in vivo*. We observed eradication of small or shrinkage of large tumor xenografts at well-tolerated doses. We then demonstrated that this maytansinoid conjugate could elicit immune cell infiltration and rejuvenate the “cold” tumor microenvironment to the inflamed “hot” tumor. The current study underscores the importance of the interplay between targeting an immune checkpoint ligand and delivery of highly potent cytotoxic in a chemically defined small organic molecule drug conjugate. Notably, it presents a new pharmacodelivery platform that, with the growing list of new small organic molecules identified as immune checkpoint inhibitors/binders, harnesses the potent activities of chemotherapy and compliments the immunostimulatory effects of cancer therapy.

## Results and Discussion

### Synthesis of the Zn-DPA Derivative and Zn-DPA Maytansinoid Conjugates

**Figure 1:**
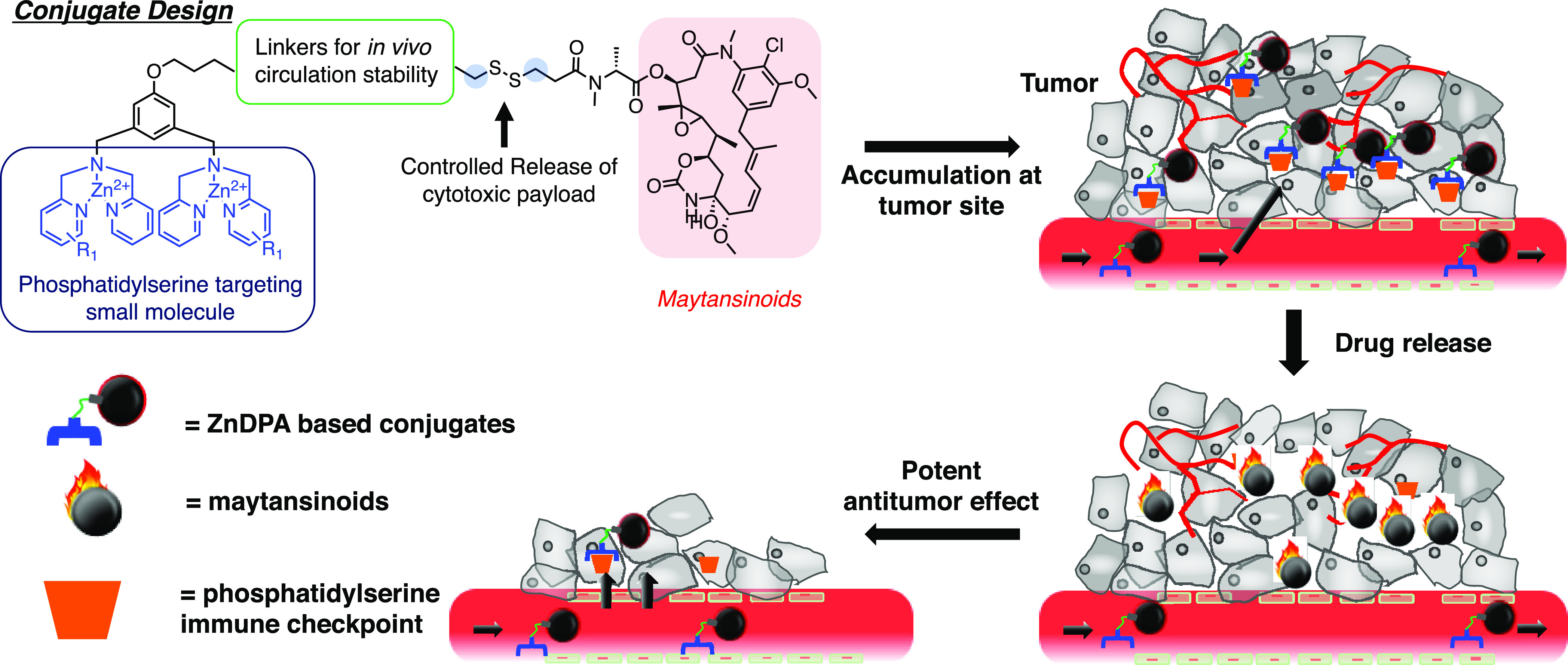
Design of small-molecule maytansinoid conjugates with active targeting of phosphatidylserine (PS) at tumor tissue and linkers that allow *in vivo* circulation stability. With the controlled release of the cytotoxic maytansinoid in the tumor microenvironment, activation of the apoptotic pathway can lead to the amplification of the homing signal *in situ* as the very exposure of PS shall facilitate recruitment of circulating conjugates that result in the enhancement of anti-tumor activities.

**Figure 2:**
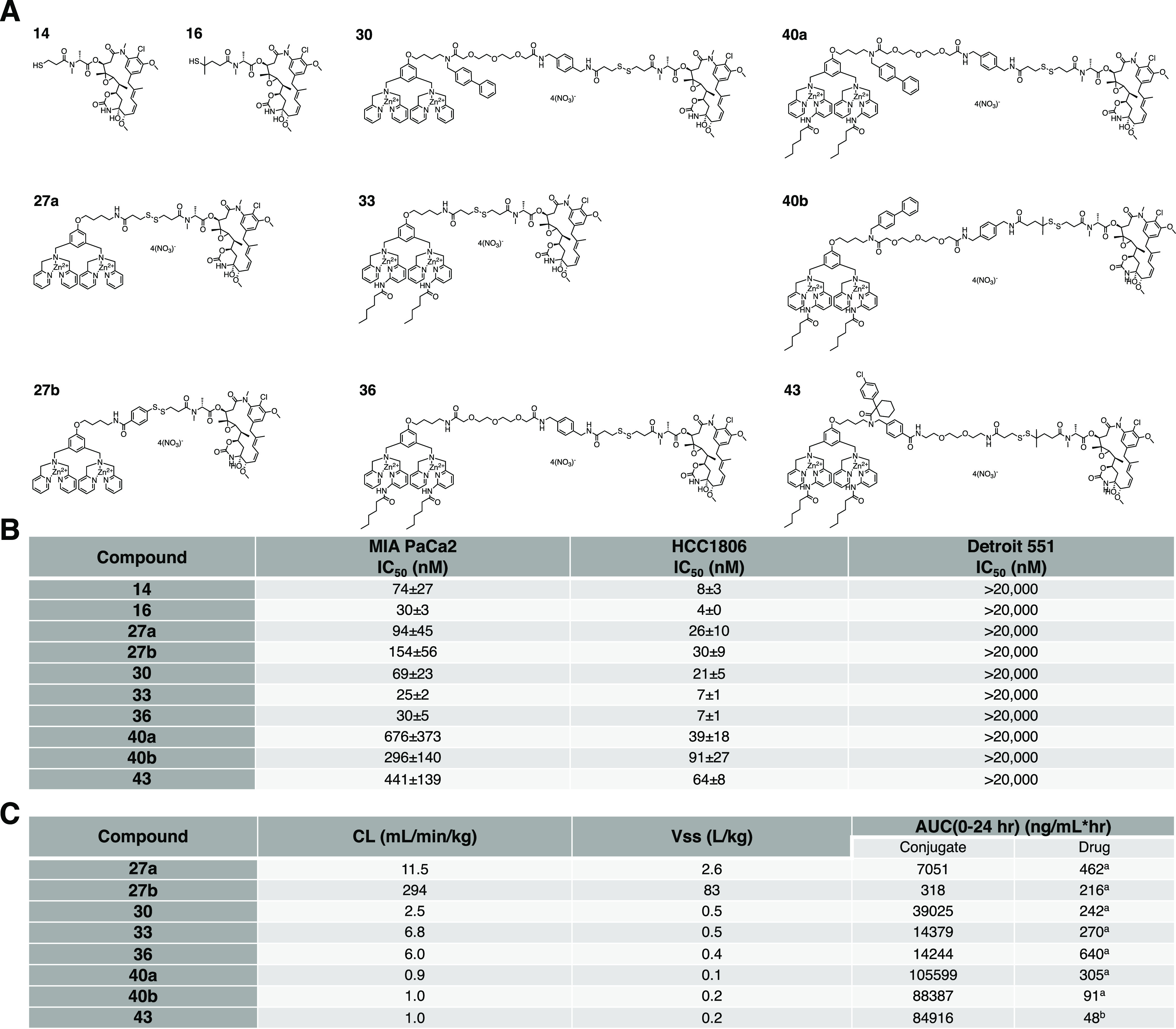
Properties of ZnDPA maytansinoid conjugates. (A) Chemical structures of the newly synthesized conjugates. (B) Cytotoxic effects on MIA PaCa-2 human pancreatic cells, HCC 1806 human triple-negative breast cancer cell, and Detroit 551 normal skin fibroblast. After a 72 h incubation of conjugates or parent cytotoxics with the cells, the ability to reduce tetrazolium compound by the viable cells was determined. (C) *In vivo* pharmacokinetic profiles of each conjugate in male ICR mice (*n* = 3) at 5 mg/kg with intravenous administration, where *a* = payload **14** and *b* = payload **16**. CL, clearance; *V*_ss_, apparent volume of distribution at steady state; AUC, area of drug concentration under the curve; mpk, mg/kg.

**Scheme 1:**
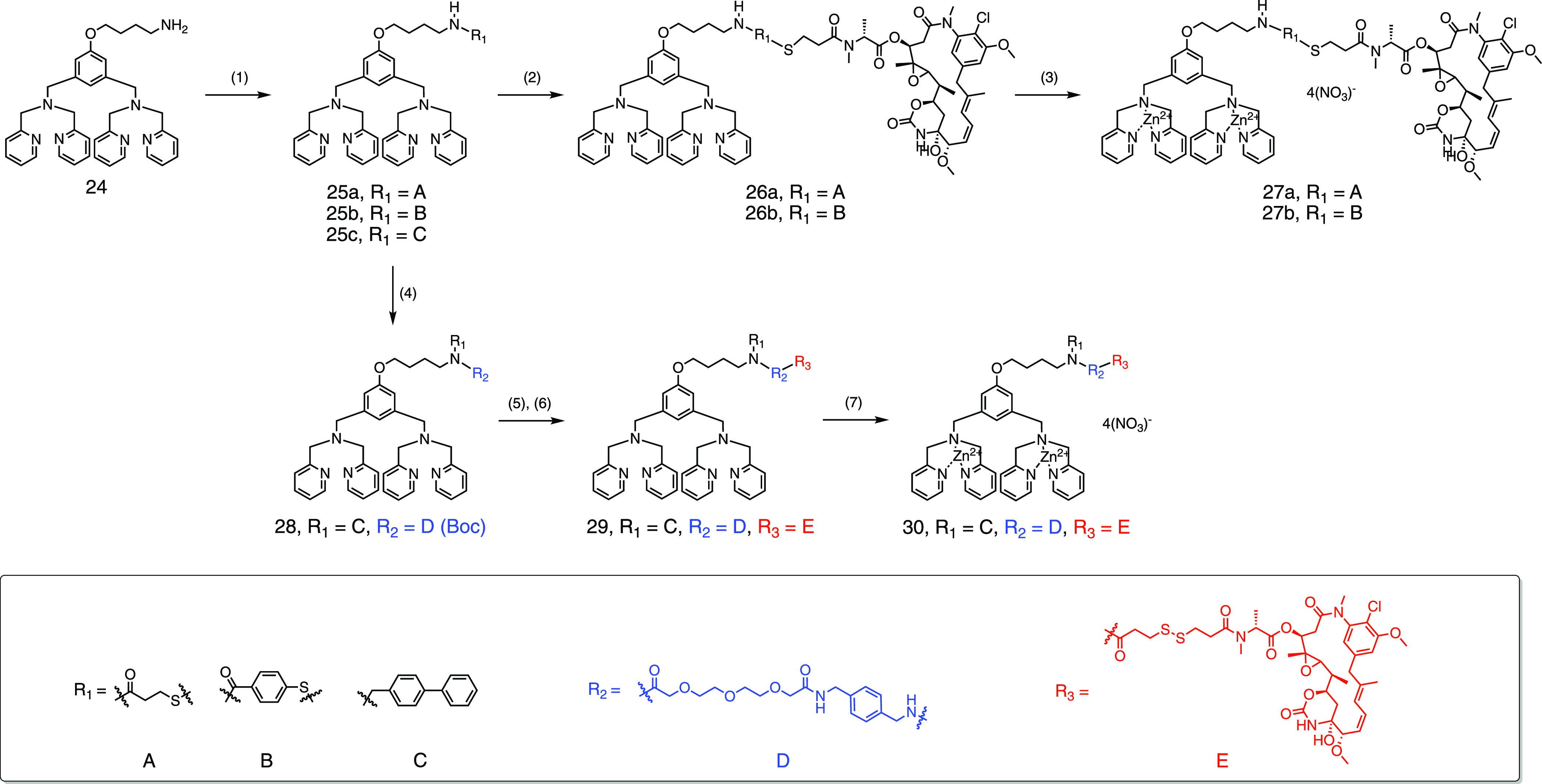
Synthetic Procedures for Zinc Dipicolylamine Maytansinoid Conjugates **27a**, **27b**, and **30** Reagents and conditions: (1) (a) compound **13**, EDCl, HOBt, NMM, CH_2_Cl_2_, 15 h, 52%; (b) compound **21**, EDCI, HOBt, DIPEA, CH_2_Cl_2_, rt, 2 h, 84%; (c) biphenyl-4-carboxaldehyde, NaBH_4_, MeOH, 70 °C, 24 h, 69%; (2) compound **14**, (a) CH_2_Cl_2_, overnight, 92%; (b) DMF, rt, 18 h, 27%; (3) 2.0 equiv. of Zn(NO_3_)_2_, CH_2_Cl_2_/MeOH; (4) compound **23**, HBTU, HOBt, NMM, 18 h, 64%; (5) TFA, CH_2_Cl_2_, 2 h; (6) compound **15**, EDCI, HOBt, NMM, 19 h, 47% in two steps; (7) 2.0 equiv. of Zn(NO_3_)_2_, CH_2_Cl_2_/MeOH.

**Scheme 2:**
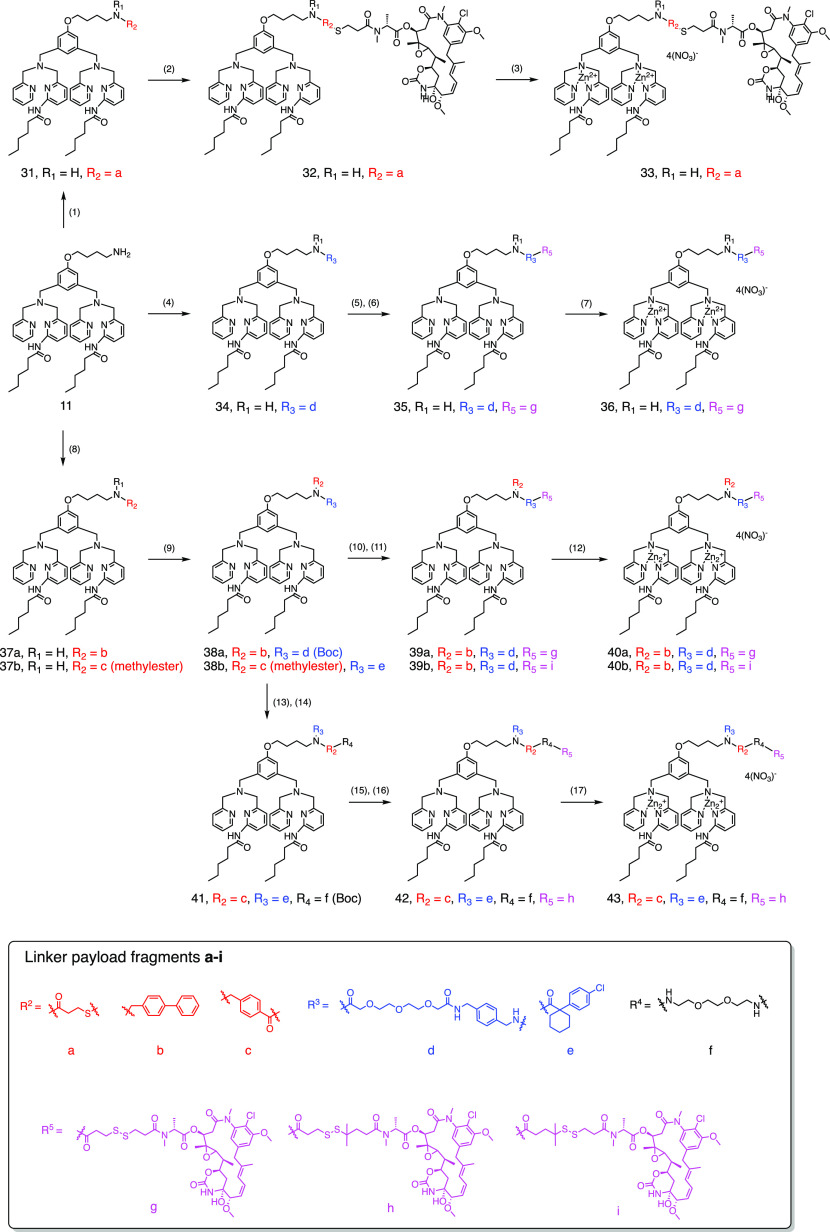
Synthetic Procedures for Modified Zinc Dipicolylamine Maytansinoid Conjugates **33**, **36**, **40a**, **40b**, and **43** Reagents and conditions: (1) compound **13**, EDCl, HOBt, NMM, CH_2_Cl_2_, 15 h, 52%; (2) compound **14**, CH_2_Cl_2_, overnight, 74%; (3) 2.0 equiv. Zn(NO_3_)_2_, CH_2_Cl_2_/MeOH; (4) compound **23**, EDCl, HOBt, NMM, CH_2_Cl_2_, 15 h, 77%; (5) TFA, CH_2_Cl_2_, 2 h; (6) compound **15**, EDCl, HOBt, NMM, CH_2_Cl_2_, overnight, 74% in two steps; (7) 2.0 equiv. Zn(NO_3_)_2_, CH_2_Cl_2_/MeOH; (8) (a) biphenyl-4-carboxaldehyde, NaBH_4_, MeOH, 70 °C, 24 h, 68%; (b) methyl 4-formylbenzoate, MeOH, 80 °C; then NaBH_4_, 0 °C, 66%; (9) (a) compound **23**, HBTU, HOBt, NMM, CH_2_Cl_2_, 18 h, 70%; (b) 1-(4-chlorophenyl)cyclohexanecarbonyl chloride, triethylamine, CH_2_Cl_2_, 60%; (10) TFA, CH_2_Cl_2_, 2 h; (11) (a) compound **15**, EDCI, HOBt, NMM, CH_2_Cl_2_, 19 h, 45% in two steps; (b) compound **19**, EDCI, HOBt, NMM, CH_2_Cl_2_, 18 h, 36% in two steps; (12) 2.0 equiv. Zn(NO_3_)_2_, CH_2_Cl_2_/MeOH; (13) 0.5 N LiOH_(aq)_, MeOH, 85%; (14) *tert*-butyl-{2-[2-(2-aminoethoxy)ethoxy]ethyl}carbamate, EDCl, HOBt, NMM, CH_2_Cl_2_, 15 h, 73%; (15) TFA, CH_2_Cl_2_, 2 h; (16) compound **17**, EDCl, HOBt, NMM, CH_2_Cl_2_, overnight, 47% in two steps; (17) 2.0 equiv. Zn(NO_3_)_2_, CH_2_Cl_2_/MeOH.

To evaluate the impacts among the targeting ligand, linker stabilities on the pharmacokinetic profiles, and *in vivo* activities of the conjugates, the Zn-DPA maytansinoid conjugates were designed and synthesized through (1) a variety of hydrophilic linkers containing ethylene glycol units, (2) installing steric hindrance with methyl groups on the adjacent carbon next to the sulfur group, (3) employing two different maytansinoids used in the clinical trials for ADCs, DM1 (compound **14**) and DM4 (compound **16**), and (4) incorporating a modified PS-targeting Zn-DPA analog. Recent studies have demonstrated that even in the absence of ligand internalization, some ADCs or SMDCs could achieve potent efficacies through efficient payload release within the tumor mass, either mediating by different extracellular proteases or by reduction of disulfide linkers.^6,42−46^ Modifications around the dipicolylamine group were shown to increase binding affinity and selectivity toward PS by taking advantage of additional secondary noncovalent interactions in hydrogen bonding and hydrophobic insertion into the membrane.^47,48^ For the synthesis of the DPA derivative, we installed a hydrophobic alkyl chain to one side of the dipicolylamine unit (Scheme S1, compound **11**). In Scheme S1, synthesis of the DPA derivative started with the coupling of hexanoic acid to methyl 6-aminopicolinate, which was then followed by the reduction of ester with NaBH_4_ to furnish compound **3**. Oxidation of compound **3** with MnO_2_ proceeded smoothly to allow the formation of aldehyde intermediate **4**, and the overall yield for these three steps was 70%. Reductive amination between 2-picolylamine and intermediate **4** was performed to obtain the alkyl-modified picolylamine precursor **5**. Treatment of 5-hydroxyisophthalate **6** with lithium aluminum hydride in dry THF at 50 °C furnished the fully reduced 3,5-bis(hydroxymethyl)phenol **7** in good yield at 97%. Alkylation of **7** with 2-(4-bromobutyl)isoindoline-1,3-dione was performed in the presence of potassium carbonate to afford the corresponding diol compound **8** in 44% yield. Following the MnO_2_ oxidation of diol **8**, the resulting dicarbaldehyde intermediate **9** was then coupled with picolylamine precursor **5,** and crude product **10** was then treated with hydrazine to furnish the Zn-DPA derivative **11** (49% yield in three steps). To probe the release of the payload, we chose to employ two different maytansinoids, DM1 (compound **14**) and DM4 (compound **16**), that have been investigated in the clinical trials for ADCs. We synthesized the caged maytansinoid precursors by introducing steric hindrance with dimethyl groups on either side of the adjacent carbon next to the disulfide linkage (Scheme S2 and Figure 1). In Scheme S2, maytansinoid precursors **15** and **17**, differing in methyl group substituents on adjacent carbons next to the sulfur, were synthesized through disulfide exchange with 3-(pyridin-2-yldisulfaneyl)propanoic acid **13** in moderate yields. In addition, maytansionoid precursor **19** was obtained by first reacting payload **14** with 2,2’-dithiobis(5-nitropyridine) followed by the treatment of intermediate **18** with 4-mercapto-4-methylpentanoic acid in THF and potassium phosphate buffer (50 mM, pH 7.5). Next, the approaches to accessing different maytansinoid conjugates linking to DPA **24** or the modified DPA derivative **11** are outlined in Scheme 1 and 2, respectively. The key step is the conjugation of different linkers between the targeting DPA moiety and the maytansinoid-containing precursors. Activation of propanoic acid in intermediate **13** with EDCl and HOBt followed by the addition of DPA **24** gave DPA-linker **25a** in 52% yield. Payload **14** (DM1) was stirred with **25a** in CH_2_Cl_2_, and the resulting conjugate **26a** was obtained in 92% yield. Disulfide containing benzoic acid **21** was activated with EDCI and HOBt followed by coupling with DPA **24** to afford **25b** in 84% yield. Conjugate **26b** was obtained via disulfide exchange between payload **14** and intermediate **25b**. To increase the solubility and stability of the eventual conjugates, we have incorporated small-unit linear ethylene glycol to bridge the derivatives of the targeting ligand and the cytotoxic payload. Large-unit pegylation could increase the solubility of the conjugate, yet the resulting steric interference might modulate the targeting ligand’s binding affinity or hinder cargo release.^49^ Reductive amination between biphenyl-4-carboxaldehyde and DPA **24** gave **25c** in 69% yield, which was then coupled with PEG-containing linker **23** (Scheme S2) to provide intermediate **28**. Removal of the Boc protecting group in **28** with TFA allowed the conjugation with maytansinoid precursor **15** to furnish conjugate **29** (47% in two steps). In Scheme 2, conjugate **32** was synthesized by coupling the activated intermediate **13** and the DPA derivative **11** followed by disulfide exchange with payload **14**. PEG-containing linker **23** was first activated by EDCI and HOBt, and DPA derivative **11** in CH_2_Cl_2_ was coupled to provide intermediate **34** in 77% yield. TFA deprotection of the Boc group in **34** followed by the conjugation with maytansinoid precursor **15** has led to the synthesis of conjugate **35** (74% in two steps). Conjugates **39a** and **39b** were synthesized via common intermediate **37a**, obtained by reductive amination of biphenyl-4-carboxaldehyde and DPA derivative **11**. Intermediate **38a**, employing the PEG-containing linker **23**, was coupled with maytansinoid precursor **15** or **19** to provide conjugate **39a** or **39b**, respectively. Alternatively, the LiOH hydrolyzed product with a 1-(4-chlorophenyl)cyclohexanecarbonyl chloride functional group in **38b_acid** was then reacted with PEG linker group f (Scheme 2) to give compound **41** in 73 % yield. After TFA deprotection of intermediate **41**, maytansinoid precursor **17** was introduced by the forming amide bond in conjugate **42**. Formation of the resulting Zn-DPA conjugates **27a**, **27b**, **30**, **33**, **36**, **40a**, **40b**, and **43** was carried out by incubating each of the DPA-maytansinoid conjugates **26a**, **26b**, **29**, **32**, **35**, **39a**, **39b**, and **42** with two equivalents of Zn(NO_3_)_2_ at room temperature, respectively. In Figure S1, comparative spectroscopic analysis and structural characterizations among conjugate **40a** and its key intermediates **39a, 38a**, and **15** were carried out to demonstrate the formation of the drug conjugate. Moreover, the chemical shifts at the dipicolylamine region after the complex formation between zinc and dipicolylamine were also observed (Figure S1). In addition, we synthesized imaging probe **Zn11-794** (Scheme S3) to address the tumor-targeting ability of the new DPA derivative **11***in vivo*. Taken together, modular constructions between different targeting ligands (DPA **24** and its derivative **11)**, linker fragments, and drug payload precursors have allowed the synthesis of a collection of conjugates (Figure 2A) for structure–activity and property relationship investigation.

### Cytotoxicities and *In Vivo* Pharmacokinetic Profiles of the Conjugates

We then examined cytotoxicities of the newly synthesized conjugates (Figure 2A) against MIA PaCa-2 (pancreatic), HCC1806 (triple-negative breast cancer) cancer cell lines, and a normal fibroblast Detroit 551 (Figure 2B). The IC_50_ of maytansinoids **14** and **16** inhibited MIA PaCa-2 and HCC1806 cancer cell growth, ranging from 70 to 4 nM. In general, the conjugates exhibited significantly less cytotoxicities toward normal Detroit 551 cells. In particular, conjugates **40a**, **40b**, and **43** harnessed prodrug properties against these cancer cell lines relative to the parent maytansinoids, suggesting that linker modifications could improve the stability and shield their cytotoxic properties *in vitro* (Figure 2B). Compared to **36**, the addition of the biphenyl group in conjugate **40a** has resulted in better stability and prodrug properties. Premature release and inadequate delivery of the cytotoxic cargo could increase off-target organ distribution and toxicities. To address the longevity of the intact conjugate during *in vivo* systemic circulation, single intravenous dose pharmacokinetic studies (Figure 2C) showed a reduction of clearance (CL) rate and volume distribution *V*_ss_ (0.2∼0.5) of conjugates **40a**, **40b**, and **43**, suggesting that pegylation of the linkers increased stability and preferential systemic distributions were in circulation. Notably, structure–property relationship studies among conjugates **30**, **36**, and **40a** have demonstrated that alkyl-chain modifications in ZnDPA analog **11** and biphenyl moiety addition in the linker region could result in a slower CL (mL/min/kg) rate (0.9 for **40a***vs* 2.5 for **30** and 6.0 for **36**) and a 5-fold decrease in volume distribution (*V*_ss_) to achieve an intact conjugate AUC of 105,599 (ng/mL hr) for **40a** (Figure 2C). For conjugates **40b** and **43**, steric hindrance introduced by the dimethyl group on the adjacent carbon next to the sulfur group of the maytansinoids has resulted in ∼20% loss of AUC. Furthermore, **40a** was highly potent against ovarian, skin, and oral cancer cell lines (Figure S2). Taken together, we identified **40a** with improved pharmacokinetic profiles and harnessed prodrug properties among the designed Zn-DPA maytansinoid conjugates.

### Tumor Targeting Ability of the Zn-DPA Derivative and Systemic Stability of Conjugate **40a**

**Figure 3:**
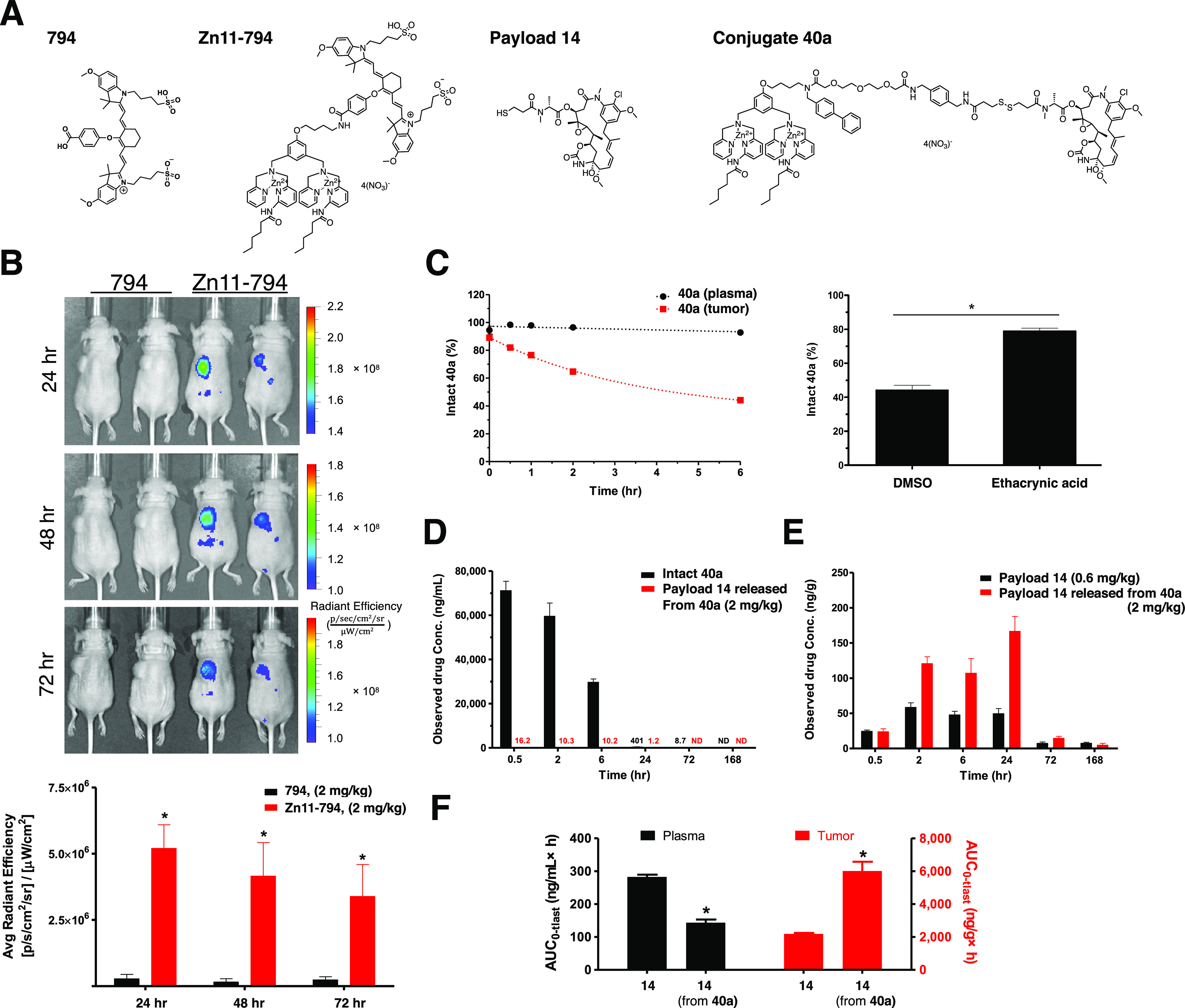
*In vivo* targeting ability, plasma stability, and *in vivo* biodistribution of modified zinc dipicolylamine conjugates in subcutaneous HCC1806 tumor xenografts. (A) Chemical structures of **dye_794**, modified ZnDPA conjugated with **dye_794**: **Zn11-794**, cytotoxic payload **14**, and conjugate **40a**. (B) *In vivo* detection of PS-expression in the HCC1806 tumor xenograft model. Representative IVIS images of new **Zn11-794** fluorescence probe in mice. Targeted with significant and lasting tumor site accumulation of **Zn11-794** were observed up to 72 h with a single intravenous dose of the conjugate at 2 mg/kg. (C) Time- and tumor homogenate-dependent cleavage of conjugate **40a** was determined. The addition of glutathione *S*-transferase (GST) inhibitor, ethacrynic acid, has rescued the cleavage of **40a**. (D) Comparison of plasma (*n* = 3) stability of intact conjugate **40a** (black bar) and the payload **14** released from **40a** (red) in HCC1806 bearing mice after a single intravenous dose (2 mg/kg) of conjugate **40a**. (E) With samples collected at indicated time points, the amount of maytansinoid **14** via direct injection (black bar) or released from **40a** (red bar) in the collected tumor tissues was determined by LC/MS/MS. (F) Plasma and tumor distribution with AUC comparisons between injection of untargeted **14** and targeted delivery of **14** in the form of conjugate **40a**.

As the Zn-DPA motif was shown to play an essential role in PS recognition,^34^ we next addressed the association properties of analog **11** with PS-containing liposomes. By using uniform-sized liposomes with 100 % DOPC as a non-specific binding control, a surface plasmon resonance (SPR) assay with PS-coated liposomes (DOPC/ DOPS (3:1,v/v)) was carried out according to the reported procedures.^35^ In comparison to the Zn-DPA compound **24**, Zn-DPA derivative **11** exhibited a relative improvement of the PS-association property (Figure S3) that resulted from a 3-fold improvement of dissociation *k*_off_ (0.00358 s^–1^) over compound **24** (0.0113 s^–1^). This result suggested that a favorable hydrophobic interaction through alkyl-chain addition to the dipicolylamine moiety in **11** had provided stronger complexation in PS-containing liposomes. Indeed, **Zn11-794**, through conjugation between analog **11** and a near-infrared dye794 (Figure 3A), showed *in vivo* HCC1806 tumor targeting ability and lasting tumor site accumulation for up to 3 days (Figure 3B). This data also demonstrated PS expression in the HCC1806 tumors. Next, we showed that conjugate **40a**, harboring analog **11** as the PS-targeting moiety, was stable in plasma incubation (Figure 3C). However, **40a** was readily cleaved with the tumor homogenates (Figure 3C). Treatment of glutathione transferase inhibitor ethacrynic acid significantly rescued the abundance of intact conjugate **40a** in the tumor homogenate, suggesting that the tumor homogenate could facilitate cleavage of the disulfide bond in **40a** (Figure 3C). Since PS lacks an internalization mechanism, we reason that the disulfide linkage can be readily cleaved in the tumor microenvironment to release maytansinoids *in vivo*. Indeed, in HCC1806 tumor-bearing mice, **40a** not only exhibited great plasma stability with negligible detection of **14** (Figure 3D), but a significant time-dependent increase of **14** released from **40a** in the tumor mass was also readily observed (Figure 3E). In all, these results demonstrated that conjugate **40a** could target and accumulate in tumor sites with limited exposure of **14** in plasma and allow controlled release of payload **14** in the tumor mass (Figure 3F).

### *In Vivo* Antitumor activities of Conjugate **40a**

**Figure 4:**
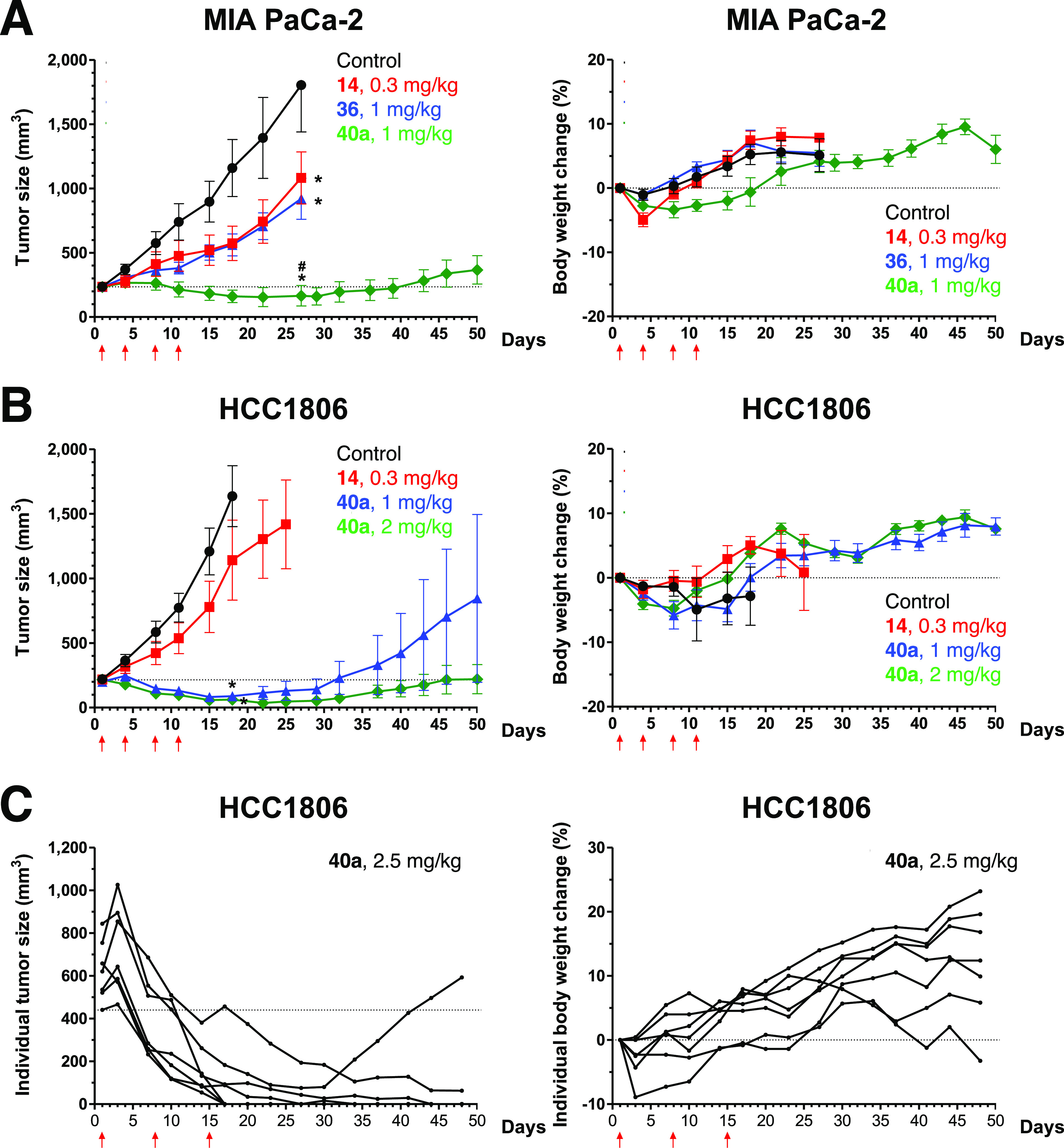
*In vivo* antitumor efficacies. The treatment regimen was presented as the amount in mg/kg and the weekly dosage frequency. The amount of the payload **14** deployed for each treatment was calculated from the percentage of **14** in the total dose of conjugate or drug used in the corresponding treatment. (A) Comparisons of anti-MIA PaCa-2 pancreatic cancer activities and body weight changes between conjugate **36**, **40a**, and cytotoxic payload **14** (at equivalent doses of **14**), when administered intravenously at a time point (twice per week), are illustrated with red arrows. (B) Comparisons of anti-HCC1806 triple-negative breast cancer activities and body weight changes between conjugate **40a** and cytotoxic payload **14** when administered intravenously at a time point (twice per week) are illustrated with red arrows. (C) Treatment and shrinkage of large (450–850 mm^3^) HCC1806 triple-negative breast cancer tumor with weekly doses of conjugate **40a** at 2.5 mg/kg.

With the determined chemical stability of conjugate **40a** and slow clearance rate during blood circulation, we evaluated the *in vivo* antitumor activities (Figure 4) in MIA PaCa-2 and HCC1806 human xenografts models. In the first set of efficacy investigations, although conjugates **27a**, **27b**, and **33** possessed low circulation exposures (Figure 2C) and still exhibited antitumor activities, significant body weight loss during treatment was readily observed (Figure S4). This data suggested that the uncontrolled or premature releases of maytansinoids might contribute to the undesired systemic toxicities. To circumvent this issue, we leveraged those respective modifications in the linker and targeting ligands of conjugates **36** and **40a** and observed potent antitumor efficacies without apparent body weight loss. These conjugates were dosed intravenously at 1 mg/kg twice weekly for two weeks (Figure 4). Comparative study based on equimolar doses of cytotoxic maytansinoid in **14** (0.3 mg/kg) and in conjugates **36** (1 mg/kg) and **40a** (1 mg/kg) was performed. The data showed that conjugate **40a**’s improved systemic exposure of the intact conjugate could provide potent and lasting anti-MIA PaCa-2 activity *in vivo* (Figure 4A). Concurrently, we have carried out a 28-day repeat dose pilot toxicity study of **40a** in rats and showed that treatment of **40a** did not alter organ weights (liver and kidneys). In general, hematologic (leukocyte, neutrophil, lymphocyte, and platelet) parameters were normal, except for a reduced erythrocyte count. Biochemical parameters, such as GOT and GPT, were slightly increased (not significant). In addition, BUN and creatinine levels were normal for the **40a** treatment group in this pilot toxicity study (Figure S5). We envision that optimizing the dosing regimen (amount and frequency) during the treatment might further expand the therapeutic window of this class of conjugates. In addition, potent antitumor efficacy was also observed against HCC1806 triple-negative breast cancer (TNBC) xenografts, indicating the benefit of targeted delivery and controlled release of the maytansinoid **14** in the form of SMDC (Figure 4B). Gratifyingly, potent efficacies and shrinkage of larger (400–800 mm^3^) HCC1806 tumors were observed when dosing at 2.5 mg/kg once per week for three weeks (Figure 4C). This study has provided another aspect of TNBC treatment, where lessening tumor burden could facilitate surgical removal procedures.

**Figure 5:**
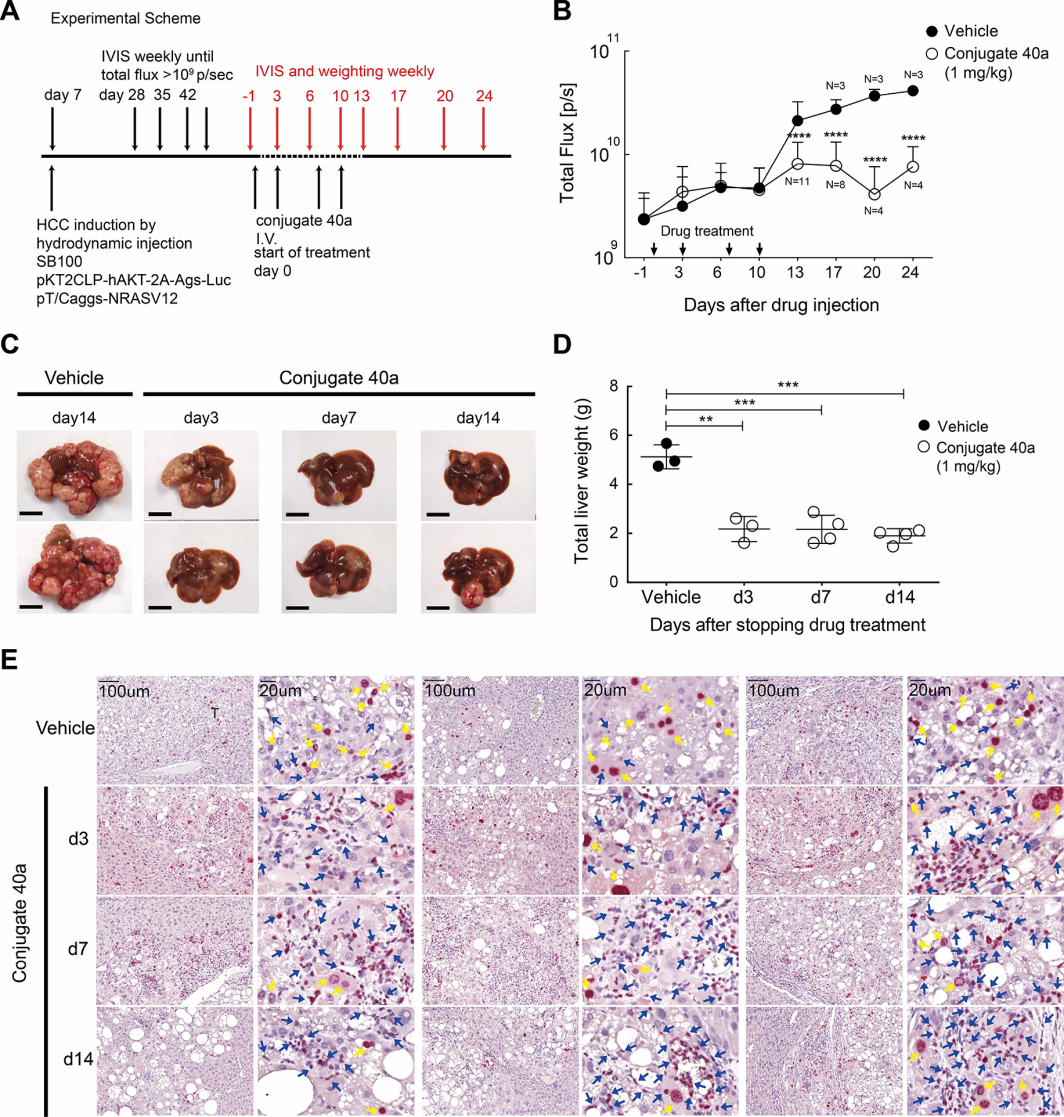
*In vivo* anti-liver cancer efficacies of conjugate **40a**. (A) Experimental scheme for HCC induction and treatment regimen. (B) Bioluminescence detection between mice treated with the vehicle and conjugate **40a** via intravenous administration at 1 mg/kg with the indicated regimen. (C) Representative images and (D) total liver weights of the vehicle (day 14)- or conjugate **40a**-treated mice livers harvested at the indicated time post drug administration. (E) Ki-67 staining of vehicle- or conjugate **40a**-treated tumor tissues at the indicated time point. A significant reduction of Ki-67 staining (yellow arrows) in the **40a**-treated tumor was observed on a scale of 100 or 20 μm.

In the second set of the efficacy tests, conjugate **40a** was evaluated in an immunocompetent, sorafenib-resistant hepatocellular carcinoma (HCC) model^50^ with a relevant expression of tumor-associated profiles in the TME. In this particular animal model, the bioluminescence reporter and luciferase activity provided noninvasive monitoring of tumor burden and progression at the liver (Figure 5A). Notably, conjugate **40a** elicited potent activity against HCC tumor growth with only 1 mg/kg injected twice weekly for two weeks (Figure 5B). Indeed, conjugate **40a**-treated livers showed a significant decrease in tumor burden examined by bioluminescence (Figure 5B), resulting in the marked reduction of total liver weight at 14 days after treatment (Figure 5C,D). In addition, the reduction of Ki67 expression in conjugate **40a**-treated tumors was observed (Figure 5E), suggesting that the treatment with **40a** could significantly diminish the proliferation of liver cancer cells and inhibit tumor growth. Overall, we have showed that conjugate **40a** not only exerted potent antipancreatic cancer and anti-triple-negative breast cancer activities but also significantly decreased HCC tumor burden in a sorafenib-resistant model.

### Profiling of Immunogenic TME and Gene Expression Induced by Conjugate **40a**

**Figure 6:**
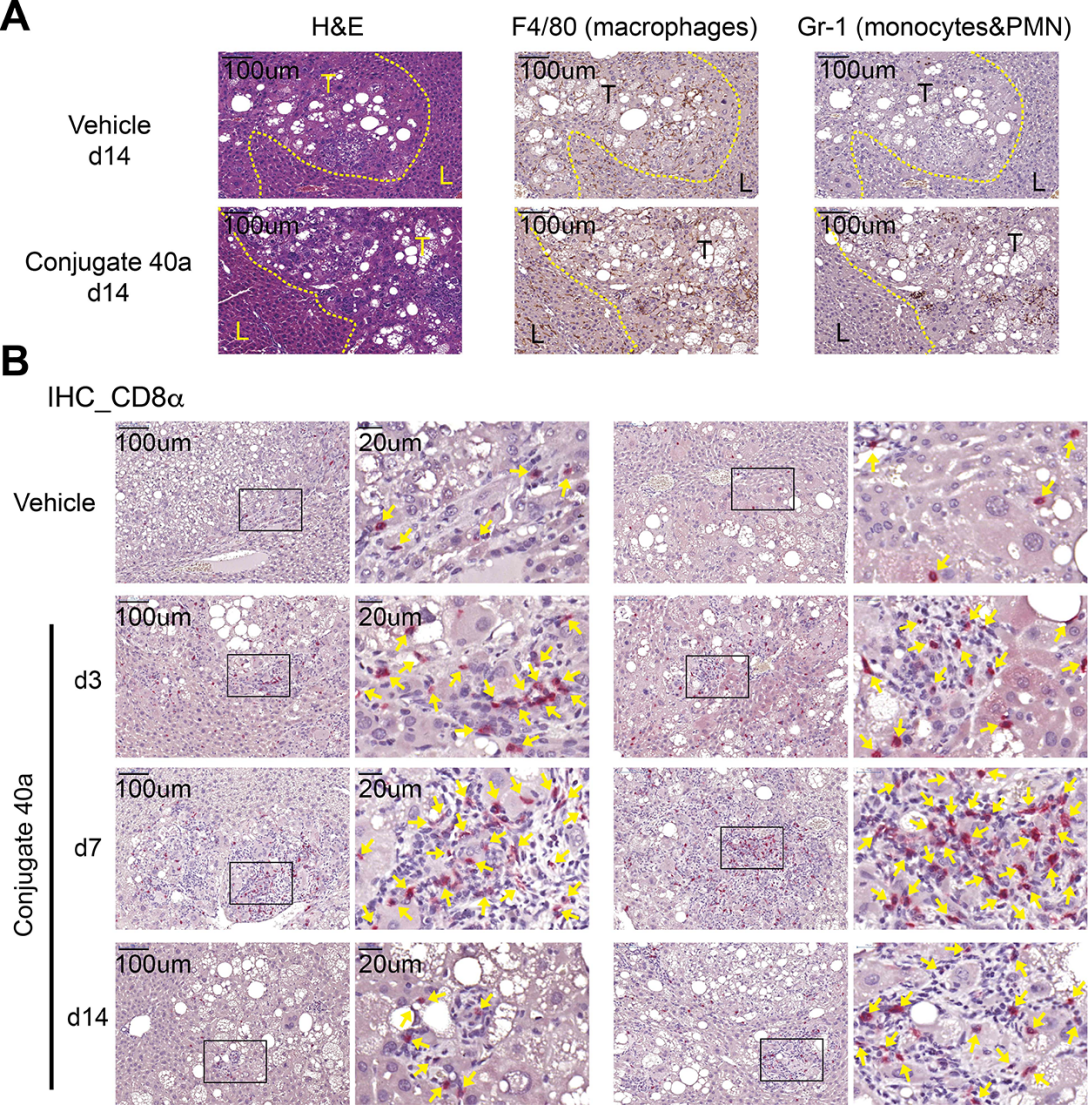
(A) Liver sections of vehicle- or conjugate **40a**-treated tissues with hematoxylin-and-eosin (H&E) staining. In addition, F4/80 and Gr-1 immunohistochemical staining. T: tumor region, L: adjacent liver tissue. (B) Immunohistochemical analysis (yellow arrows) of CD8α positive cells in vehicle- or conjugate **40a**-treated tissues. Scale bars: 100 or 20 μm.

**Figure 7:**
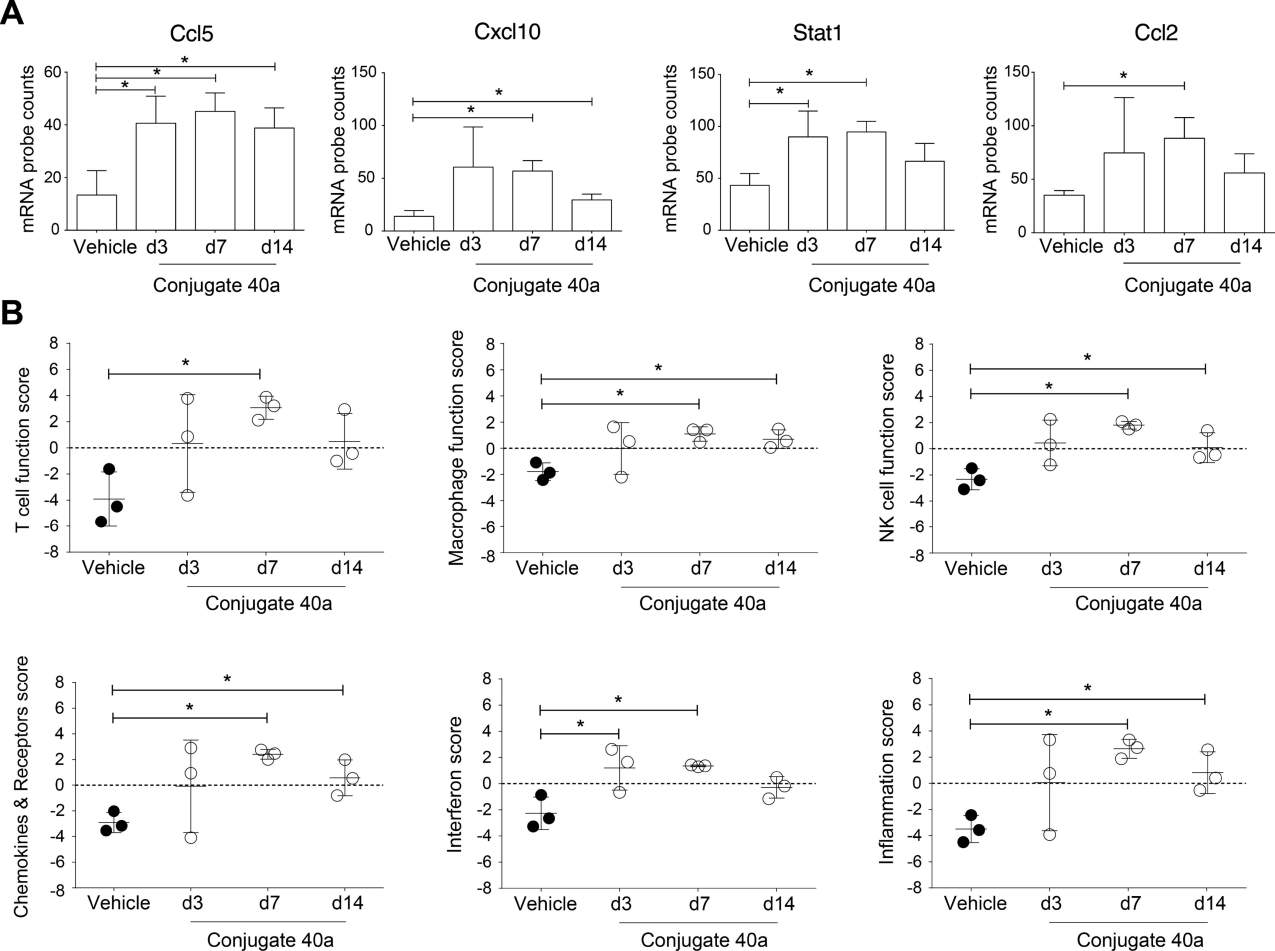
(A) Quantitative analysis of conjugate **40a**-induced immunogenic gene expression in the TME. (B) Enhancement of T cell, macrophage, NK cell, chemokine & receptor, interferon, and inflammation functions in the TME by conjugate **40a**, *n* = 3.

Limited tumor-infiltrating immune cells in immunosuppressive TME can modulate the treatment’s outcome. Many solid tumors are characterized as “cold tumors” with low proinflammatory cytokines and T-cell infiltration.^51^ On the other hand, “hot tumors” might potentiate clinical response rates of (PD-L)1/PD-1 immunotherapy and therapeutic strategies that can sensitize cold tumors into hot tumors have been investigated.^52,53^ To address the influence of conjugate **40a** treatment on the immune milieu of the HCC TME, we first analyzed conjugate **40a**-treated tumors by immunohistochemical (IHC) staining and found increased infiltration of the Gr-1^+^ monocytes and polymorphonuclear granulocytes and F4/80^+^ macrophages that formed inflammatory foci in the tumor modules (Figure 6A). Moreover, on days 3, 7, and 14, a significant increase of cytotoxic CD8^+^ T-cell infiltration was readily located in the **40a**-treated tumor (Figure 6B). These results showed that treatment of conjugate **40a** increased the permeation of multifaceted immune cells in the TME, thereby turning the HCC microenvironment into the ″hot″ status. Next, we profiled the landscape of immunogenic gene expression induced by conjugate **40a** with isolation of the total RNA from vehicle- or conjugate **40a**-treated tumor tissues. In particular, absolute copies of 700 inflammation-related mRNAs were measured. An 18-gene set of tumor inflammation signature (TIS), associated with antigen presentation, T cell/NK cell abundance, interferon activity, and T-cell exhaustion, was validated to be positively correlated to anti-PD-1 blockade responsiveness in a clinical setting.^54^ Significant increases in gene expression of stimulatory factors for inflamed tumors,^53^ such as tumor-cell-derived chemokine CC ligand 5 (CCL5) and chemokine (C-X-C motif) ligand 10 (CXCL10), were identified with the treatment of conjugate **40a** (Figure 7A). Dendritic cells (DCs) were shown to produce CXCL10 to recruit CXCR3-expressing CD8^+^ T cells to tumors, while CCL5 could provide homing signals for circulating T cells to infiltrate the tumor.^55^ In addition, as a critical regulator for inflammatory TME and T cells’ cytotoxicity, gene expression of signal transducers and activators of transcription 1 (STAT1) was significantly elevated in the **40a**-treated tumor. Gene expression of an initiator of inflammation and chemoattractant CCL2 was significantly elevated in the conjugate **40a**-treated tumors (Figure 7A). As the efficacy of immunotherapy correlates with the infiltration of cytotoxic T cells, recruitment of circulating T cells and regulated stimulations by CCL2 allowed *in situ* activation in the TME. This quantitative comparative gene set analysis has shown striking elevations of T-cell functions, macrophage functions, NK cell functions, chemokine and receptor functions, and the inflammation score in conjugate **40a**-treated tumor tissues (Figure 7B). A previous study has demonstrated that the maytansine-bearing antibody-drug conjugate induced immunogenic cell death of tumor cells, apoptosis, necrosis, and triggered the release or expression of danger-associated molecular patterns (DAMPs).^56^ These DAMPs were shown to activate innate immune cells effectively, trigger the release of cytokine and chemokines by the innate immune cells, and therefore change the immune milieu of the tumor microenvironment.^57,58^ We therefore postulated that conjugate **40a** could induce cell death of HCC cells *in vivo* and triggered the release of DAMPs, which subsequently activated local inflammation, including the recruitment and activation of macrophages and NK cells. The primary activation of innate immune cells by DAMPs further triggered the release of cytokines and chemokines, which increased the second wave of accumulation of immune cells, including CD8^+^ T cells in the tumor microenvironment. This finding provided insight into **40a** treatment in potentiating a “cold” TME to an immune-inflamed “hot” tumor state and expanding the combination treatment scope with other immunotherapeutics.

## conclusions

The SMDC is an emerging modality for the selective delivery of drug payloads. Digital and experimental analyses with “-omics” platforms generated a myriad of new disease-specific or -associated antigens. Targeted delivery via the chemically defined SMDC is largely underexplored as limited small organic ligands have been studied *in vivo* systemically when linking to conventional chemotherapeutics. This work demonstrated the design and evaluation of a pharmacokinetically optimizable and chemically defined SMDC that targets an immune checkpoint antigen in the TME. By employing ultratoxic maytansinoid payloads and leveraging an *in situ* amplification of the homing signal effect, conjugate **40a** effectively shrank the growth of many solid tumors. Moreover, CD8+ T cell infiltration significantly increased in the conjugate **40a**-treated tumor mass that sensitized tumors from the intrinsic immune-suppressive TME. A quantitative study on tumor inflammation-related mRNA expression revealed inductions of key gene expressions, such as STAT1, CXCL10, CCL5, and CCL2, and rejuvenation of TME with enhancement in T cell, macrophage, NK cell, chemokine, and cytokine functions. The current study thus established an immune checkpoint targeting conjugate enabling penetration of multifaceted immune cells into the tumor mass and potentiating new therapeutic strategies combined with immune checkpoint blockade treatment. In the current study, the synergy between the complementarity of a targeting moiety, the longevity of the conjugate with stable linkers, and the drug pharmacology is not only essential to developing effective ligand-targeted cancer therapeutics; it also offers important features for further development of a “theranostic” targeting phosphatidylserine immune checkpoint. Moreover, since revamping the TME immunity by turning it into a “hot tumor” leads to the liberation of antigens that are not initially accessible, this work can be expanded for combination therapy with existing treatment options.

## Experimental Section

### Synthesis: General

All materials used were commercially obtained and used as supplied unless otherwise noted. Reactions were performed under argon or nitrogen and monitored by analytical thin layer chromatography with glass-backed plates (5 × 10 cm) precoated with 60 F254 silica gel (supplied by Merck & Co., Inc., Whitehouse Station in Readington Township, NJ). Flame-dried glassware was cooled and used for reactions requiring anhydrous conditions under an argon or nitrogen atmosphere. The resulting chromatograms were visualized by an ultraviolet lamp (λ = 254 nm) followed by dipping in an ethanol solution of vanillin (5% w/v) containing sulfuric acid (3% v/v) or phosphomolybdic acid (2.5% w/v) after charring with a heat gun. Solvents used for reactions, including THF, diethyl ether (ether), DMF, toluene, dichloromethane, and pyridine, were dried and distilled under an argon or nitrogen atmosphere before use. Using silica gel 60 of 230–400 mesh size supplied by Merck with eluent systems given in volume/volume ratios, flash chromatography was routinely used to separate and purify product mixtures. ^1^H and ^13^C NMR spectra were collected from a Varian Mercury-300 (300 MHz), a Varian Mercury-400 (400 MHz), a Bruker Avance Neo AV4400, an AV4600, and a DMX-600 (600 MHz), with reporting of chemical shift values in ppm relative to the TMS in delta (δ) units. Multiplicities were denoted as s (singlet), br s (broad singlet), d (doublet), t (triplet), q (quartet), dd (doublet of doublets), dt (doublet of triplets), and m (multiplet). Coupling constants (*J*) were reported in Hertz. With an Agilent 1100 MSD mass spectrometer, electrospray mass spectra (ESMS) were recorded as *m/z* values, and for obtaining HRMS, a Bruker (Impact HD) Autoflex Max TOF/TOF (MALDI) was used. All test compounds with >95% purity were determined by an Agilent 1100 series HPLC system using a C18 column (Thermo Golden, 4.6 mm × 250 mm) with detailed conditions described in the Supporting Information. IUPAC nomenclature of compounds was determined with ACD/Name Pro software.

### General Procedure: Formation of Conjugates with Incubation of Zn(NO_3_)_2_

To a stirred solution of conjugate precursors (1 equiv.) in CH_2_Cl_2_ was added Zn(NO_3_)_2_ (2 equiv.) in MeOH at room temperature. The mixture was sonicated for 5 min, and then the mixture was concentrated under reduced pressure to furnish the eventual conjugates, which were HPLC-assayed to confirm the purity of >95% for animal studies.

#### *N*-[4-(3,5-bis{[bis(Pyridin-2-ylmethyl)amino]methyl}phenoxy)butyl]-3-(pyridin-2-yldisulfanyl)propanamide (**25a**)

A mixture of compound **13** (250.0 mg, 1.2 mmol, 1.1 equiv.), EDCI (333.0 mg, 1.7 mmol, 1.5 equiv.), and HOBt (235.4 mg, 1.7 mmol, 1.5 equiv.) was stirred in CH_2_Cl_2_ (10.6 mL) for 1 h at room temperature. A solution of compound **24** (620.5 mg, 1.1 mmol, 1.0 equiv.) and *N*-methylmorpholine (352.4 mg, 3.5 mmol, 3.0 equiv.) in CH_2_Cl_2_ (1.0 mL) was then added. The resultant reaction solution was stirred at room temperature for 15 h, quenched with saturated NH_4_Cl_(aq)_, dried over Na_2_SO_4_, and concentrated in vacuo. The residue was purified by flash chromatography over silica gel to give compound **25a** (434.0 mg, 52%). ^1^H NMR (400 MHz, CDCl_3_) δ 8.53–8.48 (m, 4H, CH-Py./DPA), 8.46–8.43 (m, 1H), 7.64–7.57 (m, 10H, CH-Py.+ CH-Ph./DPA), 7.15–7.05 (m, 6H, CH-Py.+ CH-Ph./DPA), 6.86–6.81 (m, 2H, CH-Ph./DPA), 6.56 (br, 1H), 4.01–3.94 (m, 2H), 3.80 (d, *J* = 3.0 Hz, 8H), 3.65 (d, *J* = 3.0 Hz, 4H), 3.40–3.33 (m, 2H), 3.10–3.04 (m, 2H), 2.62–2.55 (m, 2H), 1.86–1.81 (m, 2H). HRMS (ESI): calc. for C_44_H_49_N_8_O_2_S_2_^+^: 785.3414, found: 785.3418.

#### (1*S*,2*R*,3*S*,5*S*,6*S*,16*E*,18*Z*,20*R*,21*S*)-11-Chloro-21-hydroxy-12,20-dimethoxy-2,5,9,16-tetramethyl-8,23-dioxo-4,24-dioxa-9,22-diazatetracyclo[19.3.1.1.^10,14^0^3,5^]hexacosa-10(26),11,13,16,18-pentaen-6-yl (2*S*)-2-[{3-[(3-{[4-(3,5-bis{[bis(Pyridin-2-ylmethyl)amino]methyl}phenoxy)butyl]amino}-3-oxopropyl)disulfanyl]propanoyl}(methyl)amino]propanoate (**26a**)

To a solution of compound **25a** (150.0 mg, 0.2 mmol, 1.1 equiv.) in CH_2_Cl_2_ (3.6 mL), compound **14** (DM-1, 170.0 mg, 0.2 mmol, 1.0 equiv.) was added. The reaction solution was stirred at room temperature overnight and then concentrated in vacuo. The residue was purified by flash chromatography over silica gel to give compound **26a** (235.2 mg, 92%). ^1^H NMR (700 MHz, CDCl_3_) δ 8.43 (d, *J* = 4.8 Hz, 4H , CH-Py./DPA), 7.55 (td, *J* = 7.6, 1.8 Hz, 4H , CH-Py./DPA), 7.51 (d, *J* = 7.9 Hz, 4H , CH-Py./DPA), 7.20 (s, 1H), 7.06 (dd, *J* = 7.3, 4.9 Hz, 4H , CH-Py./DPA), 7.01 (s, 1H , CH-Ph./DPA), 6.75 (d, *J* = 8.1 Hz, 3H), 6.61 (d, *J* = 11.1 Hz, 1H), 6.57 (d, *J* = 1.8 Hz, 1H), 6.35 (dd, *J* = 15.3, 11.1 Hz, 1H), 6.20 (s, 1H), 6.00 (q, *J* = 5.6 Hz, 1H), 5.61 (dd, *J* = 15.4, 9.1 Hz, 1H), 5.26 (q, *J* = 6.9 Hz, 1H), 4.73 (dd, *J* = 12.0, 3.0 Hz, 1H), 4.23 (t, *J* = 11.3 Hz, 1H), 3.90 (d, *J* = 7.6 Hz, 4H), 3.73 (s, 7H), 3.58 (s, 4H), 3.40 (d, *J* = 9.0 Hz, 1H), 3.24 (d, *J* = 11.3 Hz, 4H), 3.15 (s, 2H), 3.05 (d, *J* = 12.8 Hz, 1H), 2.94 (d, *J* = 9.7 Hz, 1H), 2.88 (td, *J* = 12.8, 11.8, 8.0 Hz, 1H), 2.78 (d, *J* = 13.8 Hz, 4H), 2.74 (tt, *J* = 7.1, 2.9 Hz, 2H), 2.61–2.51 (m, 2H), 2.42 (t, *J* = 7.1 Hz, 2H), 2.11 (dd, *J* = 14.5, 3.0 Hz, 2H), 1.73 (dq, *J* = 11.8, 6.5 Hz, 2H), 1.62 (p, *J* = 7.3 Hz, 2H), 1.56 (s, 3H, CH_3_-alkyl/DM1), 1.51 (d, *J* = 13.5 Hz, 1H), 1.24 (d, *J* = 6.8 Hz, 3H, CH_3_-alkyl/DM1), 1.21 (d, *J* = 6.4 Hz, 3H, CH_3_-alkyl/DM1), 1.19 (d, *J* = 5.7 Hz, 2H), 0.80 (dt, *J* = 18.5, 7.1 Hz, 5H), 0.74 (s, 3H, CH_3_-alkyl/DM1). ^13^C NMR (176 MHz, CDCl_3_) δ 171.0, 170.7, 168.8, 159.8, 159.0, 156.1, 152.4, 148.9 (CH), 142.2, 141.1, 140.6, 139.3, 136.4 (CH), 133.1 (CH), 127.8 (CH), 125.2 (CH), 122.7 (CH), 121.9 (CH), 121.5 (CH), 118.7, 113.6 (CH), 113.1 (CH), 88.6 (CH), 80.7, 78.1, 74.1 (CH), 67.2 (CH_2_), 66.9 (CH), 59.9 (CH_2_), 58.4 (CH_2_), 56.5 (CH_3_), 52.7 (CH), 46.5 (CH_2_), 39.2 (CH_2_), 38.8 (CH), 36.3 (CH_2_), 35.7 (CH_2_), 35.5 (CH_3_), 33.4, 33.2, 33.1 (CH_2_), 32.4 (CH_2_), 31.5 (CH_2_), 30.9 (CH_3_), 29.6, 29.0, 26.6 (CH_2_), 26.3 (CH_2_), 22.5, 15.4 (CH_3_), 14.5 (CH_3_), 14.1, 13.4 (CH_3_), 12.1 (CH_3_). HRMS (ESI): calc. for C_74_H_91_ClN_10_NaO_12_S_2_^+^: 1433.5840, found: 1433.5859.

#### (1*S*,2*R*,3*S*,5*S*,6*S*,16*E*,18*Z*,20*R*,21*S*)-11-Chloro-21-hydroxy-12,20-dimethoxy-2,5,9,16-tetramethyl-8,23-dioxo-4,24-dioxa-9,22-diazatetracyclo[19.3.1.1.^10,14^0^3,5^]hexacosa-10(26),11,13,16,18-pentaen-6-yl (2*S*)-2-[{3-[(3-{[4-(3,5-bis{[bis(Pyridin-2-ylmethyl)amino]methyl}phenoxy)butyl]amino}-3-oxopropyl)disulfanyl]propanoyl}(methyl)amino]propanoate·2[Zn(NO_3_)_2_] (**27a**)

To a solution of zinc nitrate hexahydrate (25 mg, 0.084 mmol) in MeOH (3 mL), compound **26a** (60 mg, 0.042 mmol) in CH_2_Cl_2_ (3 mL) was added dropwise. The reaction mixture was sonicated at room temperature until all the solid was dissolved. The solvent was removed, and the resulting mixture and kept under reduced pressure until the weight was not changed to obtain the compound **27a** as a white powder (70 mg).

^1^H NMR (700 MHz, DMSO-*d*_6_) δ 8.67 (d, *J* = 5.1 Hz, 4H, CH-Py./DPA), 8.09 (dt, *J* = 8.1, 4.3 Hz, 4H, CH-Py./DPA), 8.00 (t, *J* = 5.9 Hz, 1H), 7.65 (t, *J* = 6.4 Hz, 4H, CH-Py./DPA), 7.57 (d, *J* = 7.8 Hz, 4H, CH-Py./DPA), 7.19–7.14 (m, 1H), 6.98 (s, 1H, CH-Ph./DPA), 6.89 (d, *J* = 13.8 Hz, 2H), 6.60–6.52 (m, 3H), 5.96–5.90 (m, 1H), 5.54 (dd, *J* = 14.0, 9.1 Hz, 1H), 5.30 (q, *J* = 6.8 Hz, 1H), 4.51 (dd, *J* = 12.0, 2.8 Hz, 1H), 4.34 (d, *J* = 16.2 Hz, 4H), 4.10–4.01 (m, 3H), 3.91 (s, 4H, CH_3_-alkyl/DM1), 3.86–3.75 (m, 8H), 3.49–3.48 (m, 2H), 3.23 (s, 3H, CH_3_-alkyl/DM1), 3.19 (d, *J* = 12.6 Hz, 1H), 3.16 (dd, *J* = 11.6, 4.7 Hz, 2H), 3.11 (s, 3H, CH_3_-alkyl/DM1), 2.93–2.89 (m, 1H), 2.88–2.81 (m, 2H), 2.77 (d, *J* = 9.7 Hz, 1H), 2.71 (d, *J* = 2.5 Hz, 5H), 2.60–2.55 (m, 1H), 2.42–2.34 (m, 2H), 2.03 (dd, *J* = 14.4, 2.8 Hz, 1H), 1.80–1.74 (m, 2H), 1.62–1.57 (m, 5H), 1.45 (dt, *J* = 16.8, 11.1 Hz, 2H), 1.23 (d, *J* = 13.0 Hz, 2H), 1.16 (d, *J* = 6.7 Hz, 3H, CH_3_-alkyl/DM1), 1.11 (d, *J* = 6.3 Hz, 3H, CH_3_-alkyl/DM1), 0.77 (s, 3H, CH_3_-alkyl/DM1). ^13^C NMR (176 MHz, DMSO-*d*_6_) δ 170.6, 170.4, 169.9, 168.3, 155.3, 154.4, 151.4, 147.9 (CH), 141.3, 140.8 (CH), 138.5, 133.8, 132.7 (CH), 128.6 (CH), 126.8 (CH), 125.2 (CH), 124.9 (CH), 124.7 (CH), 121.7 (CH), 117.6 (CH), 117.1, 113.9 (CH), 88.2 (CH), 80.1, 77.8 (CH), 73.2 (CH), 67.4 (CH_2_), 66.8 (CH), 60.1, 57.0 (CH_2_), 56.6 (CH_3_), 56.2 (CH_3_), 55.7 (CH_2_), 51.8 (CH), 45.5 (CH_2_), 38.1 (CH_2_), 37.5 (CH), 36.2 (CH_2_), 35.3 (CH_3_), 34.7 (CH_2_), 33.6 (CH_2_), 33.2 (CH_2_), 33.0 (CH_2_), 32.0 (CH_2_), 29.8 (CH_3_), 26.2 (CH_2_), 26.0 (CH_2_), 15.1 (CH_3_), 14.5 (CH_3_), 13.1 (CH_3_), 11.4 (CH_3_). HRMS (ESI): calc. for C_74_H_92_ClN_10_O_12_S_2_^+^: 1787.4116, found: 1787.3925.

#### *N*-[4-(3,5-bis{[bis(Pyridin-2-ylmethyl)amino]methyl}phenoxy)butyl]-4-(pyridin-2-yldisulfanyl)benzamide (**25b**)

To a solution of compound **21** (136 mg, 0.517 mmol) in CH_2_Cl_2_ (9 mL) at room temperature, 1-(3-dimethylaminopropyl)-3-ethylcarbodiimide hydrochloride (EDCI, 135 mg, 0.704 mmol) followed by hydroxybenzotriazole (HOBt, 95 mg, 0.704 mmol) was added. After the reaction mixture was stirred for 1 h, compound **24** (276 mg, 0.470 mmol) and *N*,*N*’-diisopropylethylamine (DIPEA, 0.25 mL, 1.409 mmol) were added consecutively; then, the reaction mixture was stirred at room temperature for 1 h. After the reaction was completed, 2N HCl_(aq)_ was poured into the reaction mixture and then the mixture was adjusted to pH 2–3. The aqueous phase was neutralized with saturated NaHCO_3(aq)_ and extracted with CH_2_Cl_2_ (10 mL × 3). The combined organic layers were dried over Na_2_SO_4_, filtered, and concentrated in vacuo. The residue was purified by flash chromatography with 3% MeOH in CH_2_Cl_2_ to yield the compound **25b** (147 mg, 84%). ^1^H NMR (400 MHz, CDCl_3_) δ 8.51–8.48 (m, 4H, CH-Py./DPA), 8.48–8.45 (m, 1H), 7.69–7.48 (m, 15H), 7.14–7.09 (m, 5H), 7.04 (br, 1H), 6.84 (d, *J* = 1.4 Hz, 2H), 4.01 (t, *J* = 5.8 Hz, 2H), 3.79 (s, 8H, CH_2_-αPh./DPA), 3.63 (s, 4H CH_2_-αPh./DPA), 3.52 (d, *J* = 6.2 Hz, 2H), 1.90–1.78 (m, 4H). ^13^C NMR (151 MHz, CDCl_3_) δ 166.9, 159.8, 159.1, 159.0, 149.8 (CH), 149.0 (CH), 140.7, 140.3, 137.5 (CH), 136.6 (CH), 133.4, 127.8 (CH), 126.6 (CH), 122.9 (CH), 122.1 (CH), 121.8 (CH), 121.3 (CH), 119.8 (CH), 113.7 (CH), 67.5 (CH_3_), 60.2 (CH_3_), 58.6 (CH_3_), 39.9 (CH_3_), 26.8 (CH_3_), 26.6 (CH_3_). HRMS (ESI): calc. for C_48_H_48_N_8_NaO_2_S_2_^+^: 855.3234, found: 855.3237.

#### (1*S*,2*R*,3*S*,5*S*,6*S*,16*E*,18*Z*,20*R*,21*S*)-11-Chloro-21-hydroxy-12,20-dimethoxy-2,5,9,16-tetramethyl-8,23-dioxo-4,24-dioxa-9,22-diazatetracyclo[19.3.1.1.^10,14^0^3,5^]hexacosa-10(26),11,13,16,18-pentaen-6-yl (2*S*)-2-[{3-[(4-{[4-(3,5-bis{[bis(Pyridin-2-ylmethyl)amino]methyl}phenoxy)butyl]carbamoyl}phenyl)disulfanyl]propanoyl}(methyl)amino]propanoate (**26b**)

To a solution of compound **25b** (32 mg, 0.039 mmol) in anhydrous DMF (0.77 mL) at room temperature, compound **14** (DM-1, 30 mg, 0.040 mmol) was added. After stirring at room temperature for 18 h, DMF was removed. The residue was purified by flash chromatography with 9% MeOH in CH_2_Cl_2_ to yield the compound **26b** (15 mg, 27%). ^1^H NMR (700 MHz, CDCl_3_) δ 8.45–8.41 (m, 4H, CH-Py./DPA), 7.58–7.53 (m, 6H, CH-Py.+ CH-Ph./DPA), 7.50 (d, *J* = 7.8 Hz, 4H, CH-Py./DPA), 7.31–7.27 (m, 2H), 7.07–7.04 (m, 4H, CH-Py./DPA), 7.00 (s, 1H), 6.78 (d, *J* = 1.5 Hz, 2H), 6.68 (d, *J* = 1.8 Hz, 1H), 6.60–6.54 (m, 2H), 6.48 (d, *J* = 1.8 Hz, 1H), 6.33 (dd, *J* = 15.4, 11.2 Hz, 1H), 6.19–6.17 (m, 1H), 5.59 (dd, *J* = 15.3, 9.1 Hz, 1H), 5.25 (q, *J* = 7.0 Hz, 1H), 4.71 (dd, *J* = 12.1, 3.0 Hz, 1H), 4.20–4.15 (m, 1H), 3.95 (t, *J* = 6.0 Hz, 2H), 3.90 (s, 3H, CH_3_-alkyl/DM1), 3.73 (s, 8H, CH_2_-αPh./DPA), 3.58 (s, 4H, CH_2_-αPh./DPA), 3.49–3.44 (m, 3H), 3.40 (d, *J* = 9.1 Hz, 1H), 3.25 (s, 3H, CH_3_-alkyl/DM1), 3.11 (s, 3H, CH_3_-alkyl/DM1), 2.96–2.90 (m, 3H), 2.88–2.83 (m, 1H), 2.71 (s, 3H, CH_3_-alkyl/DM1), 2.69 (dd, *J* = 8.4, 6.1 Hz, 1H), 2.64–2.58 (m, 1H), 2.50 (dd, *J* = 14.5, 12.1 Hz, 1H), 2.08 (dd, *J* = 14.5, 3.1 Hz, 2H), 1.84–1.79 (m, 2H), 1.77 (q, *J* = 7.1 Hz, 2H), 1.54 (s, 3H, CH_3_-alkyl/DM1), 1.51–1.47 (m, 1H), 1.40–1.35 (m, 1H), 1.23 (d, *J* = 6.9 Hz, 3H, CH_3_-alkyl/DM1), 1.19 (d, *J* = 6.5 Hz, 3H, CH_3_-alkyl/DM1), 0.82–0.76 (m, 1H), 0.71 (s, 3H, CH_3_-alkyl/DM1). ^13^C NMR (176 MHz, CDCl_3_) δ 170.6, 170.6, 168.9, 166.9, 159.7, 159.1, 156.0, 152.4, 149.0 (CH), 142.1, 141.1, 140.8, 140.6, 139.3, 136.6 (CH), 133.3 (CH), 133.1, 127.9 (CH), 127.7 (CH), 126.0 (CH), 125.4 (CH), 122.9 (CH), 122.1 (CH), 122.0 (CH), 121.8 (CH), 118.8, 113.8 (CH), 113.3 (CH), 88.7 (CH), 80.9 (CH), 78.2, 74.2, 67.5 (CH_2_), 67.1 (CH), 60.1, 58.7, 56.7 (CH_3_), 52.7, 46.7 (CH_2_), 39.9 (CH_2_), 38.9 (CH), 36.4 (CH_2_), 35.6 (CH_3_), 33.4 (CH_2_), 32.5 (CH_2_), 31.0, 26.8 (CH_2_), 26.5 (CH_2_), 15.6 (CH_3_), 14.7 (CH_3_), 13.6 (CH_3_), 12.3 (CH_3_). HRMS (ESI): calc. for C_78_H_91_ClN_10_NaO_12_S_2_^+^: 1481.5840, found: 1481.5833.

#### (1*S*,2*R*,3*S*,5*S*,6*S*,16*E*,18*Z*,20*R*,21*S*)-11-Chloro-21-hydroxy-12,20-dimethoxy-2,5,9,16-tetramethyl-8,23-dioxo-4,24-dioxa-9,22-diazatetracyclo[19.3.1.1.^10,14^0^3,5^]hexacosa-10(26),11,13,16,18-pentaen-6-yl (2*S*)-2-[{3-[(4-{[4-(3,5-bis{[bis(Pyridin-2-ylmethyl)amino]methyl}phenoxy)butyl]carbamoyl}phenyl)disulfanyl]propanoyl}(methyl)amino]propanoate·2[Zn(NO_3_)_2_] (**27b**)

To a solution of zinc nitrate hexahydrate (25 mg, 0.084 mmol) in MeOH (3 mL), compound **26b** (61 mg, 0.042 mmol) in CH_2_Cl_2_ (3 mL) was added dropwise. The reaction mixture was sonicated at room temperature until all the solid was dissolved. The resulting mixture was removed and kept under reduced pressure until the weight was not changed to obtain the compound **27b** as a white powder (92 mg, quant).

^1^H NMR (700 MHz, DMSO-*d*_6_) δ 8.67 (d, *J* = 5.2 Hz, 4H, CH-Py./DPA), 8.58 (t, *J* = 5.8 Hz, 1H), 8.12–8.07 (m, 4H, CH-Py./DPA), 7.78 (d, *J* = 8.1 Hz, 2H), 7.65 (t, *J* = 6.5 Hz, 4H, CH-Py./DPA), 7.57 (d, *J* = 7.8 Hz, 4H, CH-Py./DPA), 7.42 (d, *J* = 8.0 Hz, 2H), 7.08–7.05 (m, 1H), 7.02 (s, 1H, CH-Ph./DPA), 6.89 (s, 2H, CH-Ph./DPA), 6.54 (d, *J* = 12.8 Hz, 2H), 6.45 (d, *J* = 1.8 Hz, 1H), 5.93 (s, 1H), 5.59–5.53 (m, 1H), 5.28 (q, *J* = 6.8 Hz, 1H), 4.50 (dd, *J* = 11.9, 2.8 Hz, 1H), 4.35 (d, *J* = 16.0 Hz, 3H), 4.16–4.10 (m, 2H), 4.07–4.02 (m, 1H), 3.91 (s, 3H, CH_3_-alkyl/DM1), 3.85 (s, 3H, CH_3_-alkyl/DM1), 3.78 (d, *J* = 16.0 Hz, 4H), 3.48 (d, *J* = 9.0 Hz, 1H), 3.24 (s, 3H, CH_3_-alkyl/DM1), 3.16 (d, *J* = 5.2 Hz, 1H), 3.11 (d, *J* = 12.5 Hz, 1H), 3.02 (s, 3H, CH_3_-alkyl/DM1), 2.76 (d, *J* = 9.7 Hz, 1H), 2.63 (s, 3H, CH_3_-alkyl/DM1), 2.52–2.48 (m, 7H), 2.08 (s, 3H), 2.03–1.98 (m, 1H), 1.84 (q, *J* = 7.1 Hz, 2H), 1.79–1.72 (m, 2H), 1.56 (s, 3H, CH_3_-alkyl/DM1), 1.44 (dd, *J* = 11.3, 4.4 Hz, 1H), 1.23 (dd, *J* = 9.7, 5.2 Hz, 2H), 1.15 (d, *J* = 6.8 Hz, 3H, CH_3_-alkyl/DM1), 1.10 (d, *J* = 6.3 Hz, 3H), 0.74 (s, 3H, CH_3_-alkyl/DM1). ^13^C NMR (176 MHz, DMSO*d*_6_) δ 170.5, 168.2, 165.5, 158.9, 155.2, 154.4, 151.3, 147.9 (CH), 141.2, 140.8 (CH), 138.3, 133.7, 132.8, 132.6 (CH), 128.6 (CH), 128.0 (CH), 125.4 (CH), 125.2 (CH), 124.9 (CH), 124.7 (CH), 121.4 (CH), 117.7 (CH), 117.1, 113.8 (CH), 88.2 (CH), 80.0, 77.7 (CH), 73.2 (CH), 67.4 (CH_2_), 66.8 (CH), 60.0, 57.0 (CH_2_), 56.5 (CH_3_), 56.2 (CH_3_), 55.7 (CH_2_), 55.0 (CH_2_), 51.7 (CH), 45.7 (CH2),38.9 (CH_2_), 37.5 (CH), 36.4 (CH_2_), 35.1 (CH_3_), 33.9 (CH_2_), 32.9 (CH_2_), 31.9 (CH_2_), 30.7, 29.7 (CH_3_), 26.3, 26.0, 15.0 (CH_3_), 14.4 (CH_3_), 13.1 (CH_3_), 11.4 (CH_3_). HRMS (ESI): calc. for C_78_H_90_ClN_14_O_24_S_2_Zn_2_^–^: 1833.3971, found: 1833.3980.

#### *N*-(Biphenyl-4-ylmethyl)-4-(3,5-bis{[bis(pyridin-2-ylmethyl)amino]methyl}phenoxy)butan-1-amine (**25c**)

To a solution of compound **24** (2.00 g, 3.403 mmol) in MeOH (34 mL) at room temperature, biphenyl-4-carboxaldehyde (1.24 g, 6.806 mmol) was added. The reaction solution was slowly warmed to 70 °C and stirred for 20 h. The solution was cooled down to 0 °C, and then sodium borohydride (0.52 g, 13.611 mmol) was slowly added. The reaction was slowly warmed to room temperature and stirred for 4 h, and then saturated NH_4_Cl_(aq)_ was poured into the reaction mixture. After MeOH was removed, the residue was extracted with CH_2_Cl_2_ (50 mL × 2). The combined organic layers were dried over Na_2_SO_4_, filtered, and concentrated in vacuo. The residue was purified by flash chromatography with 5% MeOH in CH_2_Cl_2_ to yield the compound **25c** (1.78 g, 69%). ^1^H NMR (600 MHz, CDCl_3_) δ 8.51–8.49 (m, 4H, CH-Py./DPA), 7.62–7.54 (m, 13H), 7.44–7.41 (m, 2H), 7.41–7.38 (m, 2H), 7.35–7.31 (m, 1H), 7.13–7.10 (m, 4H, CH-Py./DPA), 7.06 (t, *J* = 1.5 Hz, 1H), 6.86 (d, *J* = 1.4 Hz, 2H), 3.97 (t, *J* = 6.4 Hz, 2H), 3.85 (s, 2H), 3.80 (s, 8H, CH_2_-αPh./DPA), 3.65 (s, 4H, CH_2_-αPh./DPA), 2.76–2.72 (m, 2H), 1.87–1.83 (m, 2H), 1.75–1.69 (m, 2H). ^13^C NMR (151 MHz, CDCl_3_) δ 159.9, 159.3, 149.1 (CH), 141.1, 140.7, 140.0, 139.6, 136.5 (CH), 128.9 (CH), 128.7 (CH), 127.2 (CH), 127.1 (CH), 122.8 (CH), 122.0 (CH), 121.5 (CH), 113.6 (CH), 67.8 (CH_2_), 60.2 (CH_2_), 58.7 (CH_2_), 53.8 (CH_2_), 49.3 (CH_2_), 27.3 (CH_2_), 26.9 (CH_2_). HRMS (ESI): calc. for C_49_H_51_N_7_NaO^+^: 776.4047, found: 776.4062.

#### *tert*-Butyl-{4-[14-(biphenyl-4-ylmethyl)-18-(3,5-bis{[bis(pyridin-2-ylmethyl)amino]methyl}phenoxy)-3,13-dioxo-5,8,11-trioxa-2,14-diazaoctadec-1-yl]benzyl}carbamate (**28**)

To a solution of compound **23** (159 mg, 0.360 mmol) in CH_2_Cl_2_ (5 mL) at room temperature, *O*-(benzotriazol-1-yl)-*N*,*N*,*N*’,*N*’-tetramethyluronium hexafluorophosphate (HBTU, 273 mg, 0.721 mmol) was added followed by hydroxybenzotriazole (HOBt, 97 mg, 0.721 mmol). After the reaction mixture was stirred for 1 h, compound **25c** (181 mg, 0.240 mmol) and *N*-methylmorpholine (NMM, 0.16 mL, 1.441 mmol) were added consecutively, and then the reaction was stirred for 17 h. After the reaction was completed, the reaction mixture was quenched with saturated NH_4_Cl_(aq)_ at ice-bath temperature and then adjusted to pH 6–7. Organic volatiles were evaporated, and then the residue was partitioned into H_2_O and CH_2_Cl_2_. The aqueous phase was extracted with CH_2_Cl_2_ (5 mL × 3), and the combined organic layers were dried over Na_2_SO_4_, filtered, and concentrated in vacuo. The residue was purified by flash chromatography (5% MeOH in CH_2_Cl_2_) to yield the compound **28** (181 mg, 64%). ^1^H NMR (400 MHz, CDCl_3_) δ 8.52–8.47 (m, 4H, CH-Py./DPA), 7.64–7.48 (m, 13H), 7.47–7.29 (m, 4H, CH-Py./DPA), 7.26–7.19 (m, 4H, CH-Py./DPA), 7.15–7.07 (m, 4H, CH-Py./DPA), 6.82 (d, *J* = 1.4 Hz, 2H), 4.59 (d, *J* = 38.8 Hz, 2H), 4.44 (t, *J* = 6.0 Hz, 2H), 4.26 (d, *J* = 6.3 Hz, 2H), 4.17 (d, *J* = 29.0 Hz, 2H), 4.03 (d, *J* = 8.2 Hz, 2H), 3.93 (d, *J* = 5.3 Hz, 2H), 3.80 (s, 8H, CH_2_-αPh./DPA), 3.71–3.43 (m, 14H), 1.75 (dt, *J* = 7.5, 3.9 Hz, 4H), 1.44 (d, *J* = 2.1 Hz, 9H). ^13^C NMR (151 MHz, CDCl_3_) δ 169.9, 169.4, 169.1, 159.6, 159.6, 159.1, 156.0, 149.0 (CH), 140.6, 140.5, 140.5, 140.3, 138.2, 137.4, 137.3, 136.5 (CH), 129.0 (CH), 128.9 (CH), 128.7 (CH), 128.2 (CH), 128.0 (CH),127.7 (CH), 127.7 (CH), 127.4 (CH), 127.1 (CH), 126.9 (CH), 122.9 (CH), 122.1 (CH), 121.8 (CH), 121.5 (CH), 113.6 (CH), 79.4, 71.1 (CH_3_), 70.6 (CH_3_), 70.6 (CH_3_), 70. (CH_3_)5, 70.4 (CH_3_), 70.4 (CH_3_), 70.2 (CH_3_), 70.1, (CH_3_), 69.8, 67.4 (CH_3_), 67.2 (CH_3_), 60.0 (CH_3_), 58.6 (CH_3_), 49.8 (CH_3_), 47.8 (CH_3_), 45.9 (CH_3_), 44.3 (CH_3_), 42.5 (CH_3_), 28.4 (CH_2_), 26.8, 26.6, 25.3, 24.2. HRMS (ESI): calc. for C_70_H_82_N_9_O_8_^+^: 1176.6281, found: 1176.6313.

#### (1*S*,2*R*,3*S*,5*S*,6*S*,16*E*,18*Z*,20*R*,21*S*)-11-Chloro-21-hydroxy-12,20-dimethoxy-2,5,9,16-tetramethyl-8,23-dioxo-4,24-dioxa-9,22-diazatetracyclo[19.3.1.1.^10,14^0^3,5^]hexacosa-10(26),11,13,16,18-pentaen-6-yl (2*S*)-2-[(3-{[3-({4-[14-(Biphenyl-4-ylmethyl)-18-(3,5-bis{[bis(pyridin-2-ylmethyl)amino]methyl}phenoxy)-3,13-dioxo-5,8,11-trioxa-2,14-diazaoctadec-1-yl]benzyl}amino)-3-oxopropyl]disulfanyl}propanoyl)(methyl)amino]propanoate (**29**)

To a solution of compound **28** (181 mg, 0.154 mmol) in CH_2_Cl_2_ (1.5 mL) at room temperature, TFA (1.5 mL) was added and then the reaction mixture was stirred for 2 h. The excess amount of TFA was removed under reduced pressure, and the resulting Boc-deprotected compound was used for the next reaction without further purification. A solution of compound **15** (90 mg, 0.107 mmol) in CH_2_Cl_2_ (2 mL) at room temperature was added EDCI (31 mg, 0.160 mmol) followed by HOBt (22 mg, 0.160 mmol). After the reaction mixture was stirred for 1 h, the resulting Boc-deprotected compound (100 mg, 0.093 mmol) and NMM (0.14 mL, 1.282 mmol) were added consecutively, and then the reaction mixture was stirred for 18 h. After the reaction was completed, the resultant mixture was quenched with saturated NH_4_Cl_(aq)_ and extracted with CH_2_Cl_2_ (5 mL × 2). The organic extracts were dried over Na_2_SO_4_, filtered, and concentrated in vacuo. The residue was purified by flash chromatography (9% MeOH in CH_2_Cl_2_) to yield the compound **29** (84 mg, 47% in two steps). ^1^H NMR (700 MHz, CDCl_3_) δ 8.44–8.40 (m, 4H, CH-Py./DPA), 7.55–7.42 (m, 13H), 7.35 (dt, *J* = 16.2, 7.6 Hz, 2H), 7.28 (dd, *J* = 16.7, 7.8 Hz, 1H), 7.21 (d, *J* = 7.8 Hz, 1H), 7.14 (q, *J* = 8.0, 6.9 Hz, 4H, CH-Py./DPA), 7.08–7.02 (m, 4H, CH-Py./DPA), 6.75 (dd, *J* = 5.7, 2.8 Hz, 4H), 6.60 (d, *J* = 11.1 Hz, 1H), 6.56 (dd, *J* = 4.2, 1.8 Hz, 1H), 6.34 (dd, *J* = 15.4, 11.1 Hz, 1H), 6.19 (s, 1H), 5.62–5.56 (m, 1H), 5.27–5.23 (m, 1H), 4.74–4.69 (m, 1H), 4.51 (d, *J* = 60.5 Hz, 2H), 4.34 (dt, *J* = 34.9, 6.3 Hz, 4H), 4.24–4.19 (m, 1H), 4.03 (d, *J* = 48.6 Hz, 2H), 3.94 (d, *J* = 12.8 Hz, 2H), 3.89 (d, *J* = 2.1 Hz, 3H, CH_3_-alkyl/DM1), 3.86 (q, *J* = 5.5 Hz, 2H), 3.73 (s, 8H, CH_2_-αPh./DPA), 3.60–3.46 (m, 10H), 3.45–3.35 (m, 5H), 3.23 (d, *J* = 6.7 Hz, 3H, CH_3_-alkyl/DM1), 3.19 (t, *J* = 7.2 Hz, 1H), 3.14 (d, *J* = 3.7 Hz, 3H, CH_3_-alkyl/DM1), 3.03 (dd, *J* = 12.7, 3.2 Hz, 1H), 2.93 (d, *J* = 9.7 Hz, 1H), 2.87 (td, *J* = 9.0, 7.2, 3.0 Hz, 1H), 2.77 (d, *J* = 7.1 Hz, 4H), 2.60–2.44 (m, 4H), 2.10 (dd, *J* = 14.4, 3.0 Hz, 2H), 1.67 (d, *J* = 8.1 Hz, 4H), 1.56 (s, 3H, CH_3_-alkyl/DM1), 1.21 (d, *J* = 6.4 Hz, 6H), 0.82–0.75 (m, 6H), 0.73 (s, 3H, CH_3_-alkyl/DM1). ^13^C NMR (176 MHz, CDCl_3_) δ 171.0, 170.7, 170.2, 168.8, 159.6, 156.1, 152.4, 149.1, 148.9 (CH), 142.2, 141.1, 140.5, 139.3, 137.6, 136.5 (CH), 133.1 (CH), 128.9 (CH), 128.8 (CH), 128.5 (CH), 128.3 (CH), 128.1 (CH), 128.0 (CH), 127.9 (CH), 127.6 (CH),127.4 (CH), 127.3 (CH), 127.0 (CH), 125.8 (CH), 125.3 (CH), 122.8 (CH), 122.0 (CH), 121.6 (CH), 121.4 (CH), 118.9, 113.5 (CH), 113.2 (CH), 88.7 (CH), 80.9, 78.1 (CH), 74.1 (CH), 71.0 (CH_2_), 70.4 (CH_2_), 70.3 (CH_2_), 70.1 (CH_2_), 69.8 (CH_2_), 67.3 (CH_2_), 67.1 (CH), 61.8 (CH_2_), 60.4 (CH_2_), 59.9 (CH_2_), 58.6 (CH_2_), 58.6, 56.7, 56.5 (CH_3_), 52.6 (CH), 49.9 (CH_2_), 47.9 (CH_2_), 46.6 (CH_2_), 46.0 (CH_2_), 45.9 (CH_2_) 43.2 (CH_2_), 42.5 (CH_2_), 42.6, 38.8 (CH), 36.4 (CH_2_), 35.6 (CH_3_), 33.4 (CH_2_), 33.1 (CH_2_), 32.4 (CH_2_), 31.7, 30.9 (CH_3_), 26.9, 26.7, 25.4, 24.3, 22.8, 21.0 (CH_3_), 20.4 (CH_3_), 19.1 (CH_3_), 15.5 (CH_3_), 14.6 (CH_3_), 14.2 (CH_3_), 13.5, 13.4 (CH_3_), 12.1 (CH_3_). HRMS (ESI): calc. for C_103_H_123_ClN_12_NaO_17_S_2_^+^: 1921.8151, found: 1921.8259.

#### (1*S*,2*R*,3*S*,5*S*,6*S*,16*E*,18*Z*,20*R*,21*S*)-11-Chloro-21-hydroxy-12,20-dimethoxy-2,5,9,16-tetramethyl-8,23-dioxo-4,24-dioxa-9,22-diazatetracyclo[19.3.1.1.^10,14^0^3,5^]hexacosa-10(26),11,13,16,18-pentaen-6-yl (2*S*)-2-[(3-{[3-({4-[14-(Biphenyl-4-ylmethyl)-18-(3,5-bis{[bis(pyridin-2-ylmethyl)amino]methyl}phenoxy)-3,13-dioxo-5,8,11-trioxa-2,14-diazaoctadec-1-yl]benzyl}amino)-3-oxopropyl]disulfanyl}propanoyl)(methyl)amino]propanoate·2[Zn(NO_3_)_2_] (**30**)

To a solution of zinc nitrate hexahydrate (24 mg, 0.080 mmol) in MeOH (5 mL), compound **29** (77 mg, 0.040 mmol) in CH_2_Cl_2_ (5 mL) was added dropwise. The reaction mixture was sonicated at room temperature until all the solid was dissolved. The resulting mixture was removed and kept under reduced pressure until the weight was not changed. Compound **30** was obtained as a white powder (92 mg, quant).

^1^H NMR (700 MHz, DMSO-*d*_6_) δ 8.69 (s, 4H, CH-Py./DPA), 8.37 (d, *J* = 6.8 Hz, 1H), 8.20 (dd, *J* = 21.0, 6.3 Hz, 1H), 8.09 (q, *J* = 7.8 Hz, 4H, CH-Py./DPA), 7.69–7.51 (m, 14H), 7.44 (dt, *J* = 12.9, 7.6 Hz, 2H), 7.34 (d, *J* = 7.9 Hz, 3H), 7.16 (dd, *J* = 11.8, 7.0 Hz, 5H), 7.00 (s, 1H), 6.88 (d, *J* = 10.5 Hz, 2H), 6.58 (t, *J* = 11.2 Hz, 1H), 6.54 (d, *J* = 5.5 Hz, 1H), 5.93 (s, 1H), 5.55 (dd, *J* = 14.7, 9.0 Hz, 1H), 5.31 (q, *J* = 6.9 Hz, 1H), 4.60 (d, *J* = 15.6 Hz, 2H), 4.52 (dd, *J* = 12.0, 2.8 Hz, 1H), 4.32–4.18 (m, 10H), 4.06 (t, *J* = 11.5 Hz, 2H), 3.92–3.87 (m, 6H), 3.63–3.45 (m, 13H), 3.24 (s, 3H, CH_3_-alkyl/DM1), 3.18 (d, *J* = 12.5 Hz, 1H), 3.11 (s, 3H, CH_3_-alkyl/DM1), 2.93–2.78 (m, 4H), 2.71 (s, 3H, CH_3_-alkyl/DM1), 2.60–2.55 (m, 1H), 2.44–2.36 (m, 2H), 2.03 (dd, *J* = 14.6, 2.9 Hz, 1H), 1.72 (d, *J* = 36.2 Hz, 4H), 1.58 (s, 3H, CH_3_-alkyl/DM1), 1.46 (dd, *J* = 17.3, 9.1 Hz, 2H), 1.28–1.21 (m, 5H), 1.17 (d, *J* = 6.8 Hz, 3H, CH_3_-alkyl/DM1), 1.11 (d, *J* = 6.3 Hz, 3H, CH_3_-alkyl/DM1), 0.85 (t, *J* = 6.8 Hz, 2H), 0.77 (s, 3H, CH_3_-alkyl/DM1). ^13^C NMR (176 MHz, DMSO-*d*_6_) δ 170.6, 170.3, 169.9, 169.3, 168.2, 155.3, 154.4, 151.3, 148.0 (CH), 141.3, 141.2, 140.9 (CH), 139.8, 138.4, 138.0, 137.9, 132.6 (CH), 129.0 (CH), 128.9 (CH), 128.5 (CH), 128.1 (CH), 127.5 (CH), 127.4 (CH), 127.2 (CH), 127.2 (CH), 127.0 (CH), 126.7 (CH), 126.6 (CH), 126.6 (CH), 125.2 (CH), 124.9 (CH), 124.7 (CH), 121.6 (CH), 117.1, 113.9 (CH), 88.2 (CH), 80.0, 77.7 (CH), 73.2 (CH), 70.3 (CH_2_), 70.3 (CH_2_), 70.0 (CH_2_), 69.9 (CH_2_), 69.7 (CH_2_), 69.6 (CH_2_), 69.5 (CH_2_), 69.3 (CH_2_), 67.4 (CH_2_), 66.8 (CH), 60.0, 56.5 (CH_3_), 56.2 (CH_3_), 55.7 (CH_2_), 51.7 (CH), 49.0 (CH_2_), 47.2 (CH_2_), 45.5 (CH_2_), 41.9 (CH_2_), 41.4 (CH_2_), 37.7 (CH_2_), 36.2 (CH_2_), 35.2 (CH_3_), 34.7 (CH_2_), 33.4 (CH_2_), 33.2 (CH_2_), 32.9 (CH_2_), 32.0 (CH_2_), 31.0 (CH_2_), 29.8 (CH_3_), 26.1 (CH_2_), 26.1 (CH_2_), 24.8 (CH_2_), 23.6 (CH_2_), 22.1 (CH_2_), 15.1 (CH_3_), 14.4 (CH_3_), 14.0, 13.1 (CH_3_), 11.4 (CH_3_). HRMS (ESI): *m*/*z* calc. for C_103_H_122_ClN_16_O_29_S_2_Zn_2_^–^: 2278.6267, found: 2278.6511.

#### *N*,*N*’-([5-(4-{[3-(Pyridin-2-yldisulfanyl)propanoyl]amino}butoxy)benzene-1,3-diyl]bis{methanediyl[(pyridin-2-ylmethyl)imino]methanediylpyridine-6,2-diyl})dihexanamide (**31**)

A mixture of compound **13** (145.5 mg, 0.7 mmol, 1.1 equiv.), EDCI (193.8 mg, 1.0 mmol, 1.5 equiv.), and HOBt (137.0 mg, 1.0 mmol, 1.5 equiv.) was stirred in CH_2_Cl_2_ (6.0 mL) for 1 h at room temperature. To the reaction mixture, a solution of compound **11** (500.2 mg, 0.6 mmol, 1.0 equiv.) and *N*-methylmorpholine (205.1 mg, 2.0 mmol, 3.0 equiv.) in CH_2_Cl_2_ (1.0 mL) was added. The resultant solution was stirred at room temperature for 15 h, quenched with saturated NH_4_Cl_(aq)_, dried over Na_2_SO_4_, and concentrated in vacuo. Purification of the crude residue by flash chromatography on silica gel was eluted with MeOH/DCM (5/95) to give compound **31** (320.3 mg, 52%). ^1^H NMR (400 MHz, CDCl_3_) δ 8.86 (s, 2H, CH-Py./DPA), 8.49 (dd, *J* = 4.9, 1.6 Hz, 2H, CH-Py./DPA), 8.44 (dt, *J* = 4.8, 1.4 Hz, 1H), 8.12 (d, *J* = 8.3 Hz, 2H, CH-Py./DPA), 7.67 (t, *J* = 7.9 Hz, 2H, CH-Py./DPA), 7.62–7.53 (m, 4H, CH-Ph. + CH-Py./DPA), 7.50 (d, *J* = 7.8 Hz, 2H, CH-Py./DPA), 7.29–7.24 (m, 3H), 7.16–7.05 (m, 3H, CH-Ph. + CH-Py./DPA), 6.72 (s, 2H, CH-PH./DPA), 6.56 (br, 1H), 3.95 (t, *J* = 6.0 Hz, 2H), 3.78 (s, 4H, CH_2_-αPh./DPA), 3.70 (s, 4H, CH_2_-αPh./DPA), 3.58 (s, 4H, CH_2_-αPh./DPA), 3.36 (q, *J* = 6.6 Hz, 2H), 3.06 (t, *J* = 6.7 Hz, 2H), 2.58 (t, *J* = 6.7 Hz, 2H), 2.06 (t, *J* = 7.6 Hz, 4H), 1.82–1.80 (m, 2H), 1.75–1.69 (m, 2H), 1.59–1.49 (m, 4H), 1.26–1.10 (m, 8H), 0.82 (t, *J* = 6.9 Hz, 6H, CH_3_-alkyl/DPA). HRMS (ESI): calc. for C_56_H_71_N_10_O_4_S_2_^+^: 1011.5096, found: 1011.5101.

#### (1*S*,2*R*,3*S*,5*S*,6*S*,16*E*,18*Z*,20*R*,21*S*)-11-Chloro-21-hydroxy-12,20-dimethoxy-2,5,9,16-tetramethyl-8,23-dioxo-4,24-dioxa-9,22-diazatetracyclo[19.3.1.1.^10,14^0^3,5^]hexacosa-10(26),11,13,16,18-pentaen-6-yl (2*S*)-2-[{3-[(3-{[4-(3,5-bis{[{[6-(Hexanoylamino)pyridin-2-yl]methyl}(pyridin-2-ylmethyl)amino]methyl}phenoxy)butyl]amino}-3-oxopropyl)disulfanyl]propanoyl}(methyl)amino]propanoate (**32**)

To a solution of compound **31** (160.2 mg, 0.2 mmol, 1.0 equiv.) in CH_2_Cl_2_ (4.8 mL), compound **14** (DM-1, 175.4 mg, 0.2 mmol, 1.5 equiv.) was added. The reaction solution was stirred at room temperature overnight and then concentrated in vacuo. Purification of the crude residue by flash chromatography on silica gel was eluted with MeOH/DCM (5/95) to give compound **32** (192.6 mg, 74%). ^1^H NMR (700 MHz, CDCl_3_) δ 8.86 (s, 2H, CH-Py./DPA), 8.42 (d, *J* = 4.9 Hz, 2H, CH-Py./DPA), 8.06 (d, *J* = 8.2 Hz, 2H, CH-Py./DPA), 7.60 (t, *J* = 7.9 Hz, 2H, CH-Py./DPA), 7.50 (t, *J* = 7.7 Hz, 2H, CH-Py./DPA), 7.43 (d, *J* = 7.9 Hz, 2H, CH-Py./DPA), 7.23–7.15 (m, 4H, CH-Ph. + CH-Py./DPA), 7.07 (dd, *J* = 7.4, 5.0 Hz, 2H), 6.76 (s, 1H, CH-Ph./DPA), 6.64 (s, 2H, CH-Ph./DPA), 6.60 (d, *J* = 11.2 Hz, 1H), 6.56 (s, 1H), 6.35 (dd, *J* = 15.4, 11.1 Hz, 1H), 6.27 (s, 1H), 5.95 (t, *J* = 5.9 Hz, 1H), 5.59 (dd, *J* = 15.4, 9.0 Hz, 1H), 5.25 (q, *J* = 7.4, 6.8 Hz, 1H), 4.73 (dd, *J* = 12.0, 3.0 Hz, 1H), 4.23 (t, *J* = 11.4 Hz, 1H), 3.90 (s, 3H, CH_3_-alkyl/DM1), 3.86 (t, *J* = 6.2 Hz, 2H), 3.72 (s, 3H, CH_3_-alkyl/DM1), 3.63 (s, 3H, CH_3_-alkyl/DM1), 3.57 (d, *J* = 12.7 Hz, 1H), 3.51 (s, 3H, CH_3_-alkyl/DM1), 3.40 (d, *J* = 9.1 Hz, 1H), 3.15 (s, 3H, CH_3_-alkyl/DM1), 3.04 (d, *J* = 12.7 Hz, 1H), 2.94 (d, *J* = 9.7 Hz, 1H), 2.88 (dt, *J* = 16.8, 7.9 Hz, 1H), 2.79 (s, 3H, CH_3_-alkyl/DM1), 2.77–2.72 (m, 3H), 2.61–2.51 (m, 2H), 2.42 (t, *J* = 7.1 Hz, 2H), 2.11 (dd, *J* = 14.4, 3.0 Hz, 1H), 2.02 (t, *J* = 7.7 Hz, 4H), 1.74–1.69 (m, 2H), 1.61 (q, *J* = 7.3 Hz, 2H), 1.56 (s, 3H, CH_3_-alkyl/DM1), 1.53–1.45 (m, 6H), 1.39 (d, *J* = 6.4 Hz, 1H), 1.25–1.09 (m, 20H), 0.83–0.78 (m, 3H), 0.76 (t, *J* = 7.2 Hz, 6H, CH_3_-alkyl/DPA), 0.73 (s, 3H, CH_3_-alkyl/DM1). ^13^C NMR (176 MHz, CDCl_3_) δ 172.3, 171.0, 170.8, 170.7, 168.9, 159.7, 158.8, 157.9, 156.1, 152.5, 151.5, 148.9 (CH), 142.3, 141.1, 140.2, 139.4, 139.1 (CH), 136.7 (CH), 133.3 (CH), 128.0 (CH), 125.4 (CH), 123.0 (CH), 122.3 (CH), 122.2 (CH), 119.0 (CH), 118.9 (CH), 114.0 (CH), 113.3 (CH), 112.5 (CH), 88.8 (CH), 80.9, 78.2 (CH), 74.2 (CH), 67.4 (CH_2_), 67.2 (CH), 60.2 (CH_2_), 60.1 (CH_2_), 59.5 (CH_2_), 58.2 (CH_2_), 56.7 (CH_3_), 56.7 (CH_3_), 52.9 (CH), 46.7 (CH_2_), 39.4 (CH_2_), 39.0 (CH), 37.5 (CH_2_), 36.5 (CH_2_), 35.9 (CH_2_), 35.7 (CH_3_), 33.6 (CH_2_), 33.4 (CH_2_), 33.3 (CH_2_), 32.6 (CH_2_), 31.4 (CH_2_), 31.2 (CH_3_), 29.8 (CH_2_), 26.8 (CH_2_), 26.5 (CH_2_), 25.0 (CH_2_), 22.4 (CH_2_), 15.6 (CH_3_), 14.7 (CH_3_), 14.0 (CH_3_), 13.6 (CH_3_), 12.3 (CH_3_). HRMS (ESI): calc. for C_86_H_114_ClN_12_O_14_S_2_^+^: 1637.7702, found: 1637.7724.

#### (1*S*,2*R*,3*S*,5*S*,6*S*,16*E*,18*Z*,20*R*,21*S*)-11-Chloro-21-hydroxy-12,20-dimethoxy-2,5,9,16-tetramethyl-8,23-dioxo-4,24-dioxa-9,22-diazatetracyclo[19.3.1.1.^10,14^0^3,5^]hexacosa-10(26),11,13,16,18-pentaen-6-yl (2*S*)-2-[{3-[(3-{[4-(3,5-bis{[{[6-(Hexanoylamino)pyridin-2-yl]methyl}(pyridin-2-ylmethyl)amino]methyl}phenoxy)butyl]amino}-3-oxopropyl)disulfanyl]propanoyl}(methyl)amino]propanoate·2[Zn(NO_3_)_2_] (**33**)

^1^H NMR (700 MHz, DMSO-*d*_6_) δ 8.49–8.41 (m, 2H, CH-Py./DPA), 8.09–7.98 (m, 6H, CH-Ph. + CH-Py./DPA), 7.60 (t, *J* = 6.5 Hz, 2H, CH-Py./DPA), 7.51 (t, *J* = 6.8 Hz, 2H, CH-Py./DPA), 7.32 (t, *J* = 6.9 Hz, 2H, CH-Py./DPA), 7.18 (d, *J* = 7.4 Hz, 6H), 6.90 (s, 1H, CH-Ph./DPA), 6.57 (t, *J* = 7.6 Hz, 1H), 6.55–6.53 (m, 1H), 5.92 (s, 1H), 5.54 (dd, *J* = 14.1, 9.1 Hz, 1H), 5.30 (q, *J* = 6.8 Hz, 1H), 4.51 (dd, *J* = 12.0, 2.8 Hz, 1H), 4.43 (d, *J* = 15.6 Hz, 3H), 4.11 (s, 3H), 4.07–4.03 (m, 2H), 4.01–3.96 (m, 2H), 3.92 (s, 3H, CH_3_-alkyl/DM1), 3.78 (d, *J* = 15.6 Hz, 2H), 3.71 (d, *J* = 15.6 Hz, 2H), 3.48 (t, *J* = 10.8 Hz, 2H), 3.24 (s, 3H, CH_3_-alkyl/DM1), 3.19 (d, *J* = 12.5 Hz, 1H), 3.15 (q, *J* = 6.9 Hz, 2H), 3.11 (s, 3H, CH_3_-alkyl/DM1), 2.92 (dd, *J* = 13.1, 6.5 Hz, 1H), 2.89–2.80 (m, 3H), 2.77 (d, *J* = 9.7 Hz, 2H), 2.71 (d, *J* = 5.5 Hz, 7H), 2.60–2.55 (m, 1H), 2.40–2.35 (m, 2H), 2.03 (dd, *J* = 14.5, 2.8 Hz, 1H), 1.83–1.80 (m, 3H), 1.78–1.76 (m, 2H), 1.58 (s, 3H, CH_3_-alkyl/DM1), 1.47 (d, *J* = 12.7 Hz, 1H), 1.41 (s, 6H), 1.23 (d, *J* = 8.1 Hz, 3H), 1.17 (d, *J* = 6.8 Hz, 3H, CH_3_-alkyl/DM1), 1.11 (d, *J* = 6.4 Hz, 3H, CH_3_-alkyl/DM1), 0.94 (d, *J* = 7.5 Hz, 6H, CH_3_-alkyl/DPA), 0.88–0.81 (m, 4H), 0.77 (s, 3H, CH_3_-alkyl/DM1). ^13^C NMR (176 MHz, DMSO-*d*_6_) δ 178.5, 170.6, 170.3, 169.9, 168.2, 158.9, 155.3, 154.1, 152.4, 151.3, 147.1 (CH), 142.7 (CH), 141.3, 140.6 (CH), 138.4, 133.8, 132.6 (CH), 128.5 (CH), 127.0 (CH), 125.2 (CH), 124.8 (CH), 124.4 (CH), 121.7 (CH), 120.6 (CH), 117.8 (CH), 117.1, 114.7 (CH), 114.0 (CH), 88.2 (CH), 80.0, 77.7 (CH), 73.2 (CH), 67.4 (CH_2_), 66.8 (CH), 60.1, 58.6 (CH_2_), 56.9 (CH_2_), 56.6 (CH_3_), 56.2 (CH_3_), 55.6 (CH_2_), 51.7 (CH), 45.5 (CH_2_), 38.3 (CH_2_), 37.7 (CH), 36.9, 36.4, 35.2 (CH_3_), 34.9 (CH_2_), 33.6 (CH_2_), 33.2 (CH_2_), 32.9 (CH_2_), 32.0 (CH_2_), 31.0 (CH_2_), 30.8 (CH_2_), 29.8, 29.0, 26.2 (CH_2_), 26.0 (CH_2_), 24.6 (CH_2_), 21.9 (CH_2_), 15.1 (CH_3_), 14.5 (CH_3_), 14.0 (CH_3_), 13.1 (CH_3_), 11.4 (CH_3_). HRMS (ESI): calc. for C_86_H_112_ClN_16_O_26_S_2_Zn_2_^-^: 2011.5652, found: 2011.5636.

#### *tert*-Butyl-{4-[18-(3,5-bis{[{[6-(hexanoylamino)pyridin-2-yl]methyl}(pyridin-2-ylmethyl)amino]methyl}phenoxy)-3,13-dioxo-5,8,11-trioxa-2,14-diazaoctadec-1-yl]benzyl}carbamate (**34**)

A mixture of compound **23** (121.7 mg, 0.3 mmol, 1.5 equiv.), EDCI (79.3 mg, 0.4 mmol, 1.5 equiv.), and HOBt (56.0 mg, 0.4 mmol, 1.5 equiv.) was stirred in CH_2_Cl_2_ (2.7 mL) for 1 h at room temperature. A solution of compound **11** (150.0 mg, 0.2 mmol, 1.0 equiv.) and *N*-methylmorpholine (83.9 mg, 0.8 mmol, 3.0 equiv.) in CH_2_Cl_2_ (1.0 mL) was added to the reaction mixture. The reaction solution was stirred at room temperature for 15 h, quenched with saturated NH_4_Cl_(aq)_, dried over Na_2_SO_4_, and concentrated in vacuo. Purification of the crude residue by flash chromatography on silica gel was eluted with MeOH/DCM (5/95) to give compound **34** (176.5 mg, 77%). ^1^H NMR (400 MHz, CDCl_3_) δ 8.89 (s, 2H, CH-Py./DPA), 8.54–8.45 (m, 2H, CH-Py./DPA), 8.12 (d, *J* = 8.3 Hz, 2H, CH-Py./DPA), 7.67 (t, *J* = 7.9 Hz, 2H, CH-Py./DPA), 7.56 (t, *J* = 7.6 Hz, 2H, CH-Py./DPA), 7.49 (d, *J* = 7.8 Hz, 2H, CH-Py./DPA), 7.29 (s, 1H, CH-Ph./DPA), 7.23 (d, *J* = 10.8 Hz, 5H), 7.13 (dd, *J* = 7.2, 5.0 Hz, 2H, CH-Py./DPA), 6.87 (s, 1H), 6.72 (s, 2H, CH-Ph./DPA), 4.44 (d, *J* = 6.0 Hz, 2H), 4.27 (d, *J* = 6.0 Hz, 2H), 4.04 (s, 2H), 3.93 (t, *J* = 6.0 Hz, 2H), 3.84 (s, 2H), 3.77 (s, 4H), 3.71–3.64 (m, 6H), 3.62–3.50 (m, 10H), 3.31 (q, *J* = 6.6 Hz, 2H), 2.07 (s, 2H), 1.74 (d, *J* = 29.4 Hz, 10H), 1.55 (d, *J* = 8.5 Hz, 3H), 1.45 (d, *J* = 1.1 Hz, 9H), 1.21 (d, *J* = 14.8 Hz, 8H), 0.83 (t, *J* = 6.9 Hz, 6H, CH_3_-alkyl/DPA). HRMS (ESI): calc. for C_69_H_93_N_11_NaO_10_^+^: 1258.6999, found: 1258.6992.

#### (1*S*,2*R*,3*S*,5*S*,6*S*,16*E*,18*Z*,20*R*,21*S*)-11-Chloro-21-hydroxy-12,20-dimethoxy-2,5,9,16-tetramethyl-8,23-dioxo-4,24-dioxa-9,22-diazatetracyclo[19.3.1.1.^10,14^0^3,5^]hexacosa-10(26),11,13,16,18-pentaen-6-yl (2*S*)-2-[(3-{[3-({4-[18-(3,5-bis{[{[6-(Hexanoylamino)pyridin-2-yl]methyl}(pyridin-2-ylmethyl)amino]methyl}phenoxy)-3,13-dioxo-5,8,11-trioxa-2,14-diazaoctadec-1-yl]benzyl}amino)-3-oxopropyl]disulfanyl}propanoyl)(methyl)amino]propanoate (**35**)

To a solution of compound **34** (176.5 mg, 0.1 mmol) in CH_2_Cl_2_ (1.4 mL), TFA (1.4 mL) was added. The reaction mixture was stirred at room temperature overnight. After the reaction was completed, the excess amount of TFA was removed under vacuum to give the Boc-deprotected product. A mixture of compound **15** (148.6 mg, 0.2 mmol, 1.2 equiv.), EDCI (101.2 mg, 0.5 mmol, 3.0 equiv.), and HOBt (71.5 mg, 0.5 mmol, 3.0 equiv.) was stirred in CH_2_Cl_2_ (2.5 mL) for 1 h at room temperature. A solution of the Boc-deprotected product (181.8 mg, 0.1 mmol, 1.0 equiv.) and *N*-methylmorpholine (214.1 mg, 2.1 mmol, 12.0 equiv.) in CH_2_Cl_2_ (1.0 mL) was added to the reaction mixture and was stirred at room temperature overnight. The reaction mixture was washed with saturated NH_4_Cl_(aq)_, dried over Na_2_SO_4_, and concentrated in vacuo. Purification of the crude residue by flash chromatography on silica gel was eluted with MeOH/DCM (5/95) to give compound **35** (213.4 mg, 74% in two steps). ^1^H NMR (700 MHz, CDCl_3_) δ 8.87 (s, 2H, CH-Py./DPA), 8.44–8.38 (m, 2H, CH-Py./DPA), 8.05 (d, *J* = 8.2 Hz, 2H, CH-Py./DPA), 7.60 (t, *J* = 7.9 Hz, 2H, CH-Py./DPA), 7.50 (td, *J* = 7.6, 1.8 Hz, 2H, CH-Py./DPA), 7.43 (d, *J* = 7.8 Hz, 2H, CH-Py./DPA), 7.26 (t, *J* = 6.0 Hz, 1H), 7.23 (s, 1H, CH-Ph./DPA), 7.20 (s, 1H), 7.18 (d, *J* = 7.5 Hz, 2H, CH-Py./DPA), 7.12 (s, 4H), 7.09–7.06 (m, 2H), 6.96 (t, *J* = 5.5 Hz, 1H), 6.82 (t, *J* = 6.1 Hz, 1H), 6.76 (d, *J* = 1.8 Hz, 1H), 6.65 (d, *J* = 1.5 Hz, 2H), 6.61 (d, *J* = 11.2 Hz, 1H), 6.56 (d, *J* = 1.8 Hz, 1H), 6.35 (dd, *J* = 15.4, 11.2 Hz, 1H), 6.26–6.22 (m, 1H), 5.59 (dd, *J* = 15.3, 9.1 Hz, 1H), 5.27 (d, *J* = 7.0 Hz, 1H), 4.72 (dd, *J* = 12.1, 3.0 Hz, 1H), 4.38 (d, *J* = 6.0 Hz, 2H), 4.31 (d, *J* = 5.5 Hz, 2H), 4.25–4.19 (m, 1H), 3.98 (s, 3H, CH_3_-alkyl/DM1), 3.91 (s, 3H, CH_3_-alkyl/DM1), 3.86 (t, *J* = 6.2 Hz, 2H), 3.70 (s, 3H, CH_3_-alkyl/DM1), 3.62 (s, 3H, CH_3_-alkyl/DM1), 3.60–3.59 (m, 2H), 3.57 (d, *J* = 12.5 Hz, 1H), 3.55–3.54 (m, 3H), 3.51 (s, 3H, CH_3_-alkyl/DM1), 3.49 (dd, *J* = 4.1, 2.0 Hz, 1H), 3.41–3.39 (m, 2H), 3.23 (s, 3H, CH_3_-alkyl/DM1), 3.15 (s, 3H, CH_3_-alkyl/DM1), 3.13 (d, *J* = 6.8 Hz, 1H), 3.05 (d, *J* = 12.7 Hz, 1H), 2.94 (d, *J* = 9.7 Hz, 1H), 2.91–2.87 (m, 1H), 2.81–2.77 (m, 6H), 2.75 (dd, *J* = 8.1, 6.3 Hz, 1H), 2.62–2.53 (m, 2H), 2.53–2.50 (m, 2H), 2.11 (dd, *J* = 14.4, 3.0 Hz, 1H), 2.01 (d, *J* = 7.6 Hz, 3H), 1.89 (s, 7H), 1.69 (dq, *J* = 11.8, 6.5 Hz, 2H), 1.60–1.55 (m, 5H), 1.53–1.45 (m, 5H), 1.39 (td, *J* = 10.2, 6.3 Hz, 1H), 1.22 (dd, *J* = 6.7, 5.0 Hz, 6H), 1.19–1.13 (m, 5H), 1.12–1.08 (m, 4H), 0.76 (t, *J* = 7.2 Hz, 6H, CH_3_-alkyl/DPA), 0.73 (s, 3H, CH_3_-alkyl/DM1). ^13^C NMR (176 MHz, CDCl_3_) δ 172.3, 171.0, 170.8, 170.8, 170.0, 169.9, 168.9, 159.7, 158.9, 157.9, 156.1, 152.4, 151.5, 148.9 (CH), 142.2, 141.1, 140.2, 139.4, 139.1 (CH), 137.8, 137.5, 136.7 (CH), 133.3 (CH), 128.0 (CH), 127.9 (CH), 125.4 (CH), 123.0, 122.3 (CH), 122.2 (CH), 119.0 (CH), 118.9 (CH), 113.9 (CH), 113.3 (CH), 112.5 (CH), 88.8 (CH), 80.9, 78.3 (CH), 74.2 (CH), 70.9 (CH_2_), 70.8 (CH_2_), 70.5 (CH_2_), 70.2 (CH_2_), 70.1 (CH_2_), 70.0 (CH_2_), 67.4 (CH_2_), 67.2 (CH), 60.2 (CH_2_), 60.1 (CH_2_), 59.5 (CH_2_), 58.2 (CH_2_), 56.7, 56.7 (CH_3_), 52.8 (CH), 46.7 (CH_2_), 43.5 (CH_2_), 42.5 (CH_2_), 39.0 (CH), 38.7 (CH_2_), 37.5 (CH_2_), 36.5 (CH_2_), 35.8 (CH_2_), 35.7 (CH_3_), 33.6 (CH_2_), 33.5 (CH_2_), 33.2 (CH_2_), 32.5 (CH_2_), 31.4 (CH_2_), 31.1 (CH_3_), 26.9 (CH_2_), 26.4 (CH_2_), 25.0 (CH_2_), 22.4 (CH_2_), 15.6 (CH_3_), 14.7 (CH_3_), 14.0 (CH_3_), 13.5 (CH_3_), 12.3 (CH_3_). HRMS (ESI): calc. for C_102_H_134_ClN_14_O_19_S_2_^-^: 1957.9085, found: 1957.8919.

#### (1*S*,2*R*,3*S*,5*S*,6*S*,16*E*,18*Z*,20*R*,21*S*)-11-Chloro-21-hydroxy-12,20-dimethoxy-2,5,9,16-tetramethyl-8,23-dioxo-4,24-dioxa-9,22-diazatetracyclo[19.3.1.1.^10,14^0^3,5^]hexacosa-10(26),11,13,16,18-pentaen-6-yl (2*S*)-2-[(3-{[3-({4-[18-(3,5-bis{[{[6-(Hexanoylamino)pyridin-2-yl]methyl}(pyridin-2-ylmethyl)amino]methyl}phenoxy)-3,13-dioxo-5,8,11-trioxa-2,14-diazaoctadec-1-yl]benzyl}amino)-3-oxopropyl]disulfanyl}propanoyl)(methyl)amino]propanoate·2[Zn(NO_3_)_2_] (**36**)

^1^H NMR (700 MHz, DMSO-*d*_6_) δ 11.91 (s, 1H), 8.43 (d, *J* = 5.2 Hz, 2H, CH-Py./DPA), 8.39 (t, *J* = 6.0 Hz, 1H), 8.23 (t, *J* = 6.2 Hz, 1H), 8.05–7.99 (m, 4H, CH-Py./DPA), 7.79 (t, *J* = 6.0 Hz, 1H), 7.58 (t, *J* = 6.4 Hz, 2H), 7.51 (t, *J* = 7.3 Hz, 2H, CH-Py./DPA), 7.31 (d, *J* = 6.7 Hz, 2H, CH-Py./DPA), 7.22–7.15 (m, 10H), 6.89 (s, 1H, CH-Ph./DPA), 6.58 (t, *J* = 10.0 Hz, 1H), 6.55–6.53 (m, 1H), 5.93 (d, *J* = 1.6 Hz, 1H), 5.56 (dd, *J* = 14.5, 9.1 Hz, 1H), 5.31 (q, *J* = 6.8 Hz, 1H), 4.52 (dd, *J* = 12.0, 2.8 Hz, 1H), 4.43 (dd, *J* = 16.1, 10.8 Hz, 3H), 4.26 (d, *J* = 6.2 Hz, 2H), 4.20 (d, *J* = 6.0 Hz, 2H), 4.14 (q, *J* = 4.6 Hz, 3H), 4.08–4.04 (m, 1H), 4.00 (d, *J* = 14.0 Hz, 2H), 3.96–3.85 (m, 8H), 3.78 (dd, *J* = 15.6, 3.3 Hz, 2H), 3.73–3.68 (m, 2H), 3.61–3.57 (m, 5H), 3.56 (s, 3H), 3.48 (t, *J* = 11.7 Hz, 2H), 3.25 (s, 3H, CH_3_-alkyl/DM1), 3.22 (d, *J* = 6.6 Hz, 1H), 3.19 (d, *J* = 12.4 Hz, 1H), 3.11 (s, 3H, CH_3_-alkyl/DM1), 2.92 (dd, *J* = 13.4, 6.5 Hz, 1H), 2.88–2.81 (m, 2H), 2.78 (d, *J* = 9.7 Hz, 2H), 2.71 (d, *J* = 8.5 Hz, 6H), 2.61–2.56 (m, 1H), 2.45–2.37 (m, 2H), 2.03 (dd, *J* = 14.5, 2.9 Hz, 1H), 1.85–1.79 (m, 4H), 1.79–1.75 (m, 2H), 1.65 (t, *J* = 7.5 Hz, 2H), 1.59 (s, 3H, CH_3_-alkyl/DM1), 1.47–1.39 (m, 10H), 1.28–1.21 (m, 5H), 1.17 (d, *J* = 6.8 Hz, 3H, CH_3_-alkyl/DM1), 1.11 (d, *J* = 6.3 Hz, 3H, CH_3_-alkyl/DM1), 0.96–0.91 (m, 6H, CH_3_-alkyl/DPA), 0.85 (t, *J* = 6.9 Hz, 3H), 0.78 (s, 3H, CH_3_-alkyl/DM1). ^13^C NMR (176 MHz, DMSO-*d*_6_) δ 178.5, 170.6, 170.3, 169.9, 169.3, 169.2, 168.2, 159.0, 155.3, 154.1, 152.3, 151.3, 147.1 (CH), 142.6 (CH), 141.3, 141.3, 140.5 (CH), 138.4, 138.0, 137.9, 133.8, 132.6 (CH), 128.5 (CH), 127.3 (CH), 127.2, 127.1, 125.2 (CH), 124.8 (CH), 124.4 (CH), 121.7 (CH), 120.7 (CH), 117.8 (CH), 117.1, 114.6, 114.0 (CH), 88.2 (CH), 80.0, 77.8 (CH), 73.2 (CH), 70.3 (CH_2_), 70.2 (CH_2_), 70.1 (CH_2_), 70.0 (CH_2_), 70.0 (CH_2_), 69.6 (CH_2_), 69.5 (CH_2_), 67.5 (CH_2_), 66.8 (CH), 60.1 (CH_2_), 58.7, 57.0 (CH_2_), 56.9 (CH_2_), 56.6 (CH), 56.2 (CH), 55.8 (CH_2_), 55.7, 51.7, 45.5 (CH_2_), 41.9 (CH_2_), 41.4 (CH_2_), 37.8 (CH), 37.7, 36.9 (CH_2_), 36.4 (CH_2_), 35.3 (CH_3_), 34.8 (CH_2_), 33.4 (CH_2_), 33.2 (CH_2_), 33.0 (CH_2_), 32.0 (CH_2_), 31.0 (CH_2_), 30.8 (CH_3_), 29.8, 26.2 (CH_2_), 26.1 (CH_2_), 24.6 (CH_2_), 22.1 (CH_2_), 21.9, 15.1 (CH_3_), 14.5 (CH_3_), 13.9 (CH_3_), 13.1 (CH_3_), 11.4 (CH_3_). HRMS (ESI): calc. for C_102_H_134_ClN_18_O_31_S_2_Zn_2_^-^: 2333.7181, found: 2333.7784.

#### *N*,*N*’-[(5-{4-[(Biphenyl-4-ylmethyl)amino]butoxy}benzene-1,3-diyl)bis{methanediyl[(pyridin-2-ylmethyl)imino]methanediylpyridine-6,2-diyl}]dihexanamide (**37a**)

To a solution of compound **11** (200 mg, 0.246 mmol) in MeOH (3 mL) at room temperature, biphenyl-4-carboxaldehyde (90 mg, 0.491 mmol) was added, and then the reaction was slowly warmed to 70 °C and stirred for 24 h. The reaction mixture was cooled down to 0 °C, and then sodium borohydride (37 mg, 0.983 mmol) was slowly added. The reaction mixture was slowly warmed to room temperature and stirred for 2 h. After the reaction was completed, the reaction mixture was quenched with saturated NH_4_Cl_(aq)_ at ice-bath temperature and then adjusted to pH 6–7. Organic volatiles were evaporated, and then the residue was partitioned into H_2_O and CH_2_Cl_2_. The aqueous phase was extracted with CH_2_Cl_2_ (5 mL × 3), and the combined organic layers were dried over Na_2_SO_4_, filtered, and concentrated in vacuo. The residue was purified by flash chromatography (5% MeOH in CH_2_Cl_2_) to yield the compound **37a** (165 mg, 68%). ^1^H NMR (400 MHz, CDCl_3_) δ 8.87 (s, 2H, CH-Py./DPA), 8.51–8.47 (m, 2H, CH-Py./DPA), 8.12 (d, *J* = 8.1 Hz, 2H, CH-Py./DPA), 7.67 (td, *J* = 7.9, 2.0 Hz, 2H, CH-Py./DPA), 7.59–7.28 (m, 16H), 7.15–7.10 (m, 2H, CH-Py./DPA), 6.73 (t, *J* = 1.7 Hz, 2H, CH-Ph./DPA), 3.98–3.92 (m, 2H), 3.85 (d, *J* = 1.9 Hz, 2H), 3.78 (d, *J* = 1.9 Hz, 4H), 3.69 (d, *J* = 1.9 Hz, 4H), 3.58 (s, 4H), 2.78–2.71 (m, 2H), 2.10–2.02 (m, 4H), 1.87–1.78 (m, 4H), 1.58–1.51 (m, 4H), 1.27–1.11 (m, 9H), 0.83 (td, *J* = 7.0, 1.9 Hz, 6H, CH_3_-alkyl/DPA). ^13^C NMR (151 MHz, CDCl_3_) δ 172.2, 159.8, 159.1, 158.0, 151.5, 149.0 (CH), 141.1, 140.2, 140.1, 139.6, 139.1 (CH), 136.6 (CH), 130.4, 129.2, 128.9, 128.7 (CH), 128.5 (CH), 128.0, 127.8, 127.5, 127.3, 127.3 (CH), 127.2 (CH), 126.0, 123.0 (CH), 122.2 (CH), 122.1 (CH), 119.1 (CH), 113.9 (CH), 112.5 (CH), 67.8 (CH_3_), 60.3 (CH_3_), 59.5 (CH_3_), 58.2 (CH_3_), 53.8 (CH_3_), 49.2 (CH_3_), 37.5 (CH_3_), 31.4 (CH_3_), 27.3 (CH_3_), 26.8 (CH_3_), 25.0 (CH_3_), 22.4 (CH_3_), 14.0 (CH_2_). HRMS (ESI): calc. for C_61_H_74_N_9_O_3_^+^: 980.5909, found: 980.5898.

#### *tert*-Butyl-{4-[14-(biphenyl-4-ylmethyl)-18-(3,5-bis{[{[6-(hexanoylamino)pyridin-2-yl]methyl}(pyridin-2-ylmethyl)amino]methyl}phenoxy)-3,13-dioxo-5,8,11-trioxa-2,14-diazaoctadec-1-yl]benzyl}carbamate (**38a**)

To a solution of compound **23** (66 mg, 0.149 mmol) in CH_2_Cl_2_ (1.5 mL) at room temperature, *O*-(benzotriazol-1-yl)-*N*,*N*,*N*’,*N*’-tetramethyluronium hexafluorophosphate (HBTU, 56 mg, 0.149 mmol) was added followed by hydroxybenzotriazole (HOBt, 20 mg, 0.146 mmol). After the reaction mixture was stirred for 1 h, compound **37a** (73 mg, 0.074 mmol) and *N*-methylmorpholine (NMM, 0.02 mL, 0.149 mmol) were added subsequently, and then the resultant mixture was stirred for 17 h. After the reaction was completed, the resultant mixture was quenched with saturated NH_4_Cl_(aq)_ and extracted with CH_2_Cl_2_ (5 mL × 2). The combined organic layers were dried over Na_2_SO_4_, filtered, and concentrated in vacuo. The residue was purified by flash chromatography (5% MeOH in CH_2_Cl_2_) to yield the compound **38a** (73 mg, 70%). ^1^H NMR (600 MHz, DMSO-*d*_6_) δ 10.36 (s, 2H, NH-amide/DPA), 8.49 (ddd, *J* = 4.9, 1.8, 0.9 Hz, 2H, CH-Py./DPA), 8.09 (s, 2H), 7.96 (d, *J* = 8.2 Hz, 3H), 7.78–7.66 (m, 4H, CH-Py./DPA), 7.66–7.57 (m, 4H, CH-Py./DPA), 7.55–7.48 (m, 2H, CH-Py./DPA), 7.44 (t, *J* = 7.7 Hz, 2H), 7.36 (dd, *J* = 7.7, 5.5 Hz, 3H), 7.29 (d, *J* = 7.5 Hz, 2H, CH-Py./DPA), 7.28–7.21 (m, 2H), 7.08 (s, 1H, CH-Ph./DPA), 6.78 (d, *J* = 1.3 Hz, 3H), 5.32 (t, *J* = 4.5 Hz, 1H), 4.48 (s, 2H), 4.24 (p, *J* = 5.3 Hz, 2H), 3.70 (s, 3H), 3.67–3.54 (m, 6H), 2.35 (t, *J* = 7.4 Hz, 3H), 2.15–2.08 (m, 2H), 2.05–1.94 (m, 6H), 1.70 (dd, *J* = 12.1, 5.8 Hz, 4H), 1.55 (p, *J* = 8.2 Hz, 6H), 1.48–1.38 (m, 3H), 1.37 (q, *J* = 5.5 Hz, 2H), 1.33–1.18 (m, 18H), 1.07 (t, *J* = 7.1 Hz, 3H), 0.91 (t, *J* = 7.5 Hz, 3H), 0.89–0.78 (m, 9H). ^13^C NMR (151 MHz, CDCl_3_) δ 172.2, 170.1, 159.8, 159.7, 158.0, 157.9, 156.1, 151.6, 151.5, 149.0, 149.0 (CH), 140.3, 140.2, 139.1 (CH), 138.3, 137.5, 136.6 (CH), 136.4, 129.0 (CH), 128.9 (CH), 128.7 (CH), 128.1 (CH), 128.0 (CH), 127.8 (CH), 127.7 (CH), 127.7 (CH), 127.5 (CH), 127.4 (CH), 127.1 (CH), 127.0 (CH), 125.9 (CH), 123.0 (CH), 122.3 (CH), 122.2 (CH), 119.1, 113.9 (CH), 112.5 (CH), 112.5 (CH), 79.6, 71.1 (CH_3_), 70.7 (CH_3_), 70.6 (CH_3_), 70.61, 70.5 (CH_3_), 70.3 (CH_3_), 70.2 (CH_3_), 70.0 (CH_3_), 67.5 (CH_3_), 67.3 (CH_3_), 60.3 (CH_3_), 59.5 (CH_3_), 58.2 (CH_3_), 50.0 (CH_3_), 48.0 (CH_3_), 46.0 (CH_3_), 45.9 (CH_3_), 44.4 (CH_3_), 42.6 (CH_3_), 37.5 (CH), 31.4 (CH), 28.6 (CH_2_), 26.9, 26.7, 25.4, 25.0, 24.2 (CH), 22.4, 14.0 (CH_2_), 1.2 (CH_2_). HRMS (ESI): calc. for C_82_H_104_N_11_O_10_^+^: 1402.7962, found: 1402.8004.

#### (1*S*,2*R*,3*S*,5*S*,6*S*,16*E*,18*Z*,20*R*,21*S*)-11-Chloro-21-hydroxy-12,20-dimethoxy-2,5,9,16-tetramethyl-8,23-dioxo-4,24-dioxa-9,22-diazatetracyclo[19.3.1.1.^10,14^0^3,5^]hexacosa-10(26),11,13,16,18-pentaen-6-yl (2*S*)-2-[(3-{[3-({4-[14-(Biphenyl-4-ylmethyl)-18-(3,5-bis{[{[6-(hexanoylamino)pyridin-2-yl]methyl}(pyridin-2-ylmethyl)amino]methyl}phenoxy)-3,13-dioxo-5,8,11-trioxa-2,14-diazaoctadec-1-yl]benzyl}amino)-3-oxopropyl]disulfanyl}propanoyl)(methyl)amino]propanoate (**39a**)

To a solution of compound **38a** (432 mg, 0.31 mmol) in CH_2_Cl_2_ (5 mL) at room temperature, TFA (5 mL) was added, and then the reaction was stirred for 2 h. The excess TFA was removed under reduced pressure to give the Boc-deprotected product (420 mg, 0.31 mmol), which was used for the next reaction without further purification. The process was repeated on another batch and to a solution of compound **15** (194 mg, 0.230 mmol) in CH_2_Cl_2_ (3 mL) at room temperature, 1-(3-dimethylaminopropyl)-3-ethylcarbodiimide hydrochloride (EDCI, 44 mg, 0.230 mmol) and HOBt (31 mg, 0.230 mmol) were added. After the reaction mixture was stirred for 1 h, the Boc-deprotected product (200 mg, 0.154 mmol) and NMM (1 mL, 0.921 mmol) were added consecutively, and then the reaction was stirred for 18 h. After the reaction was completed, the resultant mixture was quenched with saturated NH_4_Cl_(aq)_ and extracted with CH_2_Cl_2_ (10 mL × 2). The combined organic layers were dried over Na_2_SO_4_, filtered, and concentrated in vacuo. The residue was purified by flash chromatography (7% MeOH in CH_2_Cl_2_) to yield the compound **39a** (146 mg, 45% in two steps). ^1^H NMR (600 MHz, DMSO-*d*_6_) δ 10.34 (d, *J* = 2.2 Hz, 2H, NH-amide/DPA), 8.47 (dd, *J* = 4.8, 1.6 Hz, 2H, CH-Py./DPA), 8.37 (t, *J* = 5.9 Hz, 1H), 8.19 (q, *J* = 7.0 Hz, 1H), 7.95 (d, *J* = 8.3 Hz, 2H), 7.75–7.65 (m, 4H, CH-Py./DPA), 7.63–7.59 (m, 2H, CH-Py./DPA), 7.59–7.56 (m, 1H), 7.54 (dd, *J* = 8.0, 6.2 Hz, 1H), 7.51 (dd, *J* = 8.2, 2.9 Hz, 2H, CH-Py./DPA), 7.41 (dt, *J* = 10.6, 7.6 Hz, 2H), 7.33 (dt, *J* = 9.4, 7.4 Hz, 1H), 7.30–7.25 (m, 3H), 7.25–7.20 (m, 2H), 7.16 (qd, *J* = 8.3, 3.9 Hz, 3H), 7.13 (d, *J* = 1.8 Hz, 1H), 7.06 (d, *J* = 12.4 Hz, 1H), 6.91 (s, 1H, CH-Py./DPA), 6.76 (s, 2H, CH-Ph./DPA), 6.63–6.55 (m, 1H), 6.55–6.50 (m, 1H), 5.94 (d, *J* = 1.4 Hz, 1H), 5.56 (dd, *J* = 14.8, 9.0 Hz, 1H), 5.31 (q, *J* = 6.7 Hz, 1H), 4.60–4.45 (m, 3H), 4.29–4.14 (m, 6H), 4.06 (t, *J* = 12.1 Hz, 1H), 3.91 (s, 1H), 3.89 (s, 3H, CH_3_-alkyl/DM1), 3.69 (s, 4H), 3.61 (s, 4H), 3.59–3.43 (m, 14H), 3.24 (s, 3H, CH_3_-alkyl/DM1), 3.17 (d, *J* = 12.5 Hz, 1H), 3.12 (s, 3H, CH_3_-alkyl/DM1), 2.94–2.76 (m, 5H), 2.70 (d, *J* = 11.4 Hz, 5H), 2.44–2.37 (m, 2H), 2.34 (t, *J* = 7.4 Hz, 4H), 2.21–2.15 (m, 1H), 2.12 (s, 1H), 2.04 (dd, *J* = 15.5, 3.7 Hz, 1H), 1.65 (s, 4H), 1.58 (s, 3H, CH_3_-alkyl/DM1), 1.54 (q, *J* = 7.4 Hz, 4H), 1.50–1.41 (m, 3H), 1.32–1.19 (m, 14H), 1.16 (d, *J* = 6.8 Hz, 3H, CH_3_-alkyl/DM1), 1.12 (d, *J* = 6.4 Hz, 3H, CH_3_-alkyl/DM1), 0.84 (t, *J* = 7.1 Hz, 7H), 0.77 (s, 3H, CH_3_-alkyl/DM1). ^13^C NMR (151 MHz, CDCl_3_) δ 172.3, 171.0, 170.7, 170.2, 169.6, 168.9, 159.7, 159.0, 157.9, 156.1, 152.4, 151.6, 151.5, 148.9 (CH), 142.3, 141.1, 140.3, 140.2, 139.4, 139.1 (CH), 137.6, 136.6 (CH), 133.3 (CH),131.2 (CH), 129.1 (CH), 129.0 (CH), 128.6 (CH), 128.4 (CH), 128.2 (CH), 128.2 (CH), 128.0 (CH), 127.9 (CH), 127.7 (CH), 127.6 (CH), 127.5 (CH), 127.1 (CH), 127.0 (CH), 125.9 (CH), 125.4 (CH), 123.0 (CH), 122.3 (CH), 122.2 (CH), 119.0 (CH), 118.9, 113.9 (CH), 113.3 (CH), 112.5 (CH), 88.8 (CH), 80.9, 78.3 (CH), 74.2 (CH), 71.2 (CH_3_), 70.5 (CH_3_), 70.4, 70.4, 70.3 (CH_3_), 67.5 (CH_3_), 67.2 (CH_2_), 60.2, 60.1 (CH_3_), 59.5 (CH_3_), 58.2 (CH_3_), 56.7 (CH_2_), 56.7, 52.9 (CH_2_), 50.0 (CH_3_), 48.0 (CH_3_), 46.7 (CH_3_), 46.1 (CH_3_), 45.9 (CH_3_), 43.4 (CH_3_), 42.6 (CH_3_), 39.0 (CH_2_), 37.5 (CH_3_), 36.4 (CH_3_), 35.8, 35.7 (CH_2_), 33.6, 33.3 (CH_3_), 32.6 (CH_3_), 31.4 (CH_3_), 31.1, 29.8 (CH_3_), 26.9 (CH_3_), 26.7 (CH_3_), 25.4 (CH_3_), 25.0 (CH_3_), 24.3 (CH_3_), 22.8, 22.4 (CH_3_), 20.6 (CH_2_), 19.2 (CH_2_), 15.6 (CH_2_), 14.7 (CH_2_), 14.0 (CH_2_), 13.5 (CH_2_), 12.3 (CH_2_). HRMS (ESI): calc. for C_115_H_145_ClN_14_NaO_19_S_2_^+^: 2147.9833, found: 2147.9836.

#### (1*S*,2*R*,3*S*,5*S*,6*S*,16*E*,18*Z*,20*R*,21*S*)-11-Chloro-21-hydroxy-12,20-dimethoxy-2,5,9,16-tetramethyl-8,23-dioxo-4,24-dioxa-9,22-diazatetracyclo[19.3.1.1.^10,14^0^3,5^]hexacosa-10(26),11,13,16,18-pentaen-6-yl (2*S*)-2-[(3-{[3-({4-[14-(Biphenyl-4-ylmethyl)-18-(3,5-bis{[{[6-(hexanoylamino)pyridin-2-yl]methyl}(pyridin-2-ylmethyl)amino]methyl}phenoxy)-3,13-dioxo-5,8,11-trioxa-2,14-diazaoctadec-1-yl]benzyl}amino)-3-oxopropyl]disulfanyl}propanoyl)(methyl)amino]propanoate·2[Zn(NO_3_)_2_] (**40a**)

To a solution of zinc nitrate hexahydrate (41 mg, 0.138 mmol) in MeOH (10 mL), compound **39a** (146 mg, 0.069 mmol) in CH_2_Cl_2_ (10 mL) was added dropwise. The reaction mixture was sonicated at room temperature till all the solid was dissolved. The resulting mixture was removed and kept under reduced pressure until the weight was not changed. Compound **40a** was obtained as a white powder (172 mg, quant). ^1^H NMR (600 MHz, DMSO-*d*_6_) δ 11.90 (s, 2H, NH-amide/DPA), 8.42 (s, 2H, CH-Py./DPA), 8.39 (s, 1H), 8.20 (t, *J* = 6.2 Hz, 1H), 8.02 (s, 4H), 7.67 (d, *J* = 8.0 Hz, 1H), 7.64 (d, *J* = 7.5 Hz, 1H), 7.62–7.56 (m, 4H, CH-Py./DPA), 7.49 (dt, *J* = 13.0, 7.0 Hz, 2H, CH-Py./DPA), 7.44 (q, *J* = 8.2 Hz, 2H), 7.37–7.28 (m, 6H, CH-Py./DPA), 7.22–7.12 (m, 10H), 6.91 (s, 1H, CH-Ph./DPA), 6.58 (t, *J* = 9.5 Hz, 2H), 6.55–6.52 (m, 2H), 5.94 (s, 1H), 5.55 (dd, *J* = 14.4, 9.0 Hz, 1H), 5.30 (q, *J* = 6.8 Hz, 1H), 4.59 (t, *J* = 13.2 Hz, 2H), 4.52 (dd, *J* = 12.1, 2.8 Hz, 1H), 4.42 (t, *J* = 13.5 Hz, 3H), 4.31 (s, 1H), 4.24 (d, *J* = 5.7 Hz, 3H), 4.19 (d, *J* = 5.8 Hz, 3H), 4.12 (s, 3H), 4.06 (t, *J* = 11.4 Hz, 2H), 3.99 (d, *J* = 15.1 Hz, 2H), 3.90 (s, 3H, CH_3_-alkyl/DM1), 3.76 (d, *J* = 17.2 Hz, 2H), 3.72–3.65 (m, 2H), 3.62–3.45 (m, 12H), 3.24 (s, 3H), 3.18 (d, *J* = 12.4 Hz, 1H), 3.11 (s, 3H, CH_3_-alkyl/DM1), 2.93–2.87 (m, 1H), 2.83 (s, 1H), 2.78 (d, *J* = 9.8 Hz, 2H), 2.71 (s, 3H), 2.40 (q, *J* = 7.7 Hz, 2H), 2.03 (d, *J* = 14.2 Hz, 1H), 1.82 (s, 4H), 1.78–1.68 (m, 5H), 1.58 (s, 3H, CH_3_-alkyl/DM1), 1.48–1.36 (m, 11H), 1.24 (q, *J* = 6.9 Hz, 5H), 1.16 (d, *J* = 6.7 Hz, 3H, CH_3_-alkyl/DM1), 1.11 (d, *J* = 6.3 Hz, 3H, CH_3_-alkyl/DM1), 0.98–0.90 (m, 6H), 0.87–0.81 (m, 4H), 0.77 (s, 3H, CH_3_-alkyl/DM1). ^13^C NMR (176 MHz, DMSO-*d*_6_) δ 178.5, 170.5, 170.3, 169.8, 169.2, 168.9, 168.2, 158.9, 155.3, 154.0, 152.3, 151.2, 147.1 (CH), 142.6 (CH), 141.3, 141.2, 140.5 (CH), 139.8, 139.7, 139.2, 138.4, 138.0, 137.9, 136.7, 133.8, 132.6 (CH), 129.0 (CH), 128.9, 128.5 (CH), 128.1 (CH), 127.5 (CH), 127.4 (CH), 127.2 (CH), 127.0 (CH), 126.7 (CH), 126.6, 126.5 (CH), 125.2 (CH), 124.8 (CH), 124.3 (CH), 121.6 (CH), 120.6 (CH), 117.8 (CH), 117.1, 114.6, 113.9 (CH), 88.2 (CH), 80.0, 77.7 (CH), 73.2 (CH), 70.3 (CH_2_), 70.3 (CH_2_), 70.0 (CH_2_), 69.9 (CH_2_), 69.7 (CH_2_), 69.6 (CH_2_), 69.5 (CH_2_), 69.3 (CH_2_), 68.9 (CH_2_), 67.5 (CH_2_), 66.8 (CH), 60.0, 58.7 (CH_2_), 56.9 (CH_2_), 56.5 (CH_3_), 56.1 (CH_3_), 55.7, 55.6 (CH_2_), 51.7 (CH), 49.0 (CH_2_), 47.2 (CH_2_), 45.8 (CH_2_), 45.6 (CH_2_), 44.8 (CH_2_), 41.9 (CH_2_), 41.6 (CH_2_), 37.6 (CH), 36.9 (CH_2_), 36.3 (CH_2_), 35.2 (CH_3_), 34.7 (CH_2_), 33.4 (CH_2_), 33.2 (CH_2_), 32.9 (CH_2_), 31.9 (CH_2_), 31.0 (CH_2_), 30.8 (CH_2_), 29.7 (CH_3_), 26.2 (CH_2_), 26.0 (CH_2_), 24.8 (CH_2_), 24.6 (CH_2_), 23.6 (CH_2_), 22.0 (CH_2_), 21.9 (CH_2_), 15.1 (CH_3_), 14.4 (CH_3_), 14.0 (CH_3_), 13.9 (CH_3_), 13.1 (CH_3_), 11.4 (CH_3_). HRMS (ESI): *m/z* calc. for C_115_H_144_ClN_18_O_31_S_2_Zn_2_^–^: 2503.7994, found: 2503.7896.

#### (1*S*,2*R*,3*S*,5*S*,6*S*,16*E*,18*Z*,20*R*,21*S*)-11-Chloro-21-hydroxy-12,20-dimethoxy-2,5,9,16-tetramethyl-8,23-dioxo-4,24-dioxa-9,22-diazatetracyclo[19.3.1.1.^10,14^0^3,5^]hexacosa-10(26),11,13,16,18-pentaen-6-yl (2*S*)-2-[(3-{[5-({4-[14-(Biphenyl-4-ylmethyl)-18-(3,5-bis{[{[6-(hexanoylamino)pyridin-2-yl]methyl}(pyridin-2-ylmethyl)amino]methyl}phenoxy)-3,13-dioxo-5,8,11-trioxa-2,14-diazaoctadec-1-yl]benzyl}amino)-2-methyl-5-oxopentan-2-yl]disulfanyl}propanoyl)(methyl)amino]propanoate (**39b**)

To a solution of compound **19** (85 mg, 0.096 mmol) in CH_2_Cl_2_ (1 mL) at room temperature, EDCI (27 mg, 0.143 mmol) was added followed by HOBt (19 mg, 0.143 mmol). After the reaction was stirred at room temperature for 1 h, Boc-deprotected product **38a** (83 mg, 0.064 mmol) and NMM (0.04 mL, 0.382 mmol) were added consecutively. After the reaction was completed, the resultant mixture was quenched with saturated NH_4_Cl_(aq)_ and extracted with CH_2_Cl_2_ (10 mL × 2). The combined organic layers were dried over Na_2_SO_4_, filtered, and concentrated in vacuo. The residue was purified by flash chromatography (5% MeOH in CH_2_Cl_2_) to yield the compound **39b** (50 mg, 36% in two steps). ^1^H NMR (600 MHz, CDCl_3_) δ 8.94 (d, *J* = 24.6 Hz, 2H, CH-Py./DPA), 8.50–8.45 (m, 2H, CH-Py./DPA), 8.12 (dd, *J* = 8.4, 3.9 Hz, 2H, CH-Py./DPA), 7.67 (t, *J* = 7.9 Hz, 2H, CH-Py./DPA), 7.62 (t, *J* = 6.1 Hz, 1H), 7.57–7.52 (m, 4H, CH-Py./DPA), 7.49 (dd, *J* = 11.8, 8.0 Hz, 3H), 7.42 (dt, *J* = 15.0, 7.6 Hz, 2H), 7.38–7.27 (m, 3H), 7.26–7.18 (m, 7H), 7.15–7.11 (m, 2H, CH-Py./DPA), 6.84–6.80 (m, 2H, CH-Ph./DPA), 6.71–6.60 (m, 4H), 6.42–6.32 (m, 2H), 5.58 (dd, *J* = 15.4, 9.1 Hz, 1H), 5.32 (q, *J* = 6.8 Hz, 1H), 4.74 (dt, *J* = 12.0, 2.5 Hz, 1H), 4.58 (d, *J* = 59.5 Hz, 2H), 4.51–4.43 (m, 2H), 4.39 (dd, *J* = 14.9, 5.9 Hz, 1H), 4.33–4.25 (m, 2H), 4.17 (d, *J* = 13.8 Hz, 2H), 4.10 (s, 1H), 4.02 (d, *J* = 13.2 Hz, 2H), 3.97 (s, 3H), 3.91 (dt, *J* = 10.3, 5.2 Hz, 2H), 3.77 (s, 4H), 3.69 (d, *J* = 1.8 Hz, 4H), 3.65 (d, *J* = 4.6 Hz, 1H), 3.64–3.53 (m, 12H), 3.44 (dd, *J* = 10.5, 6.2 Hz, 2H), 3.28 (d, *J* = 7.2 Hz, 2H), 3.24 (d, *J* = 7.8 Hz, 1H), 3.22 (d, *J* = 2.1 Hz, 3H), 3.08 (d, *J* = 12.7 Hz, 1H), 3.02 (d, *J* = 9.7 Hz, 1H), 2.97–2.90 (m, 1H), 2.83 (d, *J* = 4.8 Hz, 3H), 2.82–2.72 (m, 2H), 2.61 (dt, *J* = 14.4, 11.1 Hz, 2H), 2.26–2.15 (m, 3H), 2.05 (q, *J* = 7.5 Hz, 4H), 1.99–1.86 (m, 8H), 1.78–1.69 (m, 4H), 1.56–1.50 (m, 5H), 1.45 (td, *J* = 10.2, 6.4 Hz, 1H), 1.28 (d, *J* = 6.3 Hz, 3H), 1.22–1.14 (m, 16H), 0.82 (td, *J* = 7.2, 1.2 Hz, 6H, CH_3_-alkyl/DPA), 0.79 (s, 3H). ^13^C NMR (151 MHz, CDCl_3_) δ 172.6, 172.2, 171.3, 170.7, 170.2, 168.9, 159.7, 158.0, 157.9, 156.1, 152.4, 151.6, 151.5, 149.0, 148.9 (CH), 142.3, 141.1, 140.3, 140.2, 139.1 (CH), 137.8, 137.7, 136.6 (CH), 133.2 (CH), 129.0 (CH), 128.6 (CH), 128.1 (CH), 128.0 (CH), 127.7 (CH), 127.5 (CH), 127.1 (CH), 127.0 (CH), 125.3 (CH), 123.0 (CH), 122.3 (CH), 122.2 (CH), 119.1 (CH), 119.0, 118.9, 113.9 (CH), 113.3 (CH), 112.5 (CH), 88.9 (CH), 80.8, 78.5 (CH), 74.2 (CH), 71.1 (CH_3_), 70.6 (CH_3_), 70.5 (CH_3_), 70.4 (CH_3_), 70.3 (CH_3_), 70.2 (CH_3_), 67.5 (CH), 60.2 (CH_3_), 60.0, 59.5 (CH_3_), 58.1 (CH_3_), 56.7 (CH_2_), 52.6 (CH_2_), 51.1 (CH_3_), 50.0 (CH_3_), 48.0 (CH_3_), 46.6 (CH_3_), 45.9 (CH_3_), 43.29 (CH_3_), 42.5 (CH_3_), 39.0 (CH_2_), 37.5 (CH_3_), 37.1 (CH_3_), 36.5 (CH_3_), 35.7 (CH_2_), 34.6 (CH_3_), 34.0 (CH_3_), 32.6 (CH_3_), 30.9 (CH_2_), 27.9 (CH_2_), 27.6 (CH_2_), 26.9 (CH_3_), 26.7 (CH_3_), 25.0 (CH_3_), 24.2 (CH_3_), 22.4 (CH_3_), 15.6 (CH_3_), 14.7 (CH_3_), 14.0 (CH_3_), 13.3 (CH_3_), 12.2 (CH_3_). HRMS (ESI): calc. for C_118_H_151_ClN_14_NaO_19_S_2_^+^: 2190.0302, found: 2190.0301.

#### (1*S*,2*R*,3*S*,5*S*,6*S*,16*E*,18*Z*,20*R*,21*S*)-11-Chloro-21-hydroxy-12,20-dimethoxy-2,5,9,16-tetramethyl-8,23-dioxo-4,24-dioxa-9,22-diazatetracyclo[19.3.1.1.^10,14^0^3,5^]hexacosa-10(26),11,13,16,18-pentaen-6-yl (2*S*)-2-[(3-{[5-({4-[14-(Biphenyl-4-ylmethyl)-18-(3,5-bis{[{[6-(hexanoylamino)pyridin-2-yl]methyl}(pyridin-2-ylmethyl)amino]methyl}phenoxy)-3,13-dioxo-5,8,11-trioxa-2,14-diazaoctadec-1-yl]benzyl}amino)-2-methyl-5-oxopentan-2-yl]disulfanyl}propanoyl)(methyl)amino]propanoate·2[Zn(NO_3_)_2_] (**40b**)

To a solution of zinc nitrate hexahydrate (19 mg, 0.064 mmol) in MeOH (5 mL), compound **39b** (69 mg, 0.032 mmol) in CH_2_Cl_2_ (5 mL) was added dropwise. The reaction mixture was sonicated at room temperature till all the solid was dissolved. The resulting mixture was removed and kept under reduced pressure until the weight was not changed. Compound **40b** was obtained as a white powder (82 mg, quant).

^1^H NMR (700 MHz, DMSO-*d*_6_) δ 8.46–8.40 (m, 2H, CH-Py./DPA), 8.33–8.29 (m, 1H), 8.23–8.17 (m, 1H), 8.02 (q, *J* = 7.3 Hz, 4H, CH-Py./DPA), 7.68–7.57 (m, 7H), 7.50 (dt, *J* = 14.2, 7.1 Hz, 2H, CH-Py./DPA), 7.44 (dt, *J* = 10.8, 7.5 Hz, 2H, CH-Py./DPA), 7.36–7.30 (m, 5H), 7.19–7.14 (m, 9H), 6.89 (s, 1H, CH-Ph./DPA), 6.61 (d, *J* = 11.2 Hz, 1H), 6.58–6.53 (m, 2H), 5.93 (s, 1H), 5.53 (dd, *J* = 15.0, 9.0 Hz, 1H), 5.31 (q, *J* = 6.8 Hz, 1H), 4.60 (d, *J* = 15.7 Hz, 2H), 4.51 (dd, *J* = 12.0, 2.8 Hz, 1H), 4.43 (t, *J* = 15.0 Hz, 3H), 4.31 (s, 1H), 4.25 (dd, *J* = 6.2, 3.0 Hz, 2H), 4.19–4.17 (m, 3H), 4.12 (s, 2H), 4.08–4.04 (m, 1H), 4.00 (d, *J* = 13.1 Hz, 1H), 3.90 (s, 3H, CH_3_-alkyl/DM1), 3.77 (d, *J* = 15.9 Hz, 2H), 3.70 (d, *J* = 16.7 Hz, 2H), 3.61 (t, *J* = 4.8 Hz, 1H), 3.57 (s, 2H), 3.54 (dd, *J* = 5.8, 3.7 Hz, 2H), 3.52–3.51 (m, 1H), 3.49 (d, *J* = 9.2 Hz, 2H), 3.24 (s, 3H, CH_3_-alkyl/DM1), 3.17 (d, *J* = 12.6 Hz, 1H), 3.13 (s, 2H), 2.92–2.88 (m, 1H), 2.85–2.74 (m, 6H), 2.70 (s, 3H, CH_3_-alkyl/DM1), 2.57–2.52 (m, 2H), 2.51–2.49 (m, 8H), 2.10 (dd, *J* = 9.0, 6.0 Hz, 2H), 2.04 (dd, *J* = 14.5, 2.8 Hz, 1H), 1.84–1.67 (m, 12H), 1.66–1.60 (m, 2H), 1.58 (s, 3H, CH_3_-alkyl/DM1), 1.46 (t, *J* = 12.2 Hz, 3H), 1.40 (d, *J* = 7.6 Hz, 6H), 1.25–1.23 (m, 3H), 1.17 (d, *J* = 6.8 Hz, 3H, CH_3_-alkyl/DM1), 1.11 (d, *J* = 6.4 Hz, 3H, CH_3_-alkyl/DM1), 1.04 (s, 4H), 0.94 (d, *J* = 6.6 Hz, 4H), 0.84 (dt, *J* = 19.7, 7.1 Hz, 4H), 0.78 (s, 3H, CH_3_-alkyl/DM1). ^13^C NMR (176 MHz, DMSO-*d*_6_) δ 178.5, 171.6, 170.5, 170.3, 169.3, 169.0, 168.2, 158.9, 155.3, 154.1, 152.3, 151.3, 147.1 (CH), 142.6 (CH), 141.3, 141.3, 140.5 (CH), 139.8, 139.7, 139.2, 138.4, 138.1, 137.9, 136.7, 133.8, 132.6 (CH), 129.0 (CH), 128.9 (CH), 128.5 (CH), 128.1 (CH), 127.5 (CH), 127.4 (CH), 127.2 (CH), 127.0 (CH), 126.7 (CH), 126.6 (CH), 125.2 (CH), 124.8 (CH), 124.4 (CH), 121.7 (CH), 120.6 (CH), 117.8 (CH), 114.7 (CH), 114.0 (CH), 88.2 (CH), 80.0, 77.8 (CH), 73.2 (CH), 70.3 (CH_2_), 70.3 (CH_2_), 70.0 (CH_2_), 69.9 (CH_2_), 69.7 (CH_2_), 69.6 (CH_2_), 69.5 (CH_2_), 69.3 (CH_2_), 69.0 (CH_2_), 67.5 (CH_2_), 66.9 (CH), 60.0 (CH_2_), 58.7 (CH_3_), 56.9 (CH_3_), 56.6 (CH_2_), 56.2, 55.6, 51.7 (CH), 50.5, 49.0, 47.2, 45.8, 45.4, 44.9, 41.9 (CH_2_), 41.4 (CH_2_), 37.7 (CH), 36.9 (CH_2_), 36.5 (CH_2_), 36.3 (CH_2_), 35.5 (CH_2_), 35.3 (CH_2_), 32.0 (CH_2_), 31.0 (CH_2_), 30.8 (CH_2_), 29.7 (CH_3_), 27.0 (CH_3_), 26.7 (CH_3_), 26.2 (CH_2_), 26.0 (CH_2_), 24.6 (CH_2_), 23.7 (CH_2_), 22.1 (CH_2_), 21.9 (CH_2_), 15.1 (CH_3_), 14.5 (CH_3_), 14.0 (CH_3_), 13.9 (CH_3_), 13.0 (CH_3_), 11.4 (CH_3_). HRMS (ESI): calc. for C_118_H_151_ClN_14_O_19_S_2_Zn^2+^: 2230.9690, found [M+Zn^2+^]^2+^: 1115.4845.

#### Methyl-4-({[4-(3,5-bis{[{[6-(hexanoylamino)pyridin-2-yl]methyl}(pyridin-2-ylmethyl)amino]methyl}phenoxy)butyl]amino}methyl)benzoate (**37b**)

Compound **11** (2.2 g, 2.7 mmol, 1.0 equiv.) and methyl 4-formylbenzoate (891.5 mg, 5.4 mmol, 2.0 equiv.) were dissolved in MeOH (27.2 mL), and the reaction solution was stirred at 80 °C overnight. Sodium borohydride (410.9 mg, 10.9 mmol, 4.0 equiv.) was added into the solution at 0 °C. After the reaction was completed, the solvent was removed. The residue was dissolved in CH_2_Cl_2_ and extracted with saturated NH_4_Cl_(aq)_. The combined organic extracts were dried over Na_2_SO_4_ and concentrated in vacuo. The residue was purified by flash chromatography over silica gel (5% MeOH in CH_2_Cl_2_) to give compound **37b** (1.73 g, 66%). ^1^H NMR (400 MHz, CDCl_3_) δ 8.83 (s, 2H, CH-Py./DPA), 8.52–8.46 (m, 2H, CH-Py./DPA), 8.12 (d, *J* = 8.3 Hz, 2H, CH-Py./DPA), 7.98 (d, *J* = 8.1 Hz, 2H), 7.67 (t, *J* = 7.9 Hz, 2H, CH-Py./DPA), 7.56 (td, *J* = 7.5, 1.7 Hz, 2H, CH-Py./DPA), 7.52–7.47 (m, 2H, CH-Py./DPA), 7.39 (d, *J* = 8.1 Hz, 2H), 7.29–7.27 (m, 1H), 7.26–7.23 (m, 2H), 7.16–7.09 (m, 2H, CH-Py./DPA), 6.73 (s, 2H, CH-Ph./DPA), 3.94 (t, *J* = 6.3 Hz, 2H), 3.91 (d, *J* = 0.7 Hz, 3H), 3.85 (s, 2H), 3.79 (s, 4H), 3.69 (s, 4H), 3.58 (s, 4H), 2.70 (t, *J* = 7.0 Hz, 2H), 2.07 (t, *J* = 7.6 Hz, 4H), 1.82 (q, *J* = 6.9, 6.2 Hz, 2H), 1.74–1.70 (m, 2H), 1.59–1.50 (m, 5H), 1.26–1.13 (m, 8H), 0.86–0.79 (m, 6H, CH_3_-alkyl/DPA). HRMS (ESI): calc. for C_57_H_72_N_9_O_5_^+^: 962.5651, found: 962.5651.

#### Methyl-4-[([4-(3,5-bis{[{[6-(hexanoylamino)pyridin-2-yl]methyl}(pyridin-2-ylmethyl)amino]methyl}phenoxy)butyl]{[1-(4-chlorophenyl)cyclohexyl]carbonyl}amino)methyl]benzoate (**38b**)

To compound **37b** (192.9 mg, 0.2 mmol, 1.0 equiv.) in CH_2_Cl_2_, triethylamine (5.9 mL, 42.1 mmol, 6.0 equiv.) and 1-(4-chlorophenyl)cyclohexanecarbonyl chloride (500 mg, 1.94 mmol, 5.0 equiv.) were added. The reaction solution was stirred at room temperature for 15 h. The reaction mixture was washed with saturated NH_4_Cl_(aq)_, dried over Na_2_SO_4_, and concentrated in vacuo. The residue was purified by flash chromatography over silica gel (5% MeOH in CH_2_Cl_2_) to give compound **38b** (141.4 mg, 60%). ^1^H NMR (400 MHz, CDCl_3_) δ 8.88 (s, 2H, CH-Py./DPA), 8.54–8.44 (m, 2H, CH-Py./DPA), 8.13 (d, *J* = 8.2 Hz, 2H, CH-Py./DPA), 7.94 (d, *J* = 8.0 Hz, 2H), 7.67 (t, *J* = 7.9 Hz, 2H, CH-Py./DPA), 7.55 (td, *J* = 7.6, 1.8 Hz, 2H, CH-Py./DPA), 7.48 (d, *J* = 7.8 Hz, 2H, CH-Py./DPA), 7.32 (s, 2H), 7.26–7.22 (m, 4H), 7.19 (d, *J* = 8.3 Hz, 2H), 7.15–7.10 (m, 2H, CH-Py./DPA), 7.01 (s, 1H, CH-Ph./DPA), 6.68 (s, 2H, CH-Ph./DPA), 3.90 (s, 3H), 3.78 (s, 4H), 3.70 (s, 4H), 3.58 (s, 4H), 2.04 (t, *J* = 7.5 Hz, 4H), 1.71–1.49 (m, 24H), 1.24–1.13 (m, 8H), 0.82 (t, *J* = 7.0 Hz, 6H, CH_3_-alkyl/DPA). HRMS (ESI): calc. for C_70_H_83_ClN_9_O_6_^-^: 1180.6160, found: 1180.6298.

#### *tert*-Butyl-(2-{2-[2-({4-[([4-(3,5-bis{[{[6-(hexanoylamino)pyridin-2-yl]methyl}(pyridin-2-ylmethyl)amino]methyl}phenoxy)butyl]{[1-(4-chlorophenyl)cyclohexyl]carbonyl}amino)methyl]benzoyl}amino)ethoxy]ethoxy}ethyl)carbamate (**41**)

To compound **38b** (948.2 mg, 0.8 mmol) in MeOH (16 mL), 0.5 N LiOH_(aq)_ was added. The reaction mixture was stirred at room temperature overnight. After the reaction was completed, the solvent was removed. The residue was redissolved in CH_2_Cl_2_ and extracted with 2 N HCl_(aq)_. The combined organic extracts were dried over Na_2_SO_4_ and concentrated in vacuo. The residue was purified by flash chromatography over silica gel (5% MeOH in CH_2_Cl_2_) to give compound **38b**_acid (795.2 mg, 85%). ^1^H NMR (400 MHz, CDCl_3_) δ 9.38 (s, 2H), 8.46 (d, *J* = 5.0 Hz, 2H, CH-Py./DPA), 8.10 (d, *J* = 8.2 Hz, 2H, CH-Py./DPA), 7.99 (d, *J* = 7.7 Hz, 2H, CH-Py./DPA), 7.61 (t, *J* = 7.9 Hz, 2H, CH-Py./DPA), 7.54 (td, *J* = 7.6, 1.8 Hz, 2H), 7.47–7.38 (m, 2H), 7.37–7.22 (m, 7H), 7.16 (d, *J* = 7.4 Hz, 2H, CH-Py./DPA), 7.09 (t, *J* = 6.4 Hz, 2H, CH-Py./DPA), 7.01–6.87 (m, 1H), 6.75–6.60 (m, 1H), 3.78 (s, 4H), 3.71 (s, 4H), 3.65 (s, 4H), 2.42–2.10 (m, 15H), 1.87–1.61 (m, 12H), 1.36–1.24 (m, 10H), 0.94–0.81 (m, 6H, CH_3_-alkyl/DPA). HRMS (ESI): *m/z* calc. for C_69_H_81_ClN_9_O_6_^-^: 1167.6037, found: 1167.6085.

A mixture of compound **38b**_acid (795.2 mg, 0.7 mmol, 1.1 equiv.), EDCI (195.1 mg, 1.0 mmol, 1.5 equiv.), and HOBt (137.9 mg, 1.0 mmol, 1.5 equiv.) in CH_2_Cl_2_ (13.7 mL) was stirred for 1 h at room temperature. A solution of {2-[2-(2-Amino-ethoxy)-ethoxy]-ethyl}-carbamic acid *tert*-butyl ester (253.4.0 mg, 1.0 mmol, 1.5 equiv.) and *N*-methylmorpholine (206.4 mg, 2.0 mmol, 3.0 equiv.) in CH_2_Cl_2_ (2.0 mL) was added to the reaction mixture, and the resultant reaction solution was stirred at room temperature for 15 h. The reaction mixture was then washed with saturated NH_4_Cl_(aq)_, dried over Na_2_SO_4_, and concentrated in vacuo. The residue was purified by flash chromatography over silica gel (5% MeOH in CH_2_Cl_2_) to give compound **41** (690.8 mg, 73%). ^1^H NMR (400 MHz, CDCl_3_) δ 8.91 (s, 2H), 8.53–8.45 (m, 2H, CH-Py./DPA), 8.12 (d, *J* = 8.3 Hz, 2H, CH-Py./DPA), 7.75–7.62 (m, 4H), 7.60–7.52 (m, 2H, CH-Py./DPA), 7.52–7.45 (m, 2H), 7.37–7.27 (m, 2H), 7.25–7.22 (m, 2H), 7.21–7.10 (m, 5H), 7.03–6.93 (m, 1H), 6.84–6.75 (m, 1H), 6.71–6.63 (m, 2H), 3.78 (s, 4H), 3.69 (s, 4H), 3.68–3.65 (m, 4H), 3.63 (s, 4H), 3.59–3.52 (m, 6H), 3.32–3.26 (m, 2H), 2.05 (t, *J* = 7.7 Hz, 4H), 1.74–1.64 (m, 20H), 1.53 (t, *J* = 7.4 Hz, 4H), 1.42 (s, 9H), 1.27–1.13 (m, 10H), 0.82 (td, *J* = 7.0, 1.3 Hz, 6H, CH_3_-alkyl/DPA). HRMS (ESI): *m/z* calc. for C_80_H_103_ClN_11_O_9_^–^: 1397.7668, found: 1397.7709.

#### (1*S*,2*R*,3*S*,5*S*,6*S*,16*E*,18*Z*,20*R*,21*S*)-11-Chloro-21-hydroxy-12,20-dimethoxy-2,5,9,16-tetramethyl-8,23-dioxo-4,24-dioxa-9,22-diazatetracyclo[19.3.1.1.^10,14^0^3,5^]hexacosa-10(26),11,13,16,18-pentaen-6-yl (22*S*)-1-{4-[([4-(3,5-bis{[{[6-(Hexanoylamino)pyridin-2-yl]methyl}(pyridin-2-ylmethyl)amino]methyl}phenoxy)butyl]{[1-(4-chlorophenyl)cyclohexyl]carbonyl}amino)methyl]phenyl}-17,17,21,22-tetramethyl-1,12,20-trioxo-5,8-dioxa-15,16-dithia-2,11,21-triazatricosan-23-oate (**42**)

To a solution of compound **41** in CH_2_Cl_2_ (1.0 mL), TFA (1.0 mL) was added. The reaction mixture was stirred at room temperature overnight. After the reaction was completed, the excess amount of TFA was removed under vacuum to give the Boc-deprotected product, which was used for the next reaction without further purification. A mixture of compound **17** (92.6 mg, 0.1 mmol, 1.2 equiv.), EDCI (42.0 mg, 0.2 mmol, 2.0 equiv.), and HOBt (29.6 mg, 0.2 mmol, 2.0 equiv.) in CH_2_Cl_2_ was stirred (1.2 mL) for 1 h at room temperature. The Boc-deprotected product (0.1 mmol, 1.0 equiv.) and *N*-methylmorpholine (66.6 mg, 0.7 mmol, 6.0 equiv.) in CH_2_Cl_2_ (1.0 mL) were added to the reaction mixture. The resultant reaction solution was stirred at room temperature overnight, washed with saturated NH_4_Cl_(aq)_, dried over Na_2_SO_4_, and concentrated in vacuo. The residue was purified by flash chromatography over silica gel (5% MeOH in CH_2_Cl_2_) to give compound **42** (97.3 mg, 47% in two steps). ^1^H NMR (700 MHz, CDCl_3_) δ 8.95–8.81 (m, 2H, CH-Py./DPA), 8.42 (dd, *J* = 5.2, 2.9 Hz, 2H, CH-Py./DPA), 8.05 (d, *J* = 8.3 Hz, 2H, CH-Py./DPA), 7.64 (d, *J* = 7.8 Hz, 2H, CH-Py./DPA), 7.60 (td, *J* = 7.9, 2.8 Hz, 2H, CH-Py./DPA), 7.48 (t, *J* = 7.8 Hz, 2H, CH-Py./DPA), 7.42 (d, *J* = 7.9 Hz, 2H), 7.22–7.15 (m, 6H), 7.14–7.09 (m, 2H, CH-Py./DPA), 7.06 (d, *J* = 6.5 Hz, 2H), 6.93–6.78 (m, 2H), 6.76 (s, 1H), 6.62 (dd, *J* = 21.1, 11.3 Hz, 3H), 6.56 (s, 1H), 6.35 (dd, *J* = 15.4, 11.0 Hz, 1H), 6.25 (d, *J* = 15.6 Hz, 1H), 5.61 (dd, *J* = 15.4, 9.1 Hz, 1H), 5.30 (q, *J* = 7.1 Hz, 1H), 4.75–4.68 (m, 1H), 4.49 (s, 1H), 4.21 (t, *J* = 11.4 Hz, 1H), 4.04 (s, 1H), 3.91 (d, *J* = 3.1 Hz, 3H), 3.83 (s, 1H), 3.71 (s, 3H), 3.62 (s, 3H), 3.60–3.57 (m, 6H), 3.55–3.53 (m, 2H), 3.51 (s, 3H), 3.47–3.44 (m, 2H), 3.41 (dd, *J* = 9.3, 3.6 Hz, 1H), 3.34 (s, 2H), 3.26–3.23 (m, 3H), 3.14 (d, *J* = 2.5 Hz, 3H), 3.05 (d, *J* = 12.8 Hz, 1H), 2.94 (dd, *J* = 9.8, 3.3 Hz, 1H), 2.80 (dd, *J* = 7.2, 3.0 Hz, 1H), 2.78 (d, *J* = 3.8 Hz, 3H), 2.56–2.51 (m, 1H), 2.41 (dd, *J* = 9.3, 5.7 Hz, 2H), 2.27–2.20 (m, 2H), 2.13–2.08 (m, 2H), 2.00 (s, 4H), 1.95–1.89 (m, 2H), 1.81–1.75 (m, 3H), 1.61 (s, 2H), 1.57 (s, 5H), 1.48–1.46 (m, 3H), 1.39 (dt, *J* = 10.4, 3.5 Hz, 1H), 1.22 (t, *J* = 6.0 Hz, 8H), 1.20–1.18 (m, 6H), 1.16 (d, *J* = 3.5 Hz, 4H), 1.14 (d, *J* = 3.5 Hz, 4H), 1.10–1.08 (m, 3H), 1.00–0.96 (m, 1H), 0.89 (dd, *J* = 6.9, 3.0 Hz, 1H), 0.83–0.78 (m, 12H), 0.76 (dd, *J* = 7.6, 3.1 Hz, 6H, CH_3_-alkyl/DPA), 0.73 (d, *J* = 2.1 Hz, 3H). ^13^C NMR (176 MHz, CDCl_3_) δ 174.6, 172.3, 172.3, 171.0, 170.9, 168.9, 159.7, 157.9, 156.1, 152.4, 151.6, 149.0 (CH), 144.7, 142.3, 141.2, 140.2, 139.2, 139.1 (CH), 136.6 (CH), 133.3 (CH), 132.4, 129.3 (CH), 129.0 (CH), 128.0 (CH), 127.8 (CH), 127.5 (CH), 127.4 (CH), 127.2 (CH), 126.8 (CH), 126.5 (CH), 125.5 (CH), 123.0 (CH), 122.3 (CH), 122.2 (CH), 119.1 (CH), 118.8, 113.9 (CH), 113.3 (CH), 112.5 (CH), 88.7 (CH), 80.9, 78.2 (CH), 74.2 (CH), 70.3 (CH_2_), 69.9 (CH_2_), 67.6, 67.3 (CH), 66.8, 60.2 (CH_2_), 60.1, 59.5 (CH_2_), 58.2 (CH_2_), 56.7 (CH_3_), 52.6 (CH), 51.0, 50.5, 49.0, 47.6, 46.8 (CH_2_), 39.9 (CH_2_), 39.4 (CH_2_), 39.0 (CH), 37.5 (CH_2_), 37.0 (CH_2_), 36.4 (CH_2_), 36.3 (CH_2_), 35.7 (CH_3_), 34.8 (CH_2_), 32.5 (CH_2_), 31.7 (CH_2_), 31.4 (CH_2_), 31.1 (CH_2_), 29.8 (CH_2_), 29.4 (CH_2_), 29.2 (CH_2_), 28.0 (CH_3_), 26.0 (CH_2_), 25.4 (CH_2_), 25.0 (CH_2_), 23.1 (CH_2_), 22.4 (CH_2_), 15.6 (CH_3_), 14.7 (CH_3_), 14.3 (CH_3_), 14.0 (CH_3_), 13.5 (CH_3_), 12.3 (CH_3_), 11.6 (CH_3_). HRMS (ESI): calc. for C_116_H_151_Cl_2_N_14_O_18_S_2_^-^: 2162.0155, found: 2162.0033.

#### (1*S*,2*R*,3*S*,5*S*,6*S*,16*E*,18*Z*,20*R*,21*S*)-11-Chloro-21-hydroxy-12,20-dimethoxy-2,5,9,16-tetramethyl-8,23-dioxo-4,24-dioxa-9,22-diazatetracyclo[19.3.1.1.^10,14^0^3,5^]hexacosa-10(26),11,13,16,18-pentaen-6-yl (22*S*)-1-{4-[([4-(3,5-bis{[{[6-(Hexanoylamino)pyridin-2-yl]methyl}(pyridin-2-ylmethyl)amino]methyl}phenoxy)butyl]{[1-(4-chlorophenyl)cyclohexyl]carbonyl}amino)methyl]phenyl}-17,17,21,22-tetramethyl-1,12,20-trioxo-5,8-dioxa-15,16-dithia-2,11,21-triazatricosan-23-oate·2[Zn(NO_3_)_2_] (**43**)

^1^H NMR (700 MHz, DMSO-*d*_6_) δ 8.50–8.41 (m, 2H, CH-Py./DPA), 8.00 (dt, *J* = 37.9, 6.8 Hz, 4H, CH-Py./DPA), 7.82–7.37 (m, 7H), 7.35–6.99 (m, 10H), 6.89 (s, 1H, CH-Ph./DPA), 6.64–6.49 (m, 2H), 5.93 (d, *J* = 1.6 Hz, 1H), 5.57 (dd, *J* = 15.0, 9.0 Hz, 1H), 5.31 (q, *J* = 6.8 Hz, 1H), 4.60–4.35 (m, 4H), 4.22–3.89 (m, 9H), 3.75 (dd, *J* = 49.1, 15.9 Hz, 4H), 3.54–3.41 (m, 22H), 3.37 (d, *J* = 6.1 Hz, 17H), 3.26–3.13 (m, 6H), 3.09 (s, 2H), 2.79–2.68 (m, 7H), 2.35 (q, *J* = 7.4 Hz, 2H), 2.31–2.10 (m, 3H), 2.03 (dd, *J* = 14.5, 2.8 Hz, 1H), 1.88 (td, *J* = 12.7, 11.8, 4.7 Hz, 1H), 1.84–1.76 (m, 3H), 1.73–1.51 (m, 12H), 1.48–1.32 (m, 9H), 1.29–1.21 (m, 4H), 1.17–1.13 (m, 5H), 1.13–1.09 (m, 5H), 0.92 (d, *J* = 6.3 Hz, 4H), 0.77 (s, 3H, CH_3_-alkyl/DM1). ^13^C NMR (176 MHz, DMSO-*d*_6_) δ 172.2, 171.4, 170.7, 169.9, 168.2, 159.0, 158.0, 155.3, 151.4, 151.3, 148.4 (CH), 141.4, 141.3, 138.5 (CH), 138.2, 136.5 (CH), 132.5 (CH), 132.3, 128.6 (CH), 127.2 (CH), 127.1 (CH), 126.1 (CH), 125.3 (CH), 122.4 (CH), 122.2 (CH), 121.6 (CH), 120.7 (CH), 117.2 (CH), 117.1, 113.9 (CH), 113.2 (CH), 111.5 (CH), 88.2 (CH), 80.1, 77.7 (CH), 73.2, 69.6 (CH_2_), 69.1 (CH_2_), 68.9 (CH_2_), 66.8 (CH), 60.0, 59.2 (CH_2_), 59.0 (CH_2_), 57.6, 56.5 (CH_3_), 56.1 (CH_3_), 51.6 (CH), 50.2, 45.5 (CH_2_), 38.7 (CH_2_), 37.4 (CH), 36.4 (CH_2_), 36.0 (CH_2_), 35.6 (CH_2_), 35.4 (CH_2_), 35.0 (CH_2_), 32.0 (CH_2_), 30.8 (CH_2_), 29.7 (CH_3_), 28.6 (CH_2_), 27.4 (CH_3_), 26.9 (CH_3_), 24.7 (CH_2_), 21.9 (CH_2_), 15.0 (CH_3_), 14.4 (CH_3_), 13.8 (CH_3_), 13.0 (CH_3_), 11.4 (CH_3_). HRMS (ESI): calc. for C_116_H_153_Cl_2_N_18_O_30_S_2_Zn_2_^+^: 2539.8396, found: 2539.8460.

### Cell Culture/Viability Assay/Data analysis

MIA PaCa2 cells or HCC1806 cells were grown in RPMI 1640 medium, and Detroit 551 cells were grown in Dulbecco’s modified Eagle’s medium. Growth media of Detroit551 were supplemented with the following: 10% fetal bovine serum, 50 U/mL of streptomycin and penicillin, and 1% nonessential amino acids. The MTS assay was performed to examine cell viability. With cells (2500–3000 cells/well) in flat-bottom 96-well plates for 24 h growth, to the medium was then added the serially diluted compound and the cells were further incubated for 72 h. At the end of the 72 h incubation period, media were removed and a 100 μL mixture solution including MTS and PMS was added. Incubation of the cells for 1.5 h at 37 °C in a humidified incubator with 5% CO_2_ was carried out to convert the tetrazolium salt into formazan by the viable cells. The conversion to formazan was measured by absorbance (490 nm) using a BioTek PowerWave-X Absorbance microplate reader. The collected data were normalized using DMSO-treated controls (100% viability) and background controls (0% viability) to verify growth inhibition, while the IC_50_ value was calculated as the amount of compound that resulted in a 50% reduction in cell viability in comparison with DMSO-treated controls using GraphPad Prism version 4 software (San Diego, CA, USA).

### Biacore SPR binding assay

Phospholipids, 1,2-dioleoyl-*sn*-glycero-3-phosphocholine (DOPC), and 1,2-dioleoyl-*sn*-glycero-3-[phospho-l-serine] (DOPS) were obtained from Avanti Polar Lipids (Alabaster, AL) in chloroform solutions. These stock solutions were combined to the indicated ratios. To a round-bottom flask, a 0.4 mL aliquot of lipid solution at concentration of 10 mg/mL was added and evaporated under a N_2_ gas stream to furnish a thin lipid film. Rehydration of lipid films was carried out in PBS buffer for at least 1 h at room temperature. Resulting suspensions were extruded through a 100 nm polycarbonate filter using an Avanti MiniExtruder following the manufacturer’s instructions. Zeta potential (ZP) and liposomes’ size distributions were recorded by dynamic light scattering (DLS) and microelectrophoresis using a Zetasizer Nano ZS instrument. Then, using a Biacore T200 biosensor equipped with an L1 sensor chip (GE Healthcare), binding kinetics between the conjugates and liposome were recorded at 25 °C. Preconditioning of new sensor chips was performed with running buffer (5% DMSO in phosphate-buffered saline (PBS) with final pH 7.4) and two consecutive 30 s pulses of 2:3 v/v 50 mM HCl/isopropanol at a flow rate of 30 μL/min. A fresh liposome capture plate was prepared for each binding cycle. In PBS buffer, liposomes were diluted to 0.5–1 mM and captured to saturation (30–150 s) across isolated flow cells at 2–5 μL/min. In a single injection, conjugates were first diluted with running buffer and injected over lipid surfaces. At flow rate of 30 μL/min, association and dissociation phases were examined for 60s. At the end of each binding cycle, the surface was regenerated by injecting 2:3 v/v 50 mM HCl/isopropanol and equilibrated with running buffer before the next injection of the test compound. By subtracting SPR signals from a reference flow cell (DOPC immobilized surface), unspecific binding was removed. Using the bivalent analyte model, sensograms were fit globally with BIAcore T200 evaluation software 3.0.

### Pharmacokinetic Studies of Conjugates

Six-week-old male ICR mice, from the Biolasco Taiwan, were divided into groups of three and dosed at 5 mg/kg intravenously. Blood samples were drawn from each animal at time points of 0.003, 0.083, 0.25, 0.5, 1, 2, 4, 6, 8, and 24 h and stored on ice (0–4 ^o^C). With centrifugation (3000 rpm for 15 min at 4 °C in a Beckman Model Allegra 6R centrifuge), plasma was separated from the blood and stored in frozen conditions (−20 °C). In addition, mice bearing HCC1806 tumor were i.v. administered with cytotoxic payload **14** of 0.6 mg/kg and conjugate **40a** of 2 mg/kg (in 10% DMA/20% Cremophor EL/70% (5% dextrose)) when the mean tumor volume was approximately at the range of 500–900 mm^3^. The mice were sacrificed, and the blood samples of 0.5 mL each and tumor samples were collected at 0.5, 2, 6, 24, 72, and 168 h after administration. Each time point group included 4 mice. Mouse blood samples were collected in EDTA tubes and centrifuged at 13,000 rpm for 5 min at 4°C for plasma collection. Plasma and the harvested tumor samples were stored at −80°C until use. Fifty microliters of mouse plasma or the sample of tumor homogenated in ddH_2_O with dilution ratio of 1:3 (w/v) by MiniBeadbeater-16 (BioSpec Products Inc., OK, USA) was mixed with 100 μL of acetonitrile containing 250 ng/mL BPR0L187. The mixture was vortexed for 30 s and then centrifuged at 15000*g* for 20 min. The supernatant was transferred to a clean tube, and 15 μL of the supernatant was injected onto LC/MS/MS. Plasma samples were analyzed by liquid chromatography tandem mass spectrometry (LC/MS/MS). The chromatographic system Agilent 1200 series LC system and an Agilent ZORBAX Eclipse XDB-C_8_ column (5 μm, 3.0 × 150 mm) interfaced to an MDS Sciex API4000 tandem mass spectrometer equipped with an ESI in the positive scanning mode at 600 °C was used. Data acquisition was collected via multiple reactions monitoring (MRM). A gradient system was employed for the separation of analyte and IS. Mobile phase A was 10 mM ammonium acetate aqueous solution containing 0.1% formic acid. Mobile phase B was acetonitrile. The gradient profile was as follows: 0.0–1.1 min, 50% B; 1.2–3.7 min, 55%B–90%B; 3.8–5.0 min, 90%B–50%B. The flow rate was 1.5 mL/min. The autosampler was programmed to inject 15 μL of the sample every 5 min.

### IVIS Imaging of HCC1806 Tumor with Zn11-794

HCC1806 tumor-bearing mice were used when the mean tumor volume reached around 500–700 mm^3^. Tumor volume in mm^3^ was calculated by the following formula: volume = (length × width^2^)/2 and measured with a digital caliper. Untargeted Dye 794 or Zn11-794 (in 10% DMA/20% Cremophor EL/70% (5% dextrose)) was i.v. administered at 2 mg/kg. All treated mice were imaged by using an IVIS spectrum system at 24, 48, and 72 h. Briefly, the mice were anesthetized by 2.5% isoflurane inhalation and placed on the stage of IVIS apparatus with imaging conditions set as follows: excitation filter, 745 nm; emission filter, 820 nm; exposure time, auto; bin, 8 (medium); f/stop, 2; field of view, 22.7 cm. Using Living Image 4.5 software (PerkinElmer, Alameda, CA, USA), fluorescence intensity was quantified and the image was processed.

### Animal Studies

Cancer cells, suspending in phenol red free medium/DPBS, were mixed with Matrigel (356237, BD Biosciences, San Jose CA, USA) in a 1:1 ratio. Human pancreatic cancer MIA PaCa-2 (1 × 10^6^ cells) or triple-negative breast cancer HCC1806 (1 × 10^6^cells) cells were subcutaneously inoculated to left flanks of male nude mice 6 weeks old (Biolasco, Taiwan) or female nude mice 6 weeks old by using a 1 mL syringe (needle 24G × 1 in., 0.55 × 25 mm; TERUMO). Tumor dimensions were measured twice a week with an electronic caliper (FOW54-200-777, PRO-MAX, Newton, Massachusetts, USA), and the volume of the subcutaneously growing tumor in mm^3^ was calculated by the following formula: volume = (length × width^2^)/2. Conjugates were formulated 10% DMA/20% Cremophor EL/70% injectable solution of 5% dextrose (D5W) for treating MIA PaCa-2 and HCC1806 xenograft tumors. The MIA PaCa-2 or HCC1806 tumor-bearing mice were grouped, and conjugates were administered when the mean tumor volume was approximately at 200–250 mm^3^ or 600–700 mm^3^ (large tumor) with dose regimens: conjugate **40a** of 1 mg/kg or 2 mg/kg and cytotoxic payload **14** of 0.3 mg/kg at twice (day 1 and day 4) a week for 2 weeks. For the studies with large tumors, a weekly dose of conjugate **40a** of 2.5 mg/kg was used. Body weight of the mice and tumor volume were measured twice weekly.

In addition, a previously reported oncogene-induced, sorafenib-resistant HCC mouse model^50^ was used in the current study to examine the antitumor activities of synthesized compounds. Male C57BL/6j mice 4–5 weeks old were purchased from the National Laboratory Animal Center (Taipei, Taiwan) and kept in the laboratory animal center (LAC) of NHRI. The mice received 2 μg of pCMV(CAT)T7-SB100 (Addgene #34879), 10 μg of pT/Caggs-NRASV12 (Addgene #20205), and 10 μg of pKT2/CLP-AKT-LUC plasmids through hydrodynamic injection and were monitored for tumor growth weekly using IVIS until the development of HCC in the liver. The mouse number used in each experiment was indicated in the figures/legends. The animal study was reviewed and approved by the NHRI IACUC (Institutional Animal Care and Use Committee). HCC-bearing mice with the total flux from IVIS imaging above 1 × 10^9^ photons/s were used for treatment of conjugates. Indicated conjugate **40a** was dosed intravenously at 1 mg/kg with a frequency of twice (day 1 and day 4) a week for 2 weeks. Repetitive IVIS imaging was performed to track tumor progression, and tumor tissues of the treated HCC-bearing mice were collected at day 3, day 7, and day 14 post conjugate administrations for histological examination and RNA extraction. The tumor tissues of vehicle-treated HCC-bearing mice were collected at day 14 post treatment to serve as control samples.

### Repeat-Dose Toxicity Study

Male SD rats 8 weeks old (*n* = 5 per group) were i.v. bolus-administered with control vehicle (2.5 mL/kg) and 1 mg/kg **40a** once a week for 4 weeks (days 1, 8, 15, and 22). The animal body weights were measured daily during the study period. At the end of the study on day 29, all the animals were euthanized with 100% CO_2_ and sacrificed for organ harvest followed by organ weight measurements and for blood sample collection followed by hematology and serum chemistry assays. Hematology and serum chemistry parameters were determined using a HEMAVET 950 automated analyzer (Drew Scientific, Santa Clara, CA, USA) and a FUJI DRI-CHEM NX500 automated analyzer (FUJIFILM, Tokyo, Japan), respectively.

### Immunohistochemical Staining

Paraffin-embedded liver/tumor tissue sections were deparaffinized, rehydrated, underwent heat-induced antigen retrieval, and then incubated with primary Abs. The primary Abs types used for detection of Ki-67, F4/80, Gr-1, and CD8alpha were SP6 (Abcam), BM8 (Biolegend), RB6F8C5 (Biolegend), and D4W2Z (Cell Signaling Technology), respectively. ImmPRESS anti-rat Ig, ImmPRESS anti-rabbit Ig, polymer detection kits, DAB peroxidase substrate kit, (Vector laboratories) liquid permanent red substrate (Dako), and Hematoxylin Gill II (Leica) were used for detection and visualization. The images were captured using an automatic digital slide scanner Pannoramic MIDI with a Plan-Apochromat 20×/0.8 objective (3D HISTECH) by the Pathology Core Laboratory of NHRI.

### Nanostring Analysis

Total RNA was extracted from conjugate **40a**-treated or vehicle-treated tumor tissues of HCC-bearing mice with the RNA-easy kit (QIAGEN). The concentration (absorbance at 260 nm) and purity (A260/280 and A260/230 ratios) of the extracted RNA were measured by spectrophotometry, and the integrity of the RNA was further determined by a 2100 Bioanalyzer system (Agilent Technologies). The RNA, hybridized with barcoded probes (NanoString Technologies) provided in the nCounter Mouse PanCancer Immune Profiling panel kit, was then used for measurement of the mRNA expression of 770 genes related to immune responses. Nanostring nSolver 4.0 and nCounter advanced analysis 2.0 software (NanoString Technologies) were used for data processing, immune cell profiling, and pathway scoring according to developer’s instructions.

### Ethical Approval

Animals used in this study were maintained and treated according to the animal protocols (NHRI-IACUC-106076-A, NHRI-IACUC-107045-A, NHRI-IACUC-107130-A, NHRI-IACUC-109063, and NHRI-IACUC-108091-A) that were approved by NHRI IACUC.

### Statistical Analysis

GraphPad Prism 7 (GraphPad Software, La Jolla, USA) and Student’s *t* test were used for statistical analysis.

## Supporting Information Available

Synthesis of linkers and precursors, ^1^H and ^13^C spectra of the final conjugates, and HPLC chromatograms of conjugates used for *in vivo* studies (PDF)
Molecular formula strings (CSV)

## ABBREVIATIONS USED

ADCs: antibody-drug conjugates;
CAIX: carbonic anhydrase IX
CAR-T: chimeric antigen receptor T-cell immunotherapy
CCL5: CC ligand 5
CXCL10: C-X-C motif ligand 10
CL: clearance
DAMPs: danger-associated molecular patterns
DCs: dendritic cells
GST: glutathione *S*-transferases
HCC: hepatocellular carcinoma
IHC: immunohistochemical
PS: phosphatidylserine
SMDCs: small molecule drug conjugates
STAT1: signal transducer and activators of transcription 1
TIS: tumor inflammation signature
TNBC: triple-negative breast cancer
TME: tumor microenvironment
Zn-DPA: zinc(II)-bis-dipicolylamine
