# Supplementary material for: Targeting the Phosphatidylserine-Immune Checkpoint with a Small-Molecule Maytansinoid Conjugate
Source: J Med Chem. 2022 Sep 26;65(19):12802–24. doi: 10.1021/acs.jmedchem.2c00631 (PMC9574934; doi:10.1021/acs.jmedchem.2c00631)
Supplement: Supplementary file 1 — jm2c00631_si_001.pdf [file jm2c00631_si_001.pdf]

Supporting Information

Targeting Phosphatidylserine-Immune Checkpoint with a Small-Molecule Maytansinoid Conjugate

Chen-Fu Lo[1#], Tai-Yu Chiu[1#], Yu-Tzu Liu[2#], Pei-Yun Pan[1#], Kuan-Liang Liu [1], Chia-Yu Hsu[1], Ming-Yu Fang[1], Yu-Chen Huang[1], Teng-Kuang Yeh[1], Tsu-An Hsu[1], Chiung-Tong Chen *[1], Li-Rung Huang*[2], and Lun Kelvin Tsou*[1]

[1] Institute of Biotechnology and Pharmaceutical Research, National Health Research Institutes, Miaoli 35053, Taiwan, ROC

[2] Institute of Molecular and Genomic Medicine, National Health Research Institutes, Miaoli 35053, Taiwan, ROC

Correspondence and requests for materials should be addressed to C.T.C (ctchen@nhri.edu.tw), L.R.H. (lrhuang@nhri.edu.tw), and L.K.T. (kelvintsou@nhri.edu.tw)

Table of Contents

Figure S1-S5 ……………………………………..…………………………….……......... S2 – S7

Scheme S1-S3…….…………….………..………..……………..…......………………....... S8 – S10

Synthetic procedures and characterizations for compounds **2-21** and **Zn11-794**…….…… S11 – S21

Copies of ^{1}H, ^{13}C NMR spectra, mass spectra and HPLC traces ………............................... S22 - S57

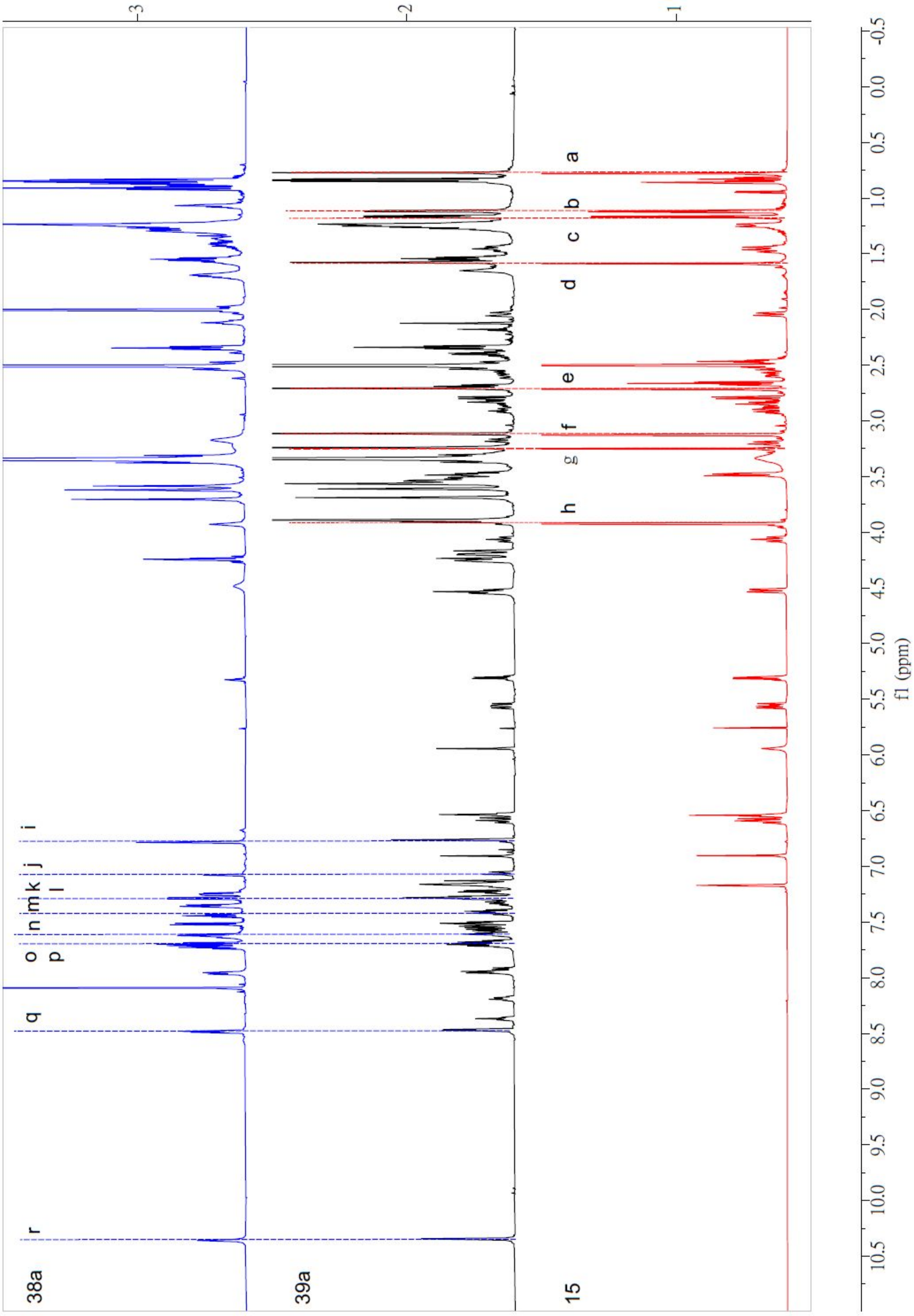
38a
39a
15
a
b
c
d
e
f
g
h
i
j
k
l
m
n
o
p
q
r
f1 (ppm)

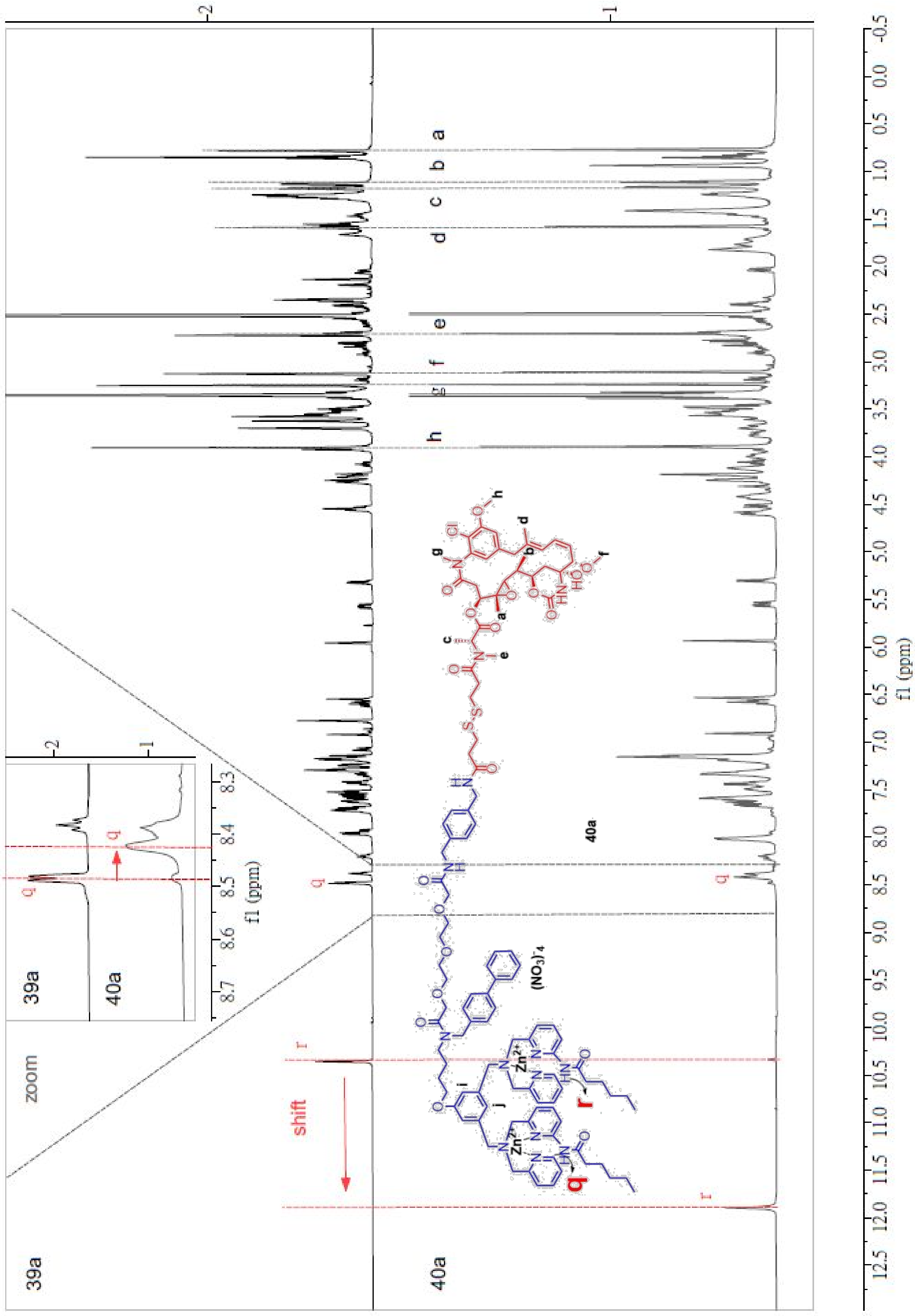

Figure S1. Analysis of NMR signal patterns and characterization of lead compound **40a**, and its intermediates **39a, 38a**, and **15**.

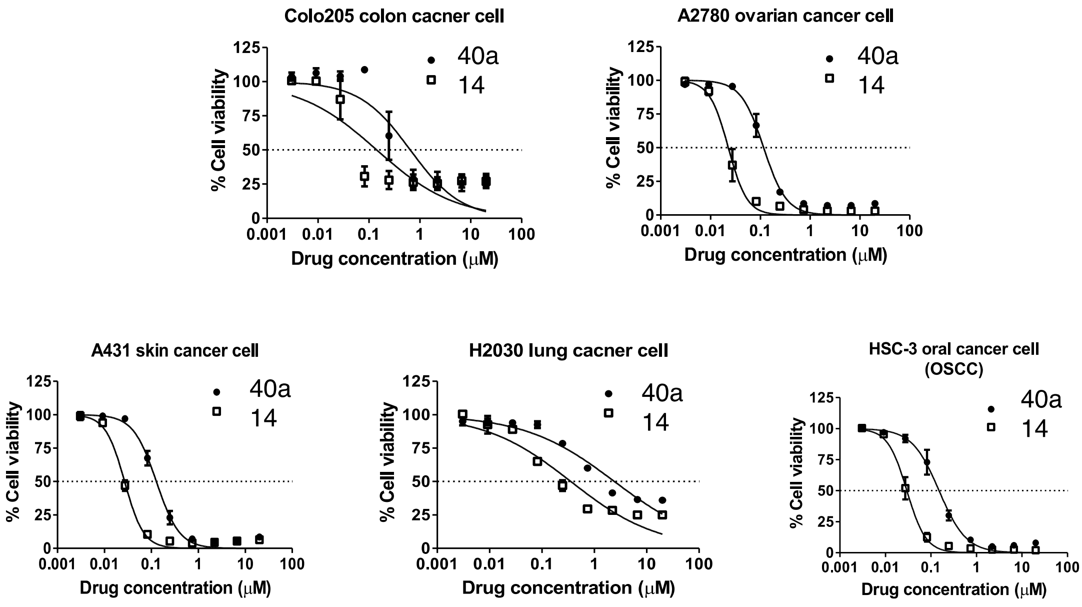

Cancer type IC50 (nM)	Colon Colo205	Ovary A2780	Skin A431	Lung H2030	Oral HSC-3
40a	902	117	129	2425	152
14	196	23	27	363	30

Figure S2. Cytotoxic effects and IC_{50}'s of conjugate **40a** and payload **14** on different cancer cells.

Cpd. 24

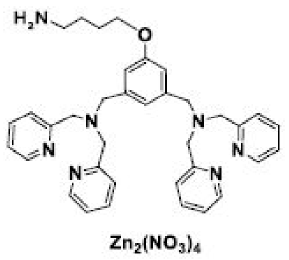

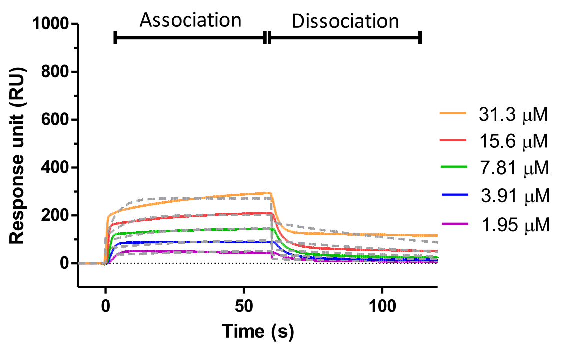

Cpd. 11

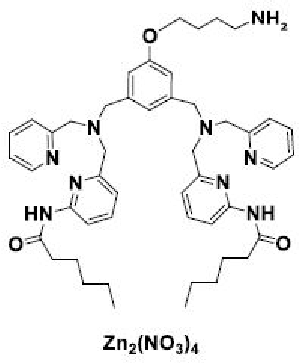

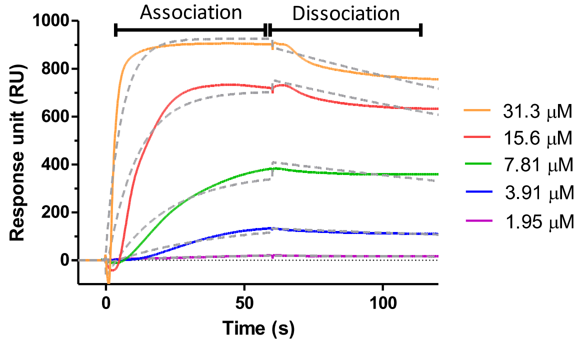

Curve	ka (1/Ms)	kd (1/s)	KD (M)
Cpd. 11	5562	0.00358	6.44E-07
Cpd. 24	7523	0.0113	1.50E-06

Figure S3. ***In vitro*** **SPR PS-association studies.** Sensorgrams generated using a Biacore T200. Zinc-coordinated dipicolylamine **24** and modified analog **11** were analyzed across a two-fold concentration series descending from 31.3 μM. The association was monitored for 1 min, and the dissociation time was 1 min. Liposome (DOPC/ DOPS (3:1,v/v)) was immobilized on a L1 chip at 5000 RU, where liposome of (DOPC 100%) was used as nonspecific binding control.

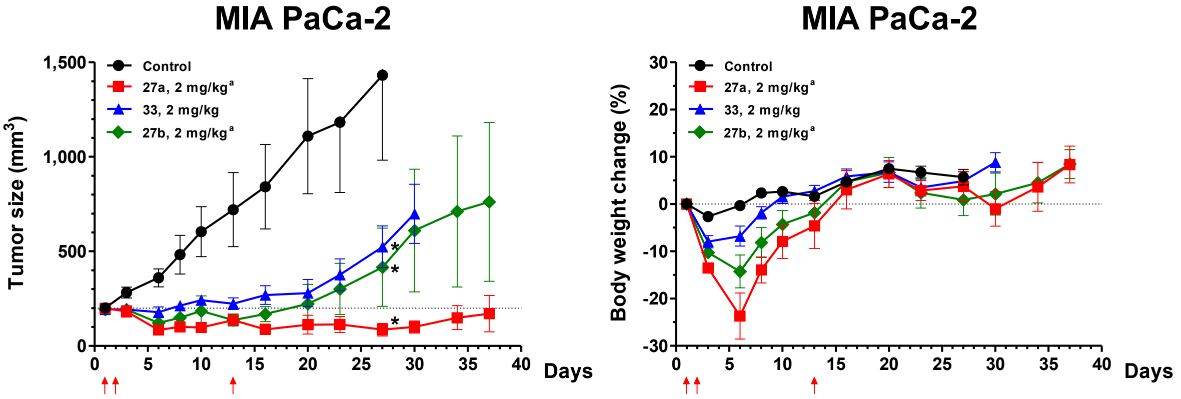

Figure S4. *In vivo* anti-tumor efficacies. Treatment regimen was presented as the amount in mg/kg and the dosage frequency per week. Comparisons of anti-MIA Paca-2 pancreatic cancer activities and body weight changes between conjugate **27**, **27b**, and **33** when administered intravenously at time-point illustrated with red arrows.

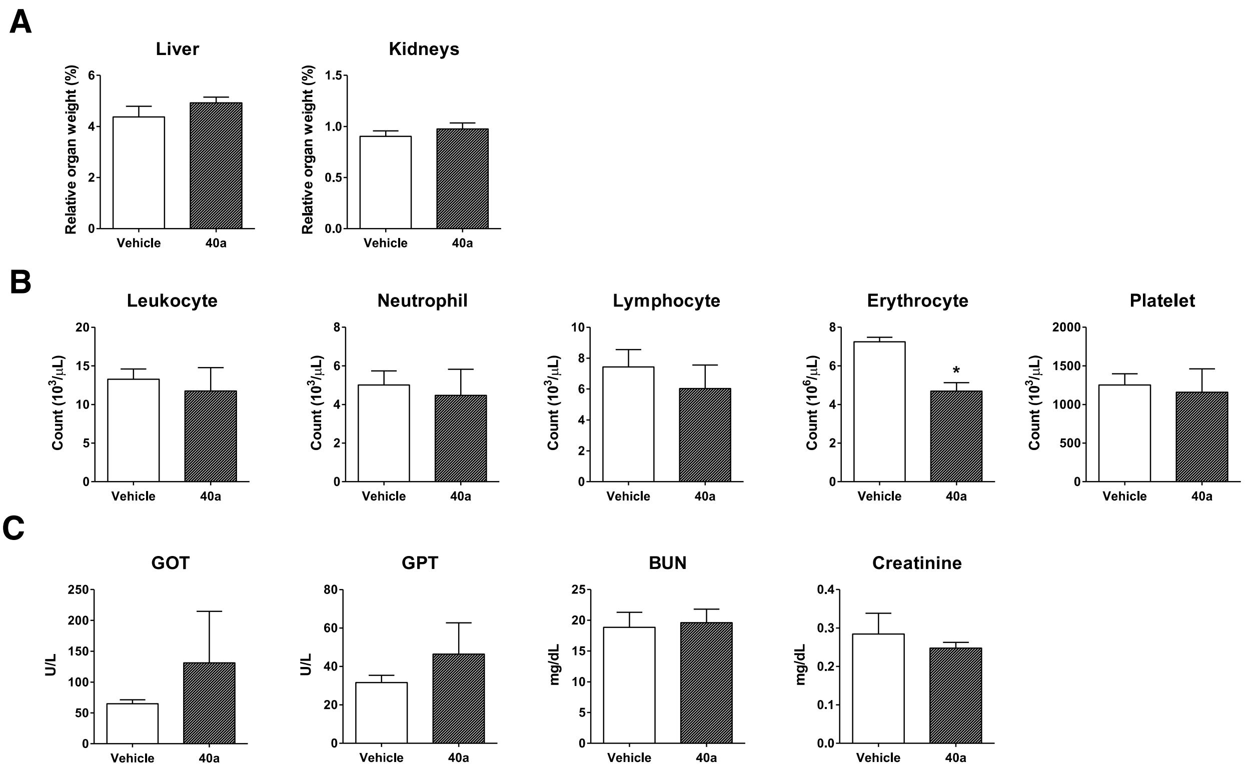

Figure S5. Analysis of a pilot toxicity (28-day repeat dose) study of lead compound **40a** in rat. (A) Compared to the vehicle control, treatment of **40a** did not alter organ weights (liver and kidneys). (B) Hematologic (Leukocyte, neutrophil, lymphocyte, and platelet) parameters were normal, except a reduction of erythrocyte count was observed. (C) Biochemical parameters, such as GOT and GPT, were slightly increased (not significant); level of BUN and creatinine were normal in the **40a** treatment group.

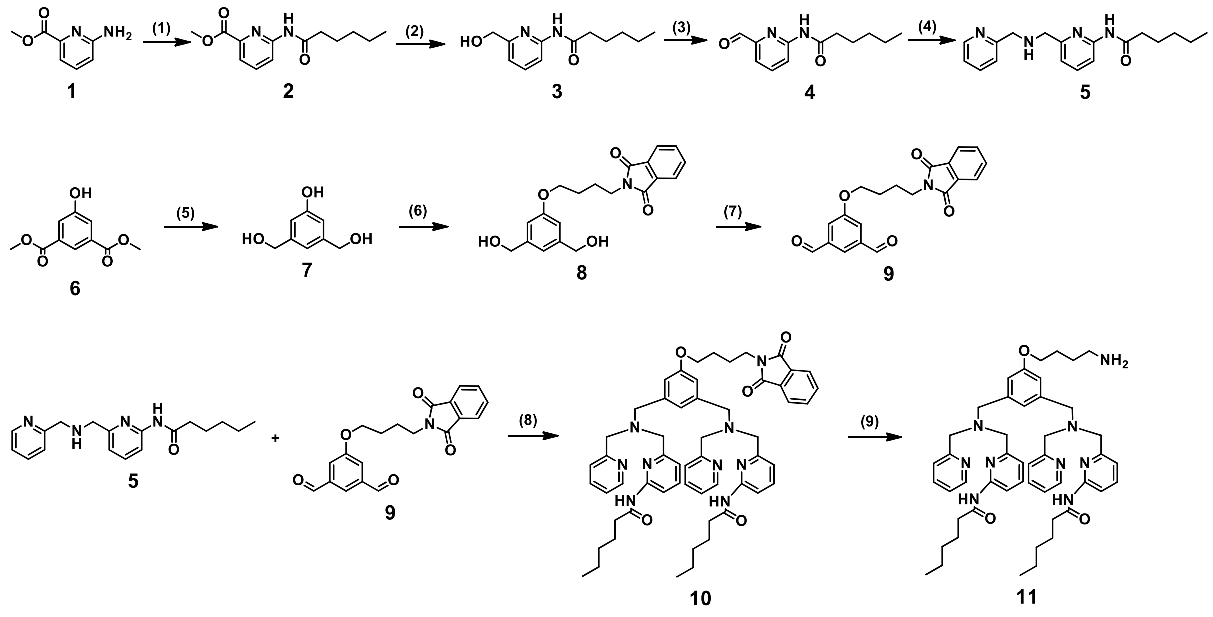

Reagents and conditions: (1) hexanoic acid, EDCI, DMAP, CH_2Cl_2, 16 h; (2) $NaBH_4$, MeOH, 4 h; (3) MnO_2, CH_2Cl_2, 40 °C, 15 h, 70% in three steps; (4) 2-Picolylamine, MeOH, rt, 3 h; then $NaBH_4$, 15 h, 90%; (5) $LiAlH_4$, THF, 50 °C, 15 h, 97%; (6) 2-(4-bromobutyl)isoindoline-1,3-dione, K_2CO_3, DMF, 40 °C, 14 h, 44%; (7) MnO_2, CH_2Cl_2, 15 h, 80%; (8) $NaB(OAc)_3H$, CH_2Cl_2, 18 h; (9) hydrazine, EtOH, 15 h, 61% in two steps.

Scheme S1. Synthetic procedures for modified dipicolylamine analog **11**.

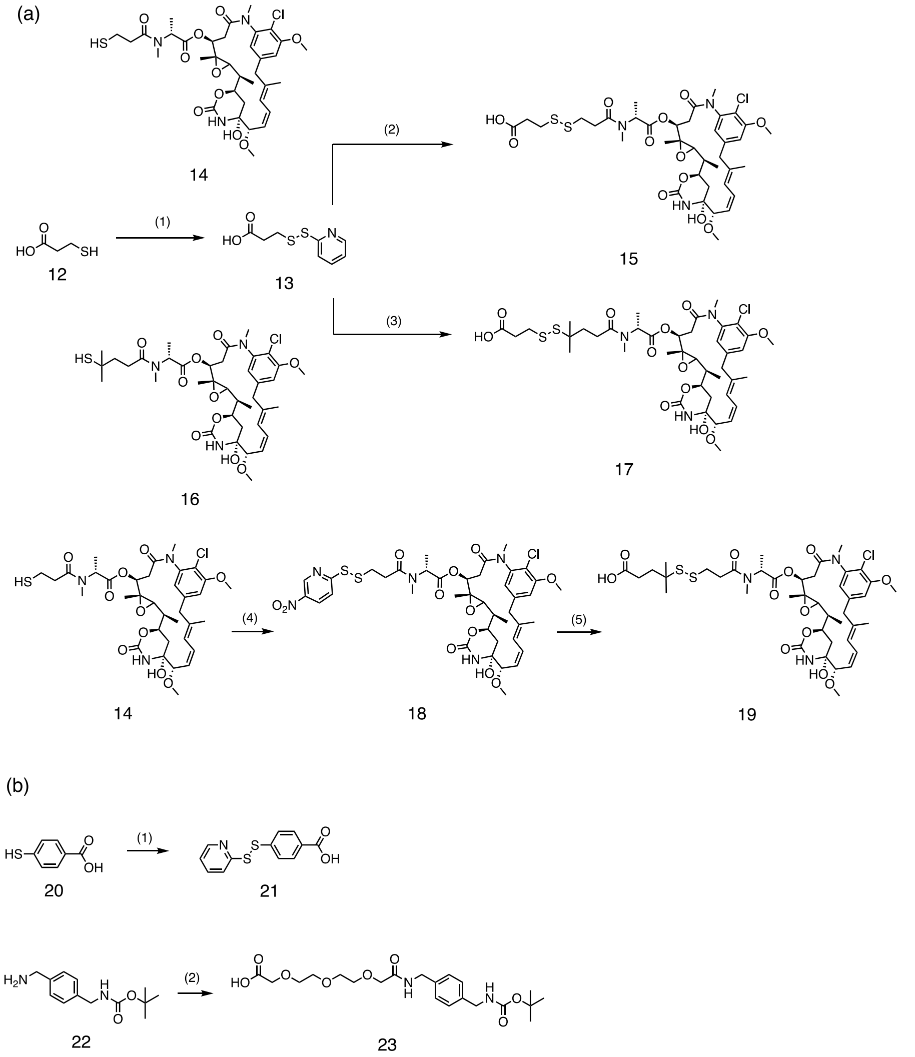

Reagents and conditions: (a) (1) 2,2'-dipyridyl disulfide, MeOH, 15 h, 74%; (2) compound 14, CH_2Cl_2, 35 °C, 16 h, 85%; (3) compound 16, MeOH, 50 mM potassium phosphate buffer pH 7.5, rt, 15 h, 71%; (4) 2,2′-dithiobis(5-nitropyridine), NMM, DMF,THF, 15 h, 83%; (5) 4-mercapto-4-methylpentanoic acid, DMF, THF, 50 mM potassium phosphate buffer pH 7.5, rt, 15 h, 29%; (b) (1) 2,2'-dipyridyl disulfide, MeOH, rt, 3 h, 24%; (2) 3,6,9-trioxaundecanedioic acid, EDCI, CH_2Cl_2, 0 °C to rt, 18 h.

Scheme S2. Synthetic procedures for linkers and maytansinoids.

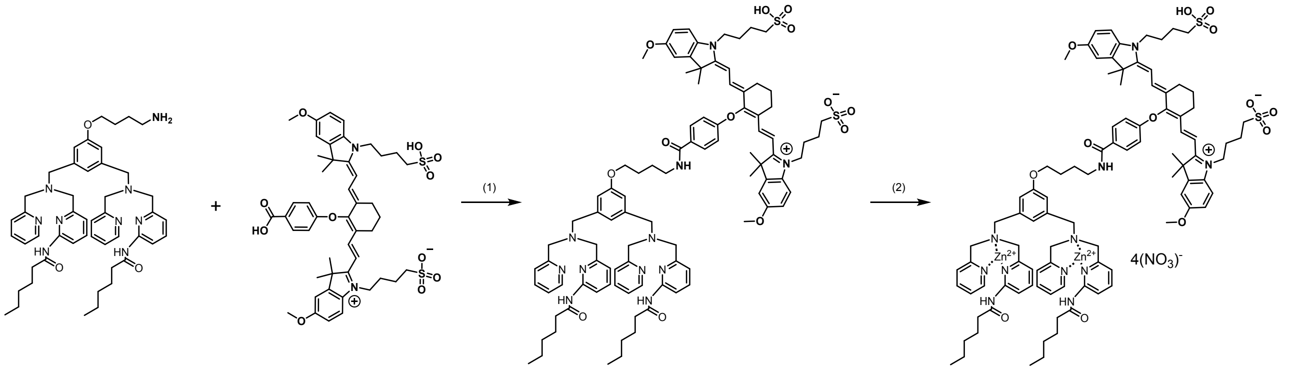

11 **794** **11-794** **Zn11-794**

Reagents and conditions: (1) EDCI, HOBt, DMF, dark, rt, 15 h.; (2) 2.0 equiv. $Zn(NO_3)_2$, MeOH.

Scheme S3. Synthetic procedures for conjugation between dye 794 and analog **11** to afford **Zn11-794**.

Methyl 6-(hexanoylamino)pyridine-2-carboxylate (2): To a solution of methyl 6-aminopicolinate (50.0 g, 329.0 mmol) in anhydrous CH_2Cl_2 (330 mL) at room temperature was added hexanoic acid (68.7 g, 592.2 mmol), followed by *N*-(3-dimethylaminopropyl)-*N'*-ethylcarbodiimide hydrochloride (EDCI, 113.5 g, 592.2 mmol) and lastly 4-(dimethylamino)pyridine (DMAP, 16.1 g, 131.6 mmol), then the reaction was allowed to stir at 40 °C. After stirring at 40 °C for 16 hours, the reaction mixture was added 2N $HCl_{(aq)}$ at room temperature and then adjusted to pH 2-3. The aqueous phase was extracted with CH_2Cl_2 (100 mL x 2). The combined organic layers were added saturated $Na_2CO_{3\ (aq)}$ (50 mL) at ice-bath temperature and then stirred for 1 hour, and the residue was extracted with CH_2Cl_2 (100 mL x 3) afterwards. The combined organic layers were dried over Na_2SO_4, filtered off and concentrated in vacuo. The crude product **2** was used for the next step without further purification. ^{1}H NMR (300 MHz, $CDCl_3$) δ 8.49 – 8.42 (m, 1H), 8.18 (br, 1H), 7.88 – 7.83 (m, 2H), 4.00 (s, 3H), 2.42 – 2.35 (m, 2H), 1.72 (d, *J* = 8.0 Hz, 2H), 1.35 (dq, *J* = 7.3, 3.5 Hz, 4H), 0.94 – 0.88 (m, 3H). ^{13}C NMR (151 MHz, $CDCl_3$) δ 172.2, 165.3, 151.5, 145.9, 139.5, 121.2, 117.8, 53.1, 37.9, 31.5, 25.1, 22.5, 14.0. HRMS (ESI): calc. for $C_{13}H_{18}N_2NaO_3^+$: 273.1210, found: 273.1207.

***N*-[6-(Hydroxymethyl)pyridin-2-yl]hexanamide (3):** To a solution of compound **2** (83.0 g, 332.0 mmol) in anhydrous MeOH (1105 mL) at ice-bath temperature was slowly added $NaBH_4$ (87.9 g, 2324.0 mmol), then the reaction was allowed to stir at room temperature for 4 hours. After the reaction was completed, the reaction mixture was quenched with saturated $NH_4Cl_{(aq)}$ at ice-bath temperature and then adjusted to pH 6-7, and stirred for 1.5 hours afterwards. Organic volatiles were evaporated, then the residue was partitioned into H_2O and CH_2Cl_2. The aqueous phase was extracted with CH_2Cl_2 (200 mL x 3), and the combined organic layers were dried over Na_2SO_4, filtered off and concentrated in vacuo. The crude product **3** was used for the next step without further purification. ^{1}H NMR (400 MHz, $CDCl_3$) δ 8.13 (d, *J* = 8.2 Hz, 1H), 8.04 (br, 1H), 7.69 (t, *J* = 7.9 Hz, 1H), 6.98 (d, *J* = 7.5 Hz, 1H), 4.68 (s, 2H), 2.41 (t, *J* = 7.6 Hz, 2H), 1.81 – 1.67 (m, 2H), 1.40 – 1.32 (m, 4H), 0.97 – 0.85 (m,

3H). ^{13}C NMR (151 MHz, $CDCl_3$) δ 172.0, 157.5, 150.8, 139.3, 116.2, 112.4, 64.0, 38.0, 31.5, 25.2, 22.5, 14.0. HRMS (ESI): calc. for $C_{12}H_{18}N_2NaO_2^+$: 245.1260, found: 245.1269.

***N*-(6-Formylpyridin-2-yl)hexanamide (4):** To a solution of compound **3** (68.0 g, 306.0 mmol) in CH_2Cl_2 (440 mL) at room temperature was added MnO_2 (106.4 g, 1224.0 mmol), and afterwards the reaction was allowed to stir at 40 °C for 15 hours. After the reaction was completed, the resulting mixture was purified by flash chromatography (celite: silica gel= 2: 1) with CH_2Cl_2 (1000 mL), followed by 20% EtOAc in Hexane to yield the product **4** (44.0 g, 70% in three steps). ^{1}H NMR (600 MHz, $CDCl_3$) δ 9.92 (d, J = 0.9 Hz, 1H), 8.49 (dd, J = 8.3, 1.0 Hz, 1H), 8.09 (br, 1H), 7.89 (tt, J = 7.5, 0.6 Hz, 1H), 7.68 (dd, J = 7.4, 0.9 Hz, 1H), 2.47 – 2.41 (m, 2H), 1.79 – 1.74 (m, 2H), 1.40 – 1.36 (m, 4H), 0.94 – 0.90 (m, 3H). ^{13}C NMR (151 MHz, $CDCl_3$) δ 192.4, 172.2, 152.0, 150.9, 139.6, 118.7, 118.6, 37.9, 31.5, 25.1, 22.5, 14.0. HRMS (ESI): calc. for $C_{12}H_{16}N_2NaO_2^+$: 243.1104, found: 243.1110.

***N*-(6-{[(Pyridin-2-ylmethyl)amino]methyl}pyridin-2-yl)hexanamide (5):** To a solution of compound **4** (44.0 g, 200.0 mmol) in anhydrous MeOH (400 mL) at room temperature was added 2-Picolylamine (19.5 g, 180.0 mmol), then stirred at room temperature for 3 hours. The reaction mixture was slowly added $NaBH_4$ (37.83 g, 1000.0 mmol) at ice-bath temperature, then the reaction was slowly warmed to room temperature and was stirred for 15 hours. After the reaction was completed, the residue was slowly quenched with saturated $NH_4Cl_{(aq)}$ at ice-bath temperature and then adjusted to pH 6-7, then stirred for 1.5 hours. Organic volatiles were evaporated, then the residue was partitioned into H_2O and CH_2Cl_2. The aqueous phase was extracted with CH_2Cl_2 (150 mL x 3), then the combined organic layers were dried over Na_2SO_4, filtered off and concentrated in vacuo. The crude product **5** was used for the next step without further purification (35 g, 90%). ^{1}H NMR (600 MHz, $CDCl_3$) δ 8.57 – 8.53 (m, 1H), 8.24 (br, 1H), 8.08 (d, J = 8.0 Hz, 1H), 7.65 – 7.60 (m, 2H), 7.31 (dd, J = 7.9, 3.4 Hz, 1H), 7.18 – 7.13 (m, 1H), 7.02 (dd, J = 7.5, 3.8 Hz, 1H), 3.95 (d, J = 3.2 Hz, 2H), 3.86 (d, J = 2.9 Hz,

2H), 2.82 (br, 1H), 2.39 – 2.34 (m, 2H), 1.76 – 1.68 (m, 2H), 1.39 – 1.31 (m, 4H), 0.94 – 0.87 (m, 3H). ^{13}C NMR (151 MHz, $CDCl_3$) δ 172.0, 159.6, 157.8, 151.2, 149.4, 138.8, 136.6, 122.5, 122.1, 118.1, 112.1, 54.7, 54.3, 37.8, 31.4, 25.1, 22.5, 14.0. HRMS (ESI): calc. for $C_{18}H_{25}N_4O^+$: 313.2023, found: 313.2026.

3,5-Bis(hydroxymethyl)phenol (7): To a stirred solution of **compound 6** (10.6 g, 50 mmol, 1 eq) in 500 ml of dry THF at 0 ℃, $LiAlH_4$ (7.5 g, 0.2 mol, 4 eq) was slowly added. The resultant reaction mixture was stirred at 50 ℃ for 15 hours. After the solution was cooled to 0 ℃, ammonium chloride aqueous solution (50 ml) was slowly added. The mixture was stirred at 0 ℃ for 1 hour. The mixture was filtered with celite, washed with THF. The resultant residue was concentrated under reduced pressure to yield **compound 7** (5 g, 64%). ^{1}H NMR (400 MHz, MeOD) δ 6.82 – 6.80 (m, 1H), 6.72 – 6.70 (m, 2H), 4.90 (br, 3H), 4.53 (s, 4H). ^{13}C NMR (151 MHz, MeOD) δ 158.6, 144.3, 117.6, 113.6, 65.1. LCMS (ESI): calc. for $C_8H_9O_3^-$: 153.05, found: 153.11.

2-{4-[3,5-Bis(hydroxymethyl)phenoxy]butyl}-1*H*-isoindole-1,3(2*H*)-dione (8): To a stirred solution of **compound 7** (7.8 g, 50.6 mmol, 1 eq) in ACN (200 ml) and DMF (30 ml) at room temperature, 2-(4-bromobutyl)isoindoline-1,3-dione (21 g, 76 mmol, 1.5 eq) and K_2CO_3 (34g, 0.25 mol, 5 eq) were slowly added. The resultant reaction mixture was stirred for 1 hour, heated at 40 ℃ for 14 hours. After removal of MeOH and ACN, the resultant residue was extracted with CH_2Cl_2 (200 mL). The CH_2Cl_2 solution was then washed with water (200 mL), dried over $MgSO_4$, and concentrated under reduced pressure. The resultant residue was purified by column chromatography (silica gel; EA : Hexane = 1 : 1) to yield **compound 8** (8 g, 44%). ^{1}H NMR (400 MHz, $CDCl_3$) δ 7.82 (dd, J = 5.4, 3.1 Hz, 2H), 7.71 (dd, J = 5.5, 3.0 Hz, 2H), 6.87 (d, J = 1.7 Hz, 1H), 6.76 (d, J = 1.5 Hz, 2H), 4.61 – 4.53 (m, 4H), 3.97 (t, J = 5.8 Hz, 2H), 3.73 (t, J = 6.7 Hz, 2H), 2.81 (br, 2H), 1.90 – 1.75 (m, 4H). ^{13}C

NMR (151 MHz, $CDCl_3$) δ 168.7, 159.4, 142.9, 134.1, 132.1, 123.4, 117.6, 112.2, 67.3, 65.1, 37.7, 26.6, 25.3. LCMS (ESI): calc. for $C_{20}H_{21}NNaO_5^+$: 378.13, found: 378.26.

5-[4-(1,3-Dioxo-1,3-dihydro-2*H*-isoindol-2-yl)butoxy]benzene-1,3-dicarbaldehyde (9): To a stirred solution of **compound 8** (500 mg, 1.41 mmol, 1 eq) in 100 mL of dry CH_2Cl_2 at room temperature, MnO_2 (1.84 g, 21.1 mmol, 15 eq) was slowly added. The resultant reaction mixture was stirred at room temperature for 15 hours. The mixture was filtered with celite, washed with CH_2Cl_2. The resultant residue was concentrated under reduced pressure to yield **compound 9** (395 mg, 80%). ^1H NMR (400 MHz, $CDCl_3$) δ 10.04 (s, 2H), 7.94 (t, *J* = 1.4 Hz, 1H), 7.85 (dd, *J* = 5.4, 3.1 Hz, 2H), 7.72 (dd, *J* = 5.4, 3.1 Hz, 2H), 7.62 (d, *J* = 1.3 Hz, 2H), 4.16 – 4.08 (m, 2H), 3.78 (t, *J* = 3.7 Hz, 2H), 1.94 – 1.88 (m, 4H). ^{13}C NMR (151 MHz, $CDCl_3$) δ 191.0, 168.5, 160.2, 138.4, 134.1, 132.2, 124.1, 123.4, 120.1, 68.2, 37.6, 26.5, 25.3. LCMS (ESI): calc. for $C_{20}H_{18}NO_5^+$: 352.12, found: 352.10.

***N*,*N*'-([5-(4-aminobutoxy)benzene-1,3-diyl]bis{methanediyl[(pyridin-2-ylmethyl)imino]methanediylpyridine-6,2-diyl})dihexanamide (11):** To a solution of compound **9** (10.3 g, 29.4 mmol) in CH_2Cl_2 (120 mL) at room temperature was added compound **5** (23.0 g, 73.5 mmol), followed by $NaB(OAc)_3H$ (28.1 g, 132.3 mmol). The reaction mixture was stirred at room temperature for 18 hours. After the reaction was completed, the resulting mixture was poured into saturated $NaHCO_{3(aq)}$ (60 mL) at ice-bath temperature. The aqueous later was extracted with CH_2Cl_2 (100 mL x 3). The combined organic layers were dried over Na_2SO_4, filtered off and concentrated in vacuo. The crude product **10** was obtained, which was used for next reaction without further purification.

The crude product **10** (7.6 g, 8.1 mmol) in EtOH (30 mL) was added hydrazine (1 mL, 32.2 mmol) at room temperature, then stirred for 15 hours. The reaction was monitored by LC. After the reaction was

completed, the residues were washed with EtOH and filtered off. The organic layers were concentrated in vacuo, then the residue was purified by reversed-phase chromatography (90% MeOH in H_2O) to yield the compound **11** (4.0 g, 61%, in two steps).

^{1}H NMR (600 MHz, $CDCl_3$) δ 8.89 (s, 2H, CH-Py.), 8.49 (tt, *J* = 3.1, 1.7 Hz, 2H, CH-Py.), 8.11 (d, *J* = 8.2 Hz, 2H, CH-Py.), 7.72 – 7.61 (m, 2H, CH-Py.), 7.56 (tt, *J* = 7.6, 1.6 Hz, 2H, CH-Py.), 7.48 (dd, *J* = 7.7, 1.4 Hz, 2H, CH-Py.), 7.23 (s, 1H, CH-Ph.), 7.22 (d, *J* = 5.4 Hz, 2H), 7.13 (ddd, *J* = 7.8, 3.9, 2.6 Hz, 2H, CH-Py.), 6.72 (q, *J* = 1.5 Hz, 2H, CH-Ph.), 3.94 (td, *J* = 6.3, 2.7 Hz, 2H, CH_2-alkyl), 3.77 (d, *J* = 2.3 Hz, 4H, CH_2-αPh.), 3.68 (s, 4H, CH_2-αPh.), 3.57 (s, 4H, CH_2-αPh.), 2.82 (td, *J* = 6.4, 1.8 Hz, 2H, CH_2-alkyl), 2.11 (td, *J* = 7.7, 3.0 Hz, 4H, CH_2-alkyl), 1.87 – 1.76 (m, 2H, CH_2-alkyl), 1.68 (p, *J* = 7.3 Hz, 2H, CH_2-alkyl), 1.56 (p, *J* = 7.6 Hz, 4H, CH_2-alkyl), 1.30 – 1.12 (m, 8H, CH_2-alkyl), 0.91 – 0.76 (m, 6H, CH_3-alkyl).

^{13}C NMR (151 MHz, $CDCl_3$) δ 172.3, 159.7, 159.0, 157.9, 151.5, 149.0, 140.1, 139.1, 136.6, 123.1, 122.3, 122.3, 119.1, 114.0, 112.6, 67.7, 60.3, 59.6, 58.3, 41.6, 37.5, 31.4, 29.4, 26.7, 25.1, 22.4, 14.0.

HRMS (ESI): calc. for $C_{48}H_{63}N_9NaO_3^+$: 836.4946, found: 836.4952.

3-(Pyridin-2-yldisulfaneyl)propanoic acid (13): To a solution of compound **12** (2.0 g, 18.8 mmol) in MeOH (13 mL) was added 2,2'-Dipyridyl disulfide (6.2 g, 28.3 mmol) and the reaction mixture was stirred at room temperature for 15 hours. Organic volatiles were evaporated, then the residue was purified by flash chromatography with 50% EtOAc/hexane to yield the product **13** (3.0 g, 74%). ^{1}H NMR (600 MHz, MeOD) δ 8.40 (ddt, *J* = 4.9, 1.7, 0.8 Hz, 1H), 7.85 (dq, *J* = 8.2, 1.0 Hz, 1H), 7.81 (dddd, *J* = 8.1, 7.3, 1.8, 0.7 Hz, 1H), 7.23 (ddt, *J* = 7.2, 4.9, 1.0 Hz, 1H), 3.04 (td, *J* = 6.9, 0.7 Hz, 2H), 2.71 (td, *J* = 6.9, 0.7 Hz, 2H). LCMS (ESI): calc. for $C_8H_{10}NO_2S_2^+$:216.01, found: 216.11.

3-[(3-{[(2*S*)-1-{[(1*S*,2*R*,3*S*,5*S*,6*S*,16*E*,18*E*,20*R*,21*S*)-11-chloro-21-hydroxy-12,20-dimethoxy-2,5,9,16-tetramethyl-8,23-dioxo-4,24-dioxa-9,22-diazatetracyclo[19.3.1.1^{10,14}.0^{3,5}]hexacosa-

10(26),11,13,16,18-pentaen-6-yl]oxy}-1-oxopropan-2-yl](methyl)amino}-3-oxopropyl)disulfanyl]propanoic acid (15): To a solution of compound **13** (0.3 g, 1.5 mmol) in CH_2Cl_2 (22 mL) was added compound **14** (DM-1, 1.0 g, 1.4 mmol) and the reaction mixture was stirred at 35 ℃ for 16 hours. The reaction was monitored by LC. After the reaction was completed, iced ether (220mL) was added dropwise into the residue at 4 ℃ to precipitate the crude **15**. The precipitate was filtered off and washed with iced ether (50 mL x 3) to give the product **15** (0.95 g, 85%). ^{1}H NMR (600 MHz, DMSO-d_6) δ 7.17 (d, *J* = 1.8 Hz, 1H), 6.90 (d, *J* = 1.2 Hz, 1H), 6.65 – 6.49 (m, 2H), 5.94 (s, 1H), 5.56 (dd, *J* = 14.7, 9.0 Hz, 1H), 5.31 (q, *J* = 6.8 Hz, 1H), 4.52 (dd, *J* = 12.1, 2.8 Hz, 1H), 4.06 (ddd, *J* = 12.4, 10.6, 2.1 Hz, 1H), 3.92 (s, 3H, CH_3-alkyl), 3.56 – 3.43 (m, 2H), 3.25 (s, 3H, CH_3-alkyl), 3.20 (d, *J* = 12.5 Hz, 1H), 3.13 (s, 3H, CH_3-alkyl), 2.96 – 2.76 (m, 4H), 2.72 (s, 3H, CH_3-alkyl), 2.66 (t, *J* = 7.1 Hz, 2H), 2.62 – 2.55 (m, 1H), 2.46 (td, *J* = 7.0, 2.9 Hz, 2H), 2.04 (dd, *J* = 14.5, 2.8 Hz, 1H), 1.59 (s, 3H, CH_3-alkyl), 1.51 – 1.38 (m, 2H), 1.31 – 1.21 (m, 3H), 1.17 (d, *J* = 6.8 Hz, 3H, CH_3-alkyl), 1.12 (d, *J* = 6.4 Hz, 3H, CH_3-alkyl), 0.89 – 0.80 (m, 2H), 0.78 (s, 3H, CH_3-alkyl). LCMS (ESI): calc. for $C_{38}H_{51}ClN_3O_{12}S_2^-$: 840.26, found: 840.20.

3-[(5-{[(2*S*)-1-{[(1*S*,2*R*,3*S*,5*S*,6*S*,16*E*,18*E*,20*R*,21*S*)-11-chloro-21-hydroxy-12,20-dimethoxy-2,5,9,16-tetramethyl-8,23-dioxo-4,24-dioxa-9,22-diazatetracyclo[19.3.1.1^{10,14}.0^{3,5}]hexacosa-10(26),11,13,16,18-pentaen-6-yl]oxy}-1-oxopropan-2-yl](methyl)amino}-2-methyl-5-oxopentan-2-yl)disulfanyl]propanoic acid (17): To a solution of compound **13** (27.60 mg, 0.12 mmol) in MeOH (5 mL) and potassium phosphate buffer (50 mM, pH 7.5, 3.56 mL), compound **16** (DM4, 0.05g, 0.06 mmol) was added. The reaction mixture was stirred at room temperature for 15 hours. The solvent was then removed in vacuo. The product was purified by flash chromatography over silica gel with Methanol/CH_2Cl_2 (3/97) to give **17** as a white solid (40 mg, 71%). ^{1}H NMR (400 MHz, $CDCl_3$): δ 6.83 (d, *J* = 1.2 Hz,1H), 6.67 (d, *J* = 10.8 Hz,1H), 6.64 (d, *J* = 1.2 Hz, 1H), 6.48 (s, 1H), 6.42 (dd, *J* = 15.6, 9.2 Hz, 1H), 5.67 (dd, *J* = 15.2, 9.2Hz, 1H), 5.31 (br, 1H), 4.80 (dd, *J* = 12.0, 3.2 Hz, 1H), 4.29(t, *J* =

12.0 Hz, 1H), 3.99 (s, 3H), 3.64 (d, *J* = 12.8 Hz, 1H), 3.49 (d, *J* = 9.2 Hz, 1H), 3.36 (s, 3H), 3.22 (s, 3H), 3.12 (d, *J* = 12.8Hz, 1H), 3.01 (d, *J* = 9.6 Hz, 1H) , 2.87 (s, 3H), 2.86 (t, *J* = 7.2Hz, 2H), 2.71-2.57 (m, 3H), 2.53-2.42 (m, 1H), 2.40-2.29 (m, 1H), 2.19 (dd, *J* = 14.4, 2.8Hz, 1H), 2.09-1.81 (m, 2H), 1.64 (s, 3H) , 1.60 (d, *J* = 13.6 Hz, 1H), 1.53-1.39 (m, 1H), 1.3 (t, *J* = 7.2 Hz, 6H), 1.25 (s, 6H) 1.23-1.20 (m, 1H), 0.88 (t, *J* = 6.8Hz, 1H), 0.81 (s, 3H). LCMS (ESI): calc. for $C_{41}H_{57}ClN_3O_{12}S_2^-$: 882.30, found:882.30.

(1*S*,2*R*,3*S*,5*S*,6*S*,16*E*,18*E*,20*R*,21*S*)-11-chloro-21-hydroxy-12,20-dimethoxy-2,5,9,16-tetramethyl-8,23-dioxo-4,24-dioxa-9,22-diazatetracyclo[19.3.1.1^{10,14}.0^{3,5}]hexacosa-10(26),11,13,16,18-pentaen-6-yl (2*S*)-2-(methyl{3-[(5-nitropyridin-2-yl)disulfanyl]propanoyl}amino)propanoate (18): To a solution of compound **14** (DM-1, 0.2 g, 0.27 mmol) in DMF (9 mL) was added a solution of 2,2'-dithiobis(5-nitropyridine) (0.19 g, 0.62 mmol) in THF (19 mL). N-Methylmorpholine (NMM, 1.43 mmol, 0.16 mL) was then added to the stirred solution. The resultant reaction solution was stirred at room temperature for 15 hours, poured onto saturated $NaHCO_{3(aq.)}$, and extracted twice with ethyl acetate. The combined organic extracts were dried over Na_2SO_4, filtered, and concentrated in vacuo. The product was purified by flash chromatography over silica gel with methanol/dichloromethane (3/97) to give **18** as a white solid (0.20 g, 83%). ^{1}H NMR (600 MHz, MeOD) δ 9.05 (dd, *J* = 2.6, 0.7 Hz, 1H), 8.41 (dd, *J* = 8.9, 2.7 Hz, 1H), 7.88 (dd, *J* = 8.9, 0.7 Hz, 1H), 6.91 (d, *J* = 1.8 Hz, 1H), 6.67 (dt, *J* = 11.2, 1.3 Hz, 1H), 6.57 (dd, *J* = 15.3, 11.2 Hz, 1H), 6.46 (d, *J* = 1.7 Hz, 1H), 5.70 (dd, *J* = 15.2, 9.1 Hz, 1H), 5.46 (q, *J* = 6.8 Hz, 1H), 4.65 (dd, *J* = 12.1, 2.9 Hz, 1H), 4.58 (s, 5H), 4.17 (td, *J* = 10.4, 4.2 Hz, 1H), 3.56 (d, *J* = 9.0 Hz, 1H), 3.46 (d, *J* = 12.5 Hz, 1H), 3.37 (s, 3H, CH_3-alkyl), 3.21 (s, 3H, CH_3-alkyl), 3.09 – 3.05 (m, 2H), 2.94 – 2.91 (m, 1H), 2.86 (d, *J* = 0.7 Hz, 1H), 2.84 (s, 3H, CH_3-alkyl), 2.55 (dd, *J* = 14.5, 12.1 Hz, 1H), 2.09 (dd, *J* = 14.5, 2.9 Hz, 1H), 2.06 – 1.98 (m, 1H), 1.63 (s, 3H, CH_3-alkyl), 1.49 – 1.46 (m, 2H), 1.32 – 1.26 (m, 8H), 1.22 (d, *J* = 6.4 Hz, 3H, CH_3-alkyl), 0.93 – 0.88 (m, 1H), 0.80 (s, 3H, CH_3-alkyl).

LCMS(ESI) calculated for $C_{40}H_{50}ClNaN_5O_{12}S_2^+$: 914.24, found: 914.53(M +Na$^+$)$^+$

4-[(3-{[(2*S*)-1-{[(1*S*,2*R*,3*S*,5*S*,6*S*,16*E*,18*E*,20*R*,21*S*)-11-chloro-21-hydroxy-12,20-dimethoxy-2,5,9,16-tetramethyl-8,23-dioxo-4,24-dioxa-9,22-diazatetracyclo[19.3.1.1^{10,14}.0^{3,5}]hexacosa-10(26),11,13,16,18-pentaen-6-yl]oxy}-1-oxopropan-2-yl](methyl)amino}-3-oxopropyl)disulfanyl]-4-methylpentanoic acid (19): The compound **18** (0.20 g, 0.22 mmol) in DMF (3.36 mL) was added 4-Mercapto-4-methylpentanoic acid (49.80 mg, 0.34 mmol) in THF (0.75 mL) and potassium phosphate buffer (50 mM, pH 7.5, 2.80 mL). The reaction solution was stirred at room temperature for 15 hours. The solvent was then removed in vacuo. The product was purified by flash chromatography over silica gel with Methanol/CH_2Cl_2 (3/97) to give **19** as a white solid (57.00 mg, 29%). ^{1}H NMR (400 MHz, $CDCl_3$): δ 6.83 (s,1H), 6.68-6.61 (m, 2H), 6.46-6.38 (m, 2H), 6.466.38 (m, 2H), 5.64 (dd, *J* = 15.6, 8.8 Hz, 1H), 5.27 (br, 1H), 4.81 (dd, *J* = 12.4, 3.2 Hz, 1H), 4.30(t, *J* = 12.0 Hz, 1H), 3.98 (s, 3H, CH_3-alkyl), 3.65 (d, *J* = 12.8 Hz, 1H), 3.49 (d, *J* = 9.2 Hz, 1H), 3.36 (s, 3H, CH_3-alkyl) , 3.24 (s, 3H, CH_3-alkyl), 3.11 (d, *J* = 13.2Hz, 1H), 3.00 (d, *J* = 9.2 Hz, 1H) , 2.97-2.9 (m, 1H), 2.89 (s, 3H, CH_3-alkyl), 2.88-2.73 (m, 2H), 2.70-2.57 (m, 3H, CH_3-alkyl), 2.40-2.32 (m, 2H), 2.19 (dd, *J* = 14.4, 3.2Hz, 1H), 1.92-1.82 (m, 2H), 1.65 (s, 3H, CH_3-alkyl) , 1.60 (d, *J* = 13.6 Hz, 1H), 1.51-1.40 (m, 1H), 1.36-1.24 (m, 6H), 1.21 (d, *J* = 2.8Hz, 6H), 0.91-0.82 (m, 1H), 0.81 (s, 3H, CH_3-alkyl). LCMS (ESI): calc. for $C_{41}H_{57}ClN_3O_{12}S_2^-$: 882.31, found: 882.10(M-H$^+$)$^-$.

4-(pyridin-2-yldisulfaneyl)benzoic acid (21): To a solution of 2,2'-dipyridyl disulfide (350 mg, 1.58 mmol) in MeOH (10ml) was added 4-mercaptobenzoic acid (300 mg, 1.94 mmol, 1.2 eq.) and the reaction mixture was stirred at room temperature for 3 hours. Organic volatiles were evaporated, then the residue was purified by flash chromatography with 5% MeOH/ CH_2Cl_2 to yield **the compound 21** (100 mg, 0.38 mmol, 24%) ^{1}H NMR (600 MHz, MeOD) δ 7.71 – 7.63 (m, 2H), 7.55 – 7.40 (m, 4H), 6.97 – 6.79 (m, 2H). LCMS (ESI): calc. for $C_{12}H_{10}NO_2S_2^+$: 264.01, found: 264.11

1-(4-{[(tert-butoxycarbonyl)amino]methyl}phenyl)-3-oxo-5,8,11-trioxa-2-azatridecan-13-oic acid (23): To a solution of compound **22** (5.0 g, 21.2 mmol) in anhydrous CH_2Cl_2 (140 mL) at ice-bath temperature was added 3,6,9-trioxaundecanedioic acid (14.1 g, 63.0 mmol), then a solution of EDCI (6.1 g, 31.7 mmol) in CH_2Cl_2 (70 mL) was slowly added into the reaction mixture, and afterwards the reaction mixture was slowly warmed to room temperature. After stirring at room temperature for 18 hours, saturated $NH_4Cl_{(aq)}$ was poured into the reaction mixture and then adjusted to pH 6-7. The aqueous phase was extracted with CH_2Cl_2 (200 mL x 3), and organic volatiles were evaporated. The residue was partitioned into saturated $NaHCO_{3(aq)}$ and EtOAc, then the organic phase was extracted with saturated $NaHCO_{3(aq)}$ (50 mL x 2). After phase was separated, the aqueous layers were added 2N $HCl_{(aq)}$ at ice-bath temperature and then adjusted to pH 2-3, the reaction mixture was extracted with CH_2Cl_2 (150 mL x 2). The combined organic layers were washed with H_2O, dried over Na_2SO_4, filtered off and concentrated in vacuo. The crude product **23** was used for the next step without further purification. ^{1}H NMR (400 MHz, MeOD) δ 7.29 – 7.21 (m, 4H), 4.43 (s, 2H), 4.20 (s, 2H), 4.04 (s, 2H), 4.02 – 4.00 (m, 2H), 3.71 – 3.64 (m, 4H), 3.61 (s, 4H), 1.45 (s, 9H). ^{13}C NMR (151 MHz, MeOD) δ 174.1, 172.8, 158.6, 140.0, 138.6, 128.7, 128.4, 80.2, 72.1, 71.6, 71.4, 71.3, 71.2, 69.1, 44.7, 43.3, 28.8. HRMS (ESI): calc. for $C_{21}H_{33}N_2O_8^+$: 441.2231, found: 441.2224.

4-(2-{(*E*)-2-[(3*E*)-2-(4-{[4-(3,5-bis{[{[6-(hexanoylamino)pyridin-2-yl]methyl}(pyridin-2-ylmethyl)amino]methyl}phenoxy)butyl]carbamoyl}phenoxy)-3-{(2*E*)-2-[5-methoxy-3,3-dimethyl-1-(4-sulfobutyl)-1,3-dihydro-2*H*-indol-2-ylidene]ethylidene}cyclohex-1-en-1-yl]ethenyl}-5-methoxy-3,3-dimethyl-3*H*-indolium-1-yl)butane-1-sulfonate (11-794): To a stirred solution of compound 11 (35 mg, 0.043 mmol, 1.2 equiv) was dissolved in DMF (3 ml), and **4-(2-{(*E*)-2-[(3*E*)-2-(4-carboxyphenoxy)-3-{(2*E*)-2-[5-methoxy-3,3-dimethyl-1-(4-sulfobutyl)-1,3-dihydro-2*H*-indol-2-ylidene]ethylidene}cyclohex-1-en-1-yl]ethenyl}-5-methoxy-3,3-dimethyl-3*H*-indolium-1-yl)butane-1-sulfonate (compound 794)** (30 mg, 0.034 mmol, 1 equiv) and

hydroxybenzotriazole (HOBt, 10 mg, 0.074 mmol, 2.1 equiv) and *N*-(3-Dimethylaminopropyl)-*N′*-ethylcarbodiimide hydrochloride (EDCl, 10 mg, 0.064 mmol, 2 equiv) were added. The mixture was stirred at room temperature in the dark for 2 hours. After completion of reaction, the mixture was slowly dripped into the ether (100 ml) subsequent unpurified compound 11-794 precipitated out. Purification of the crude residue by reversed-phase chromatography on Lichroprep@RP-18 was eluting with methanol gradient grade to get **11-794** (23 mg, 0.014 mmol, 41%). ^{1}H NMR (400 MHz, CD_3OD) δ 8.39 (d, J = 4.8 Hz, 2H), 7.97 – 7.88 (m, 4H), 7.83 (d, J = 14.1 Hz, 2H), 7.78 – 7.69 (m, 3H), 7.69 – 7.58 (m, 5H), 7.28 – 7.16 (m, 10H), 7.02 (s, 1H), 6.94 (t, J = 1.7 Hz, 2H), 6.92 – 6.85 (m, 2H), 6.77 (s, 2H), 6.12 (d, J = 14.2 Hz, 1H), 4.09 (s, 4H), 3.97 (s, 3H), 3.61 (dd, J = 7.0, 1.1 Hz, 6H), 3.58 (d, J = 1.3 Hz, 7H), 3.43 (d, J = 7.0 Hz, 4H), 2.94 – 2.79 (m, 6H), 2.74 (s, 5H), 2.34 (t, J = 7.6 Hz, 5H), 2.03 (s, 3H), 1.96 – 1.73 (m, 18H), 1.65 (t, J = 7.4 Hz, 8H), 1.18 (td, J = 7.1, 1.1 Hz, 9H), 0.97 – 0.78 (m, 10H). HRMS (ESI), calc. for $C_{95}H_{118}N_{11}O_{13}S_2^+$: 1685.8380, found: 1685.8366 $(M + H^+)^+$.

4-(2-{(*E*)-2-[(3*E*)-2-(4-{[4-(3,5-bis{[{[6-(hexanoylamino)pyridin-2-yl]methyl}(pyridin-2-ylmethyl)amino]methyl}phenoxy)butyl]carbamoyl}phenoxy)-3-{(2*E*)-2-[5-methoxy-3,3-dimethyl-1-(4-sulfobutyl)-1,3-dihydro-2*H*-indol-2-ylidene]ethylidene}cyclohex-1-en-1-yl]ethenyl}-5-methoxy-3,3-dimethyl-3*H*-indolium-1-yl)butane-1-sulfonate * 2[$Zn(NO_3)_2$] (Zn11-794)

To a stirred solution of compound 11-794 (23 mg, 0.014 mmol) in 2 mL MeOH was added 1 mL of $Zn(NO_3)_2$ (8.12 mg, 0.028 mmol) in MeOH at room temperature. After 10 min, the mixture was concentrated under reduced pressure to get 28 mg (Zn11-794, 0.013 mmol, 93%). ^{1}H NMR (600 MHz, MeOD) δ 8.59 (ddd, *J* = 5.3, 1.6, 0.9 Hz, 2H), 8.05 – 8.01 (m, 2H), 8.00 – 7.95 (m, 2H), 7.95 – 7.88 (m, 2H), 7.65 – 7.58 (m, 2H), 7.57 – 7.50 (m, 2H), 7.31 – 7.26 (m, 2H), 7.25 – 7.15 (m, 5H), 7.07 – 7.02 (m, 2H), 6.96 (s, 4H), 6.85 (dd, *J* = 8.7, 2.4 Hz, 2H), 4.44 – 4.32 (m, 5H), 4.15 – 4.07 (m, 5H),

4.04 (dd, J = 13.6, 5.6 Hz, 2H), 3.87 (ddd, J = 16.5, 4.4, 2.5 Hz, 5H), 3.80 – 3.66 (m, 6H), 3.48 (t, J = 6.3 Hz, 2H), 2.84 (t, J = 7.2 Hz, 4H), 2.72 (qd, J = 7.6, 1.6 Hz, 5H), 1.98 – 1.77 (m, 20H), 1.48 – 1.37 (m, 10H), 1.29 (s, 16H), 0.99 – 0.92 (m, 7H). HRMS (ESI), calc. for $C_{95}H_{118}N_{11}O_{13}S_2Zn^{2+}$: 874.3794, found: 874.3795(M + Zn^{2+})$^{2+}$.

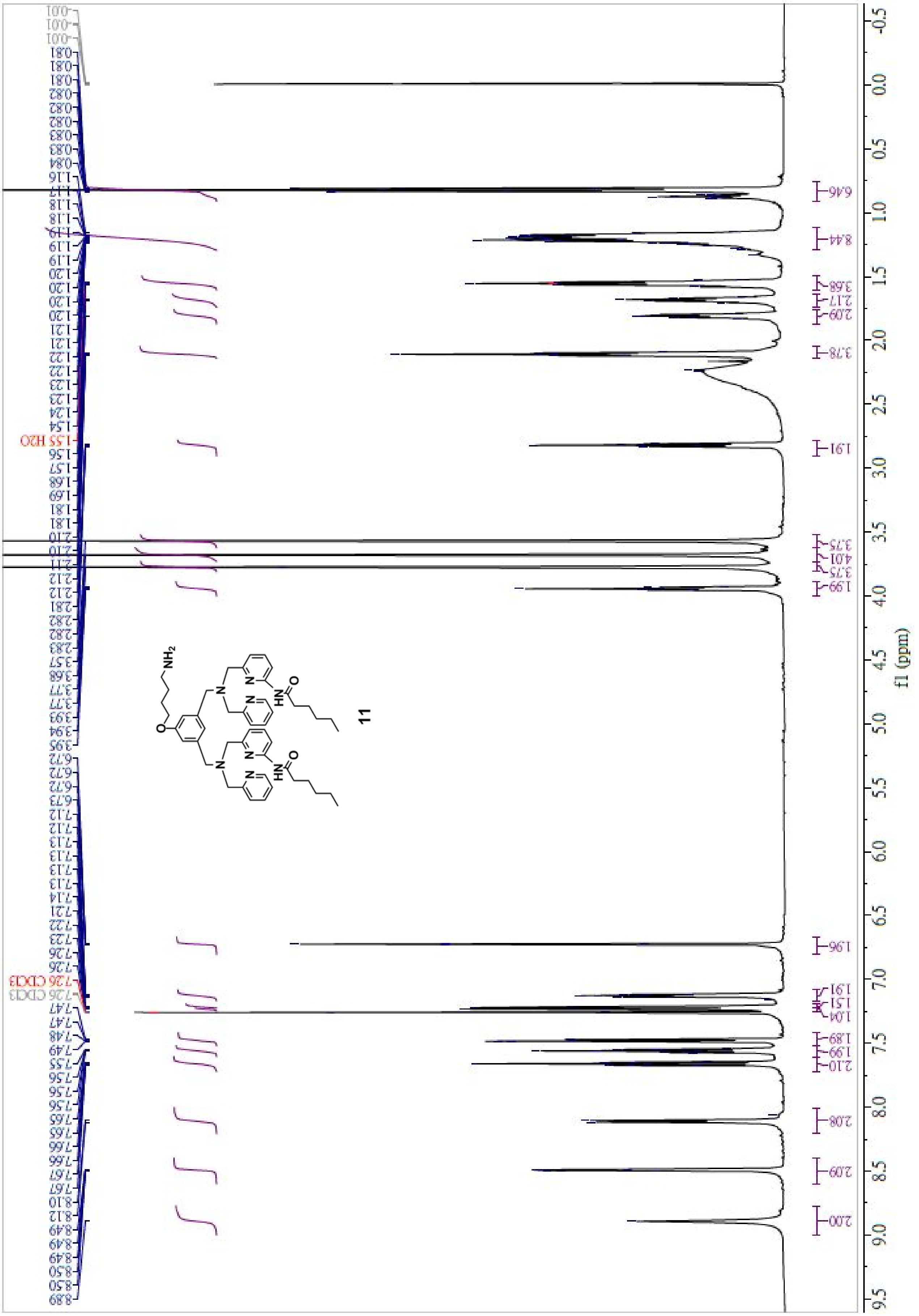
11
f1 (ppm)
1.55 H2O
7.26 CDCl3

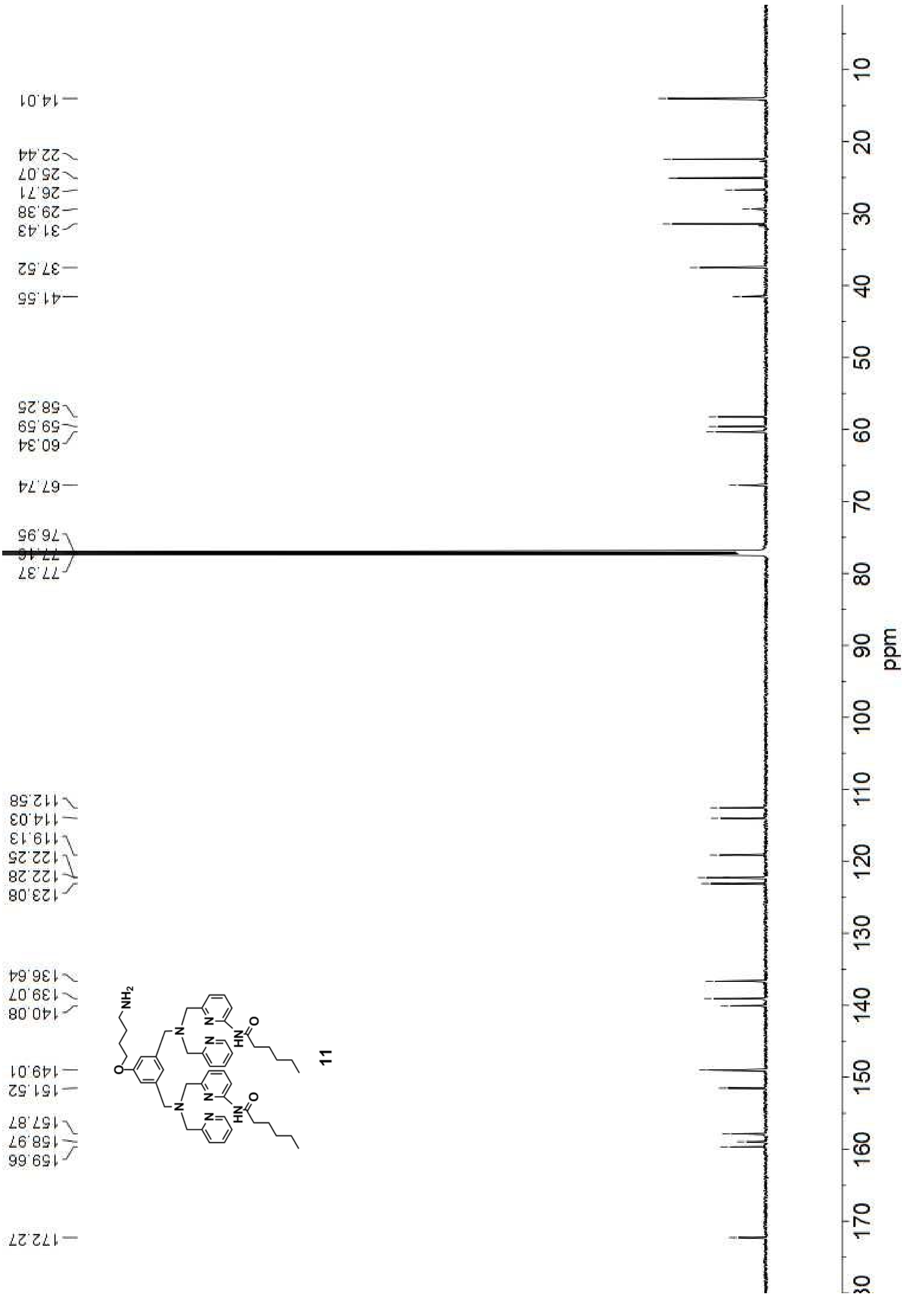
14.01
22.44
25.07
26.71
29.38
31.43
37.52
41.55
58.25
59.59
60.34
67.74
76.95
77.16
77.37
112.58
114.03
119.13
122.25
122.28
123.08
136.64
139.07
140.08
149.01
151.52
157.87
158.97
159.66
172.27
NH_2
O
N
HN
11
ppm
10
20
30
40
50
60
70
80
90
100
110
120
130
140
150
160
170

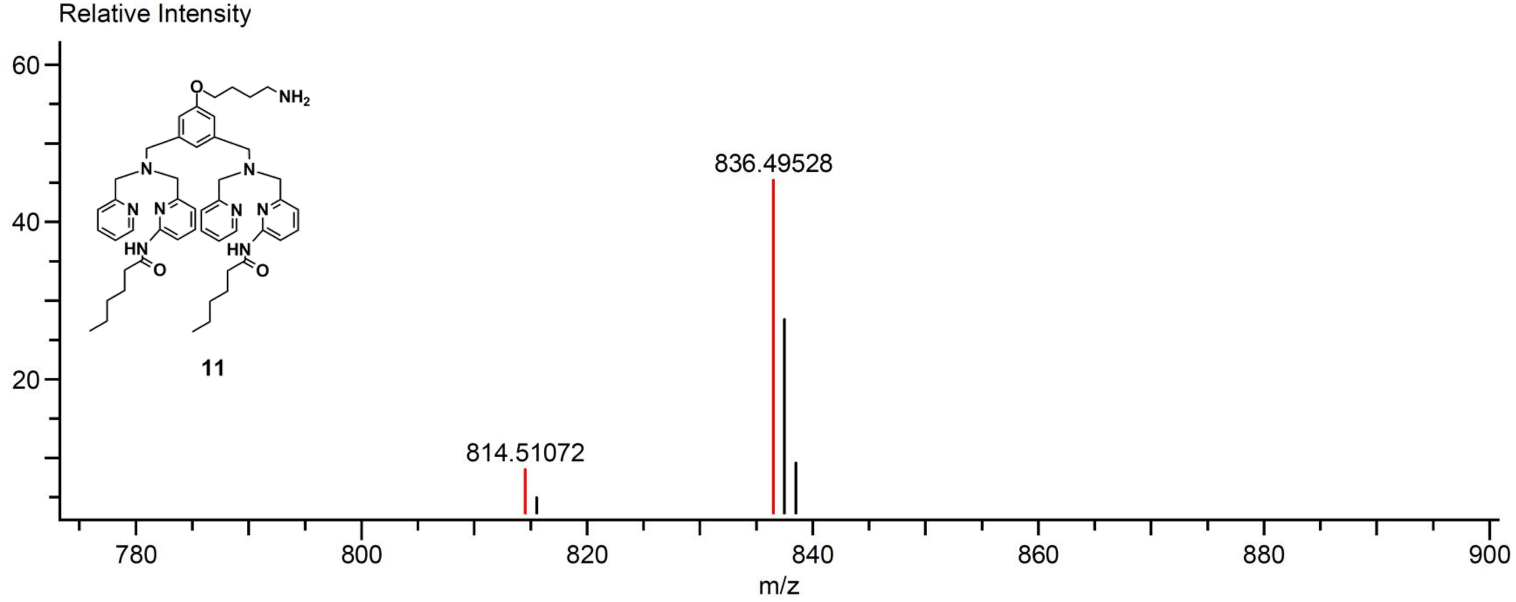

Mass	Intensity	Calc. Mass	Mass Difference [mDa]	Mass Difference [ppm]	Possible Formula
814.51072	2203.37	814.51321	-2.49	-3.06	$^{12}C_{48}{}^{1}H_{64}{}^{14}N_{9}{}^{16}O_{3}$
836.49528	11796.87	836.49515	0.13	0.15	$^{12}C_{48}{}^{1}H_{63}{}^{14}N_{9}{}^{23}Na_{1}{}^{16}O_{3}$

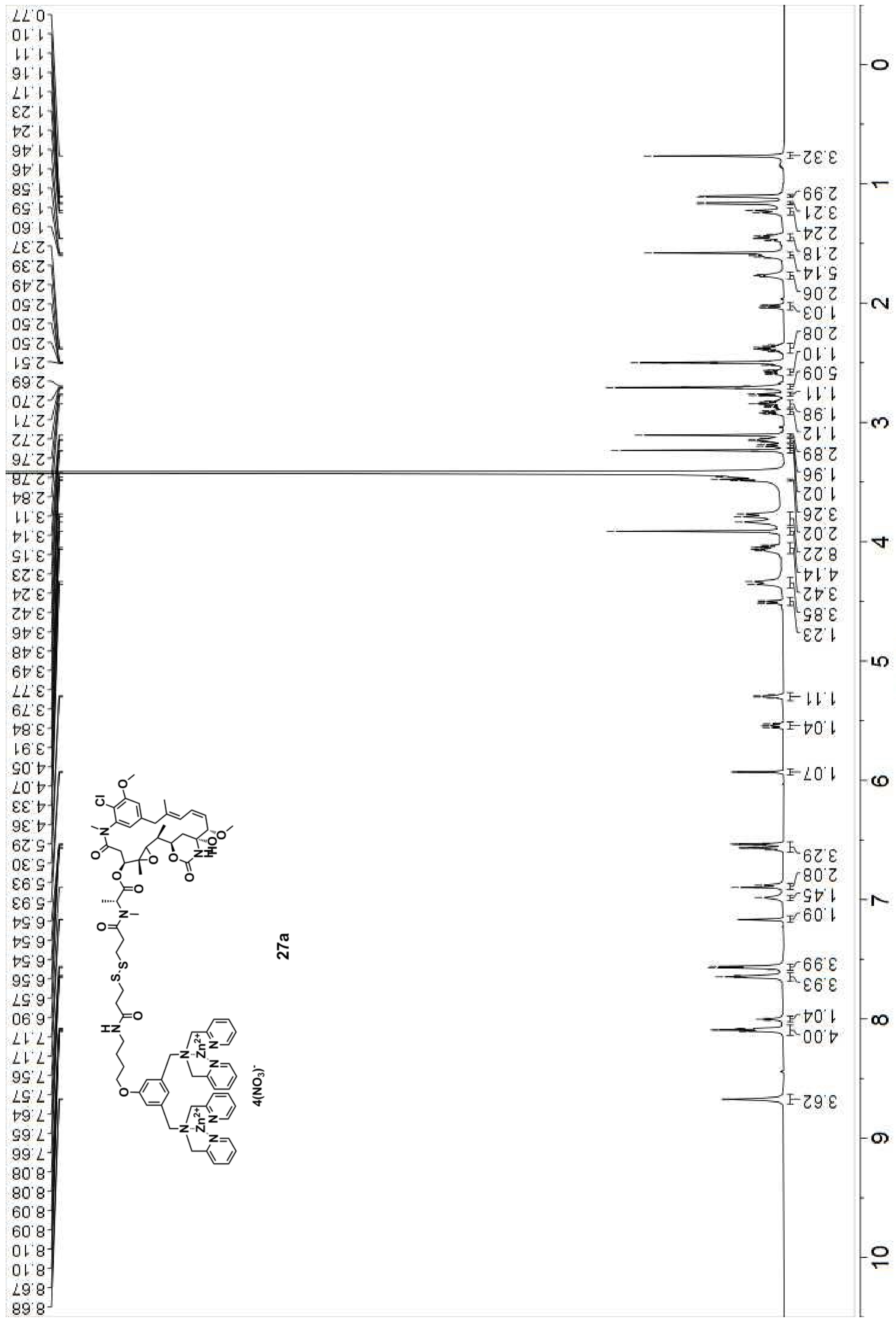
27a
ppm

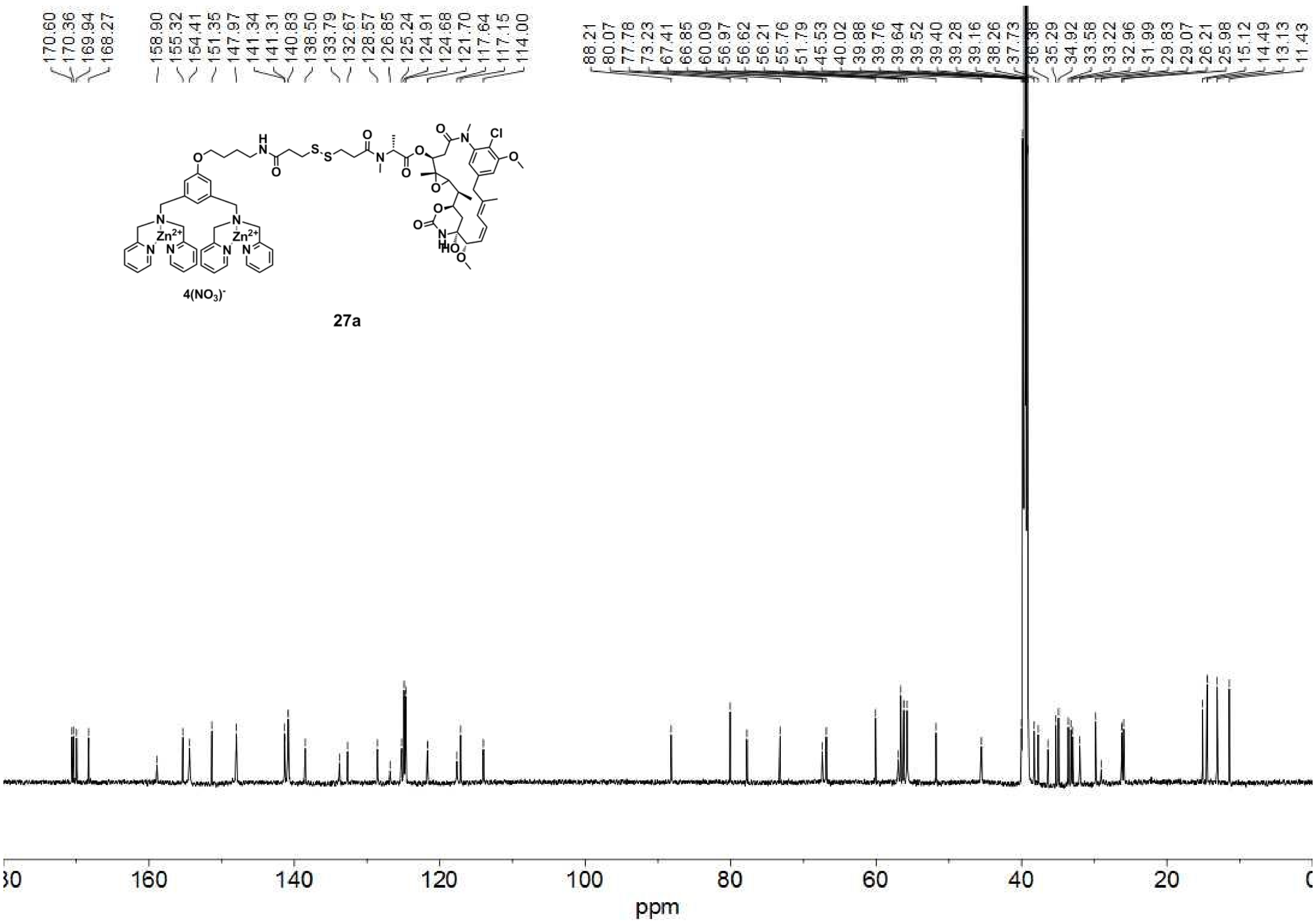
170.60
170.36
169.94
168.27
158.90
155.32
154.41
151.35
147.97
141.34
141.31
140.83
138.50
133.79
132.67
128.57
126.85
125.24
124.91
124.68
121.70
117.64
117.15
114.00
88.21
80.07
77.78
73.23
67.41
66.85
60.09
56.97
56.62
56.21
55.76
51.79
45.53
40.02
39.88
39.76
39.64
39.52
39.40
39.28
39.16
38.26
37.73
36.38
35.29
34.92
33.58
33.22
32.96
31.99
29.83
29.07
26.21
25.98
15.12
14.49
13.13
11.43
4(NO3)-
27a
160
140
120
100
80
60
40
20
ppm

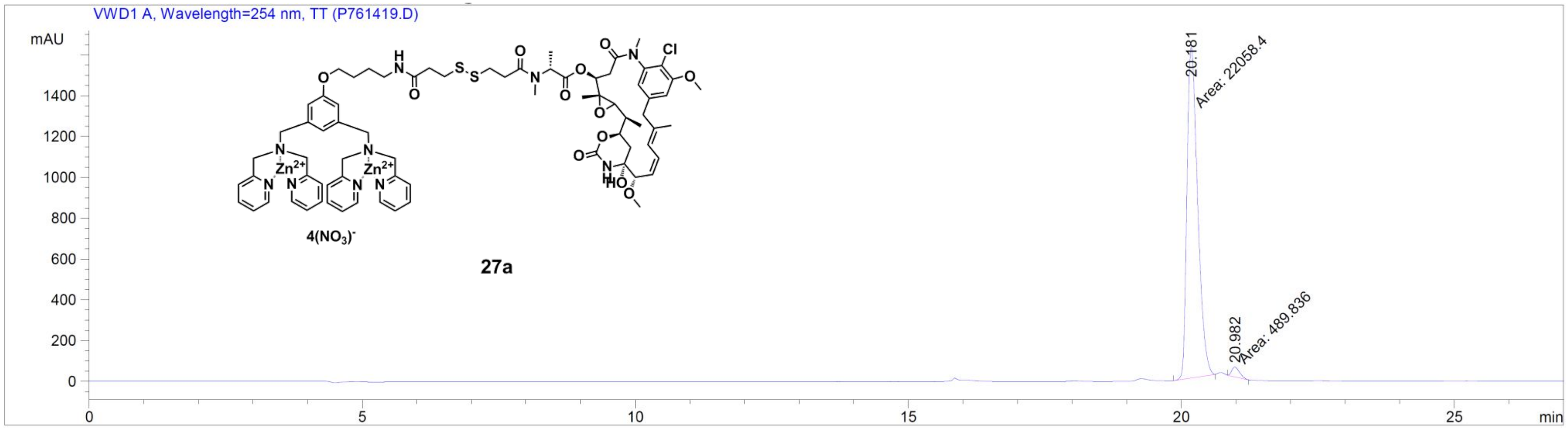

===
Area Percent Report
===

Sorted By : Signal
Multiplier : 1.0000
Dilution : 1.0000
Use Multiplier & Dilution Factor with ISTDs

Signal 1: VWD1 A, Wavelength=254 nm, TT

Peak #	RetTime [min]	Type	Width [min]	Area mAU *s	Height [mAU]	Area %
1	20.181	MM	0.2267	2.20584e4	1621.52478	97.8276
2	20.982	MM	0.1689	489.83585	48.32505	2.1724

Totals : 2.25482e4 1669.84983

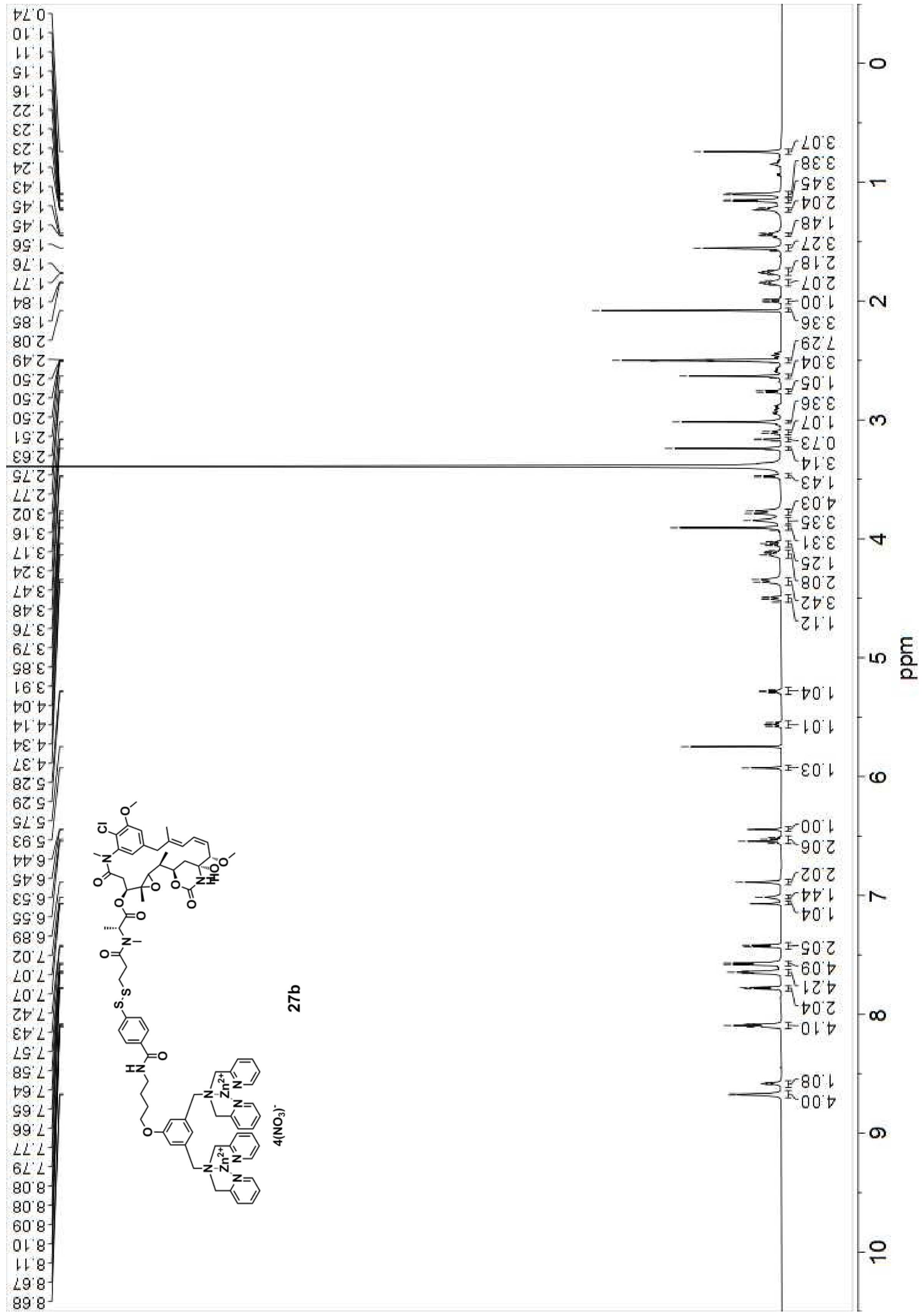
27b
4(NO3)-
ppm

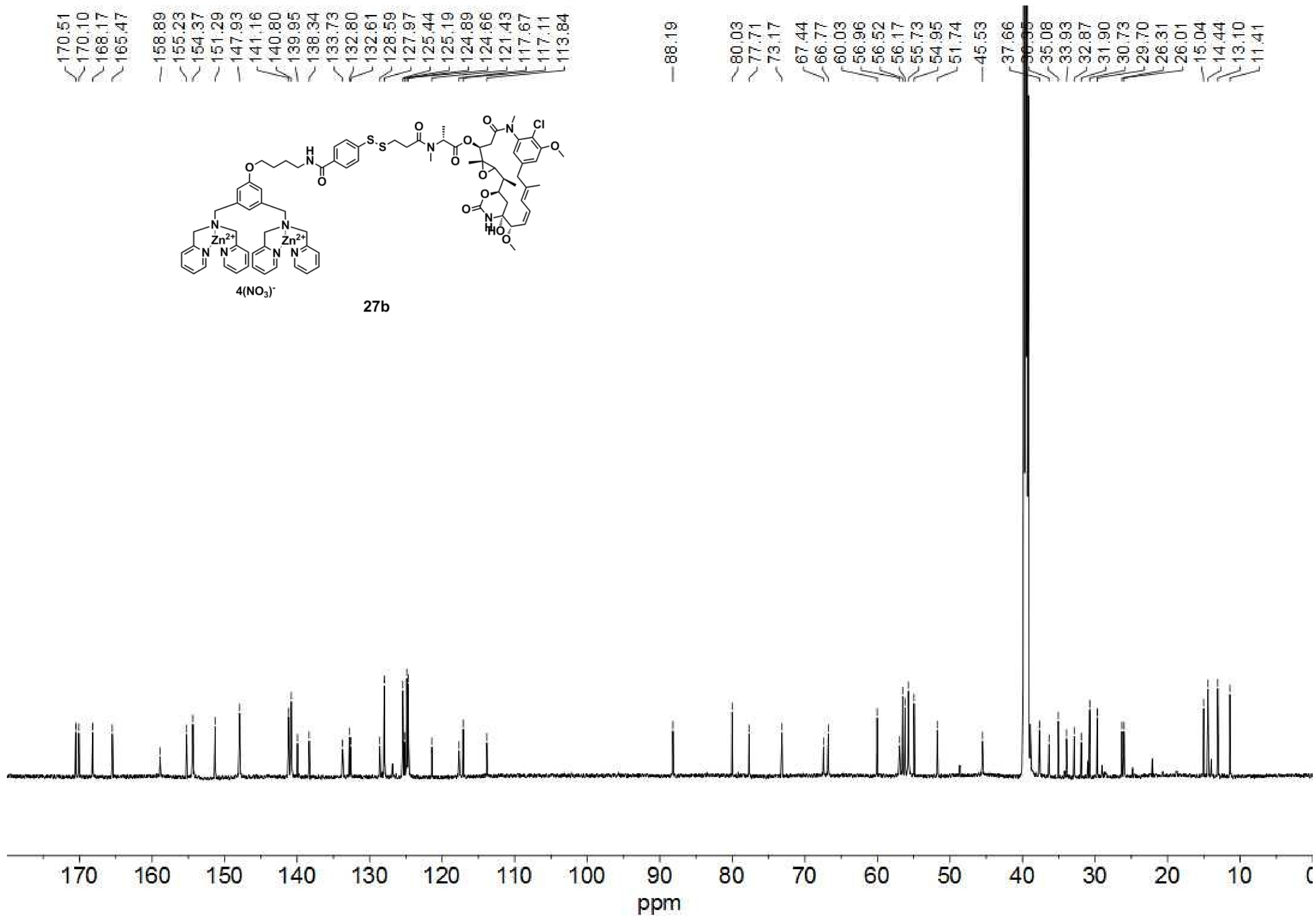
170.51
170.10
168.17
165.47
158.89
155.23
154.37
151.29
147.93
141.16
140.80
139.95
138.34
133.73
132.80
132.61
128.59
127.97
125.44
125.19
124.89
124.66
121.43
117.67
117.11
113.84
88.19
80.03
77.71
73.17
67.44
66.77
60.03
56.96
56.52
56.17
55.73
54.95
51.74
45.53
37.66
35.08
33.93
32.87
31.90
30.73
29.70
26.31
26.01
15.04
14.44
13.10
11.41
4(NO3)-
27b
170
160
150
140
130
120
110
100
90
80
70
60
50
40
30
20
10
ppm

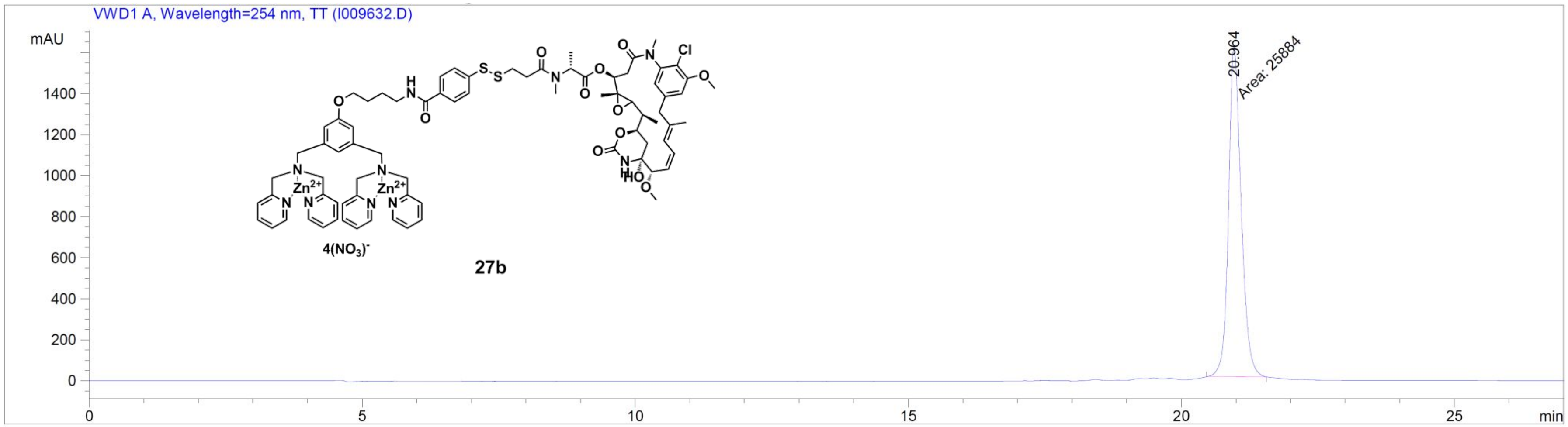


```
=====================================================================
                          Area Percent Report
=====================================================================

Sorted By             :      Signal
Multiplier            :      1.0000
Dilution              :      1.0000
Use Multiplier & Dilution Factor with ISTDs


Signal 1: VWD1 A, Wavelength=254 nm, TT

Peak RetTime Type  Width     Area      Height     Area
  #   [min]        [min]  mAU   *s   [mAU   ]      %
----|-------|----|-------|----------|----------|--------|
   1  20.964 MM    0.2685 2.58840e4  1606.40967 100.0000

Totals :                  2.58840e4  1606.40967
```

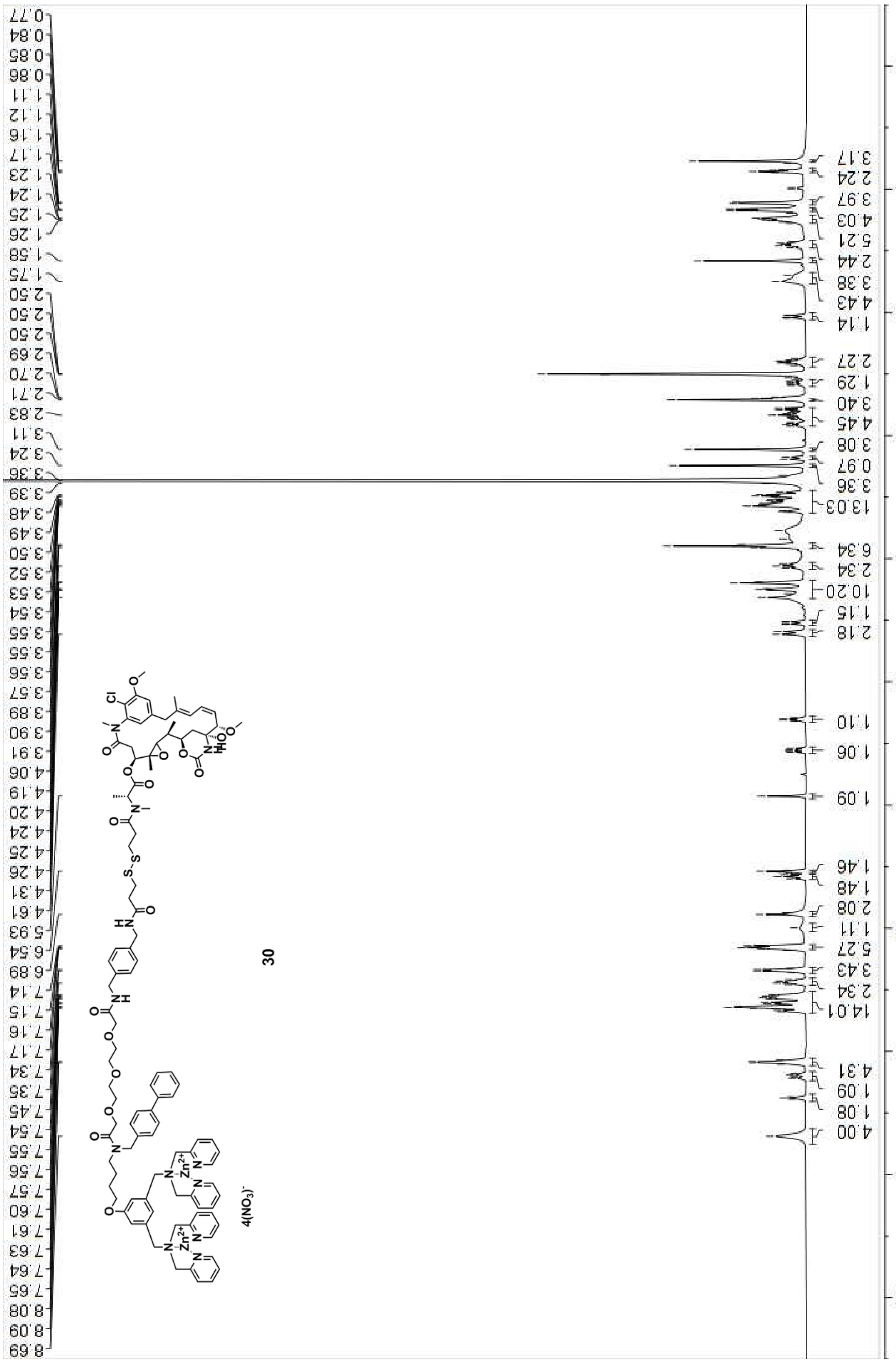
ppm
30

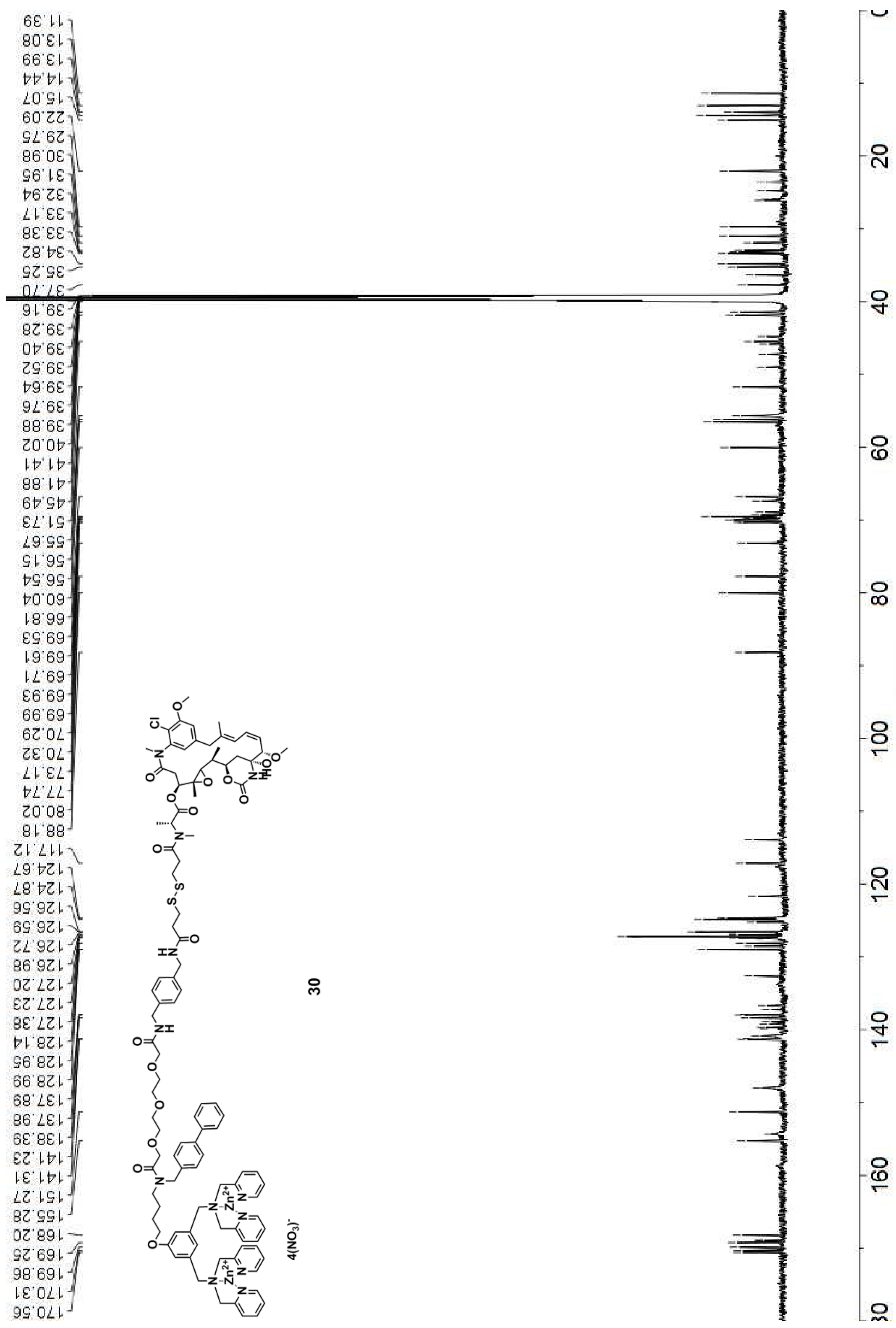
170.56
170.31
169.86
169.25
168.20
155.28
151.27
141.31
141.23
138.39
137.98
137.89
128.99
128.95
128.14
127.38
127.23
127.20
126.98
126.72
126.59
126.56
124.87
124.67
117.12
88.18
80.02
77.74
73.17
70.32
70.29
69.99
69.93
69.71
69.61
69.53
66.81
60.04
56.54
56.15
55.67
51.73
45.49
41.88
41.41
40.02
39.88
39.76
39.64
39.52
39.40
39.28
39.16
37.70
35.25
34.82
33.38
33.17
32.94
31.95
30.98
29.75
22.09
15.07
14.44
13.99
13.08
11.39
4(NO3)-
180
160
140
120
100
80
60
40
20
0
ppm

30

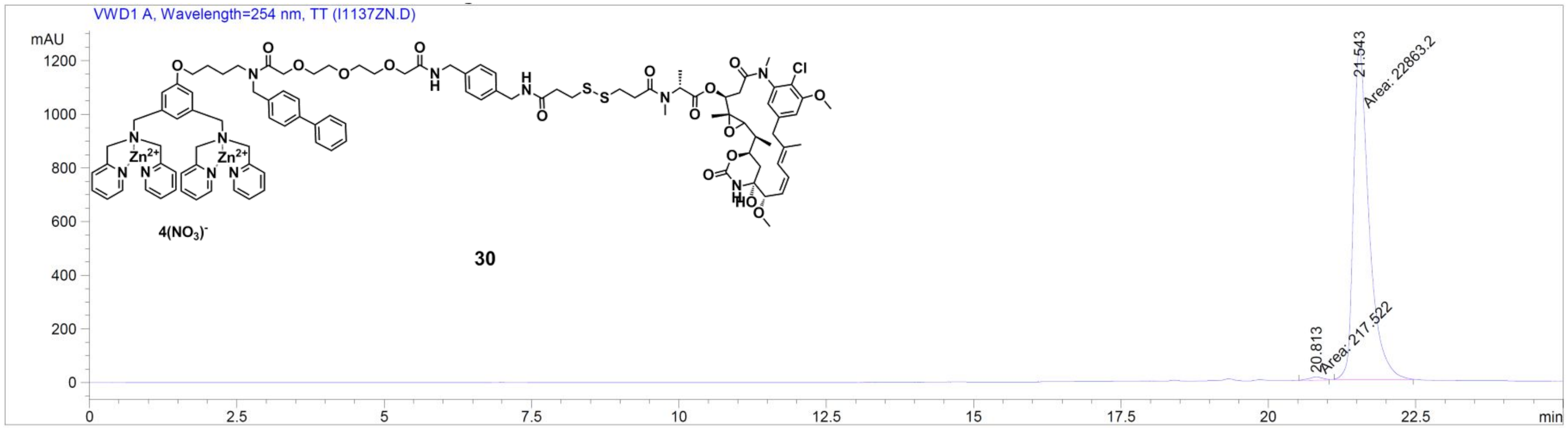


```
==================================================================
                         Area Percent Report
==================================================================

Sorted By             :      Signal
Multiplier            :      1.0000
Dilution              :      1.0000
Use Multiplier & Dilution Factor with ISTDs


Signal 1: VWD1 A, Wavelength=254 nm, TT

Peak RetTime Type  Width     Area      Height     Area
  #   [min]        [min]  mAU   *s   [mAU   ]       %
----|-------|----|-------|----------|----------|--------|
   1  20.813 MM    0.2836  217.52158   12.78129   0.9424
   2  21.543 MM    0.3076 2.28632e4  1238.79126  99.0576

Totals :                  2.30807e4  1251.57255
```

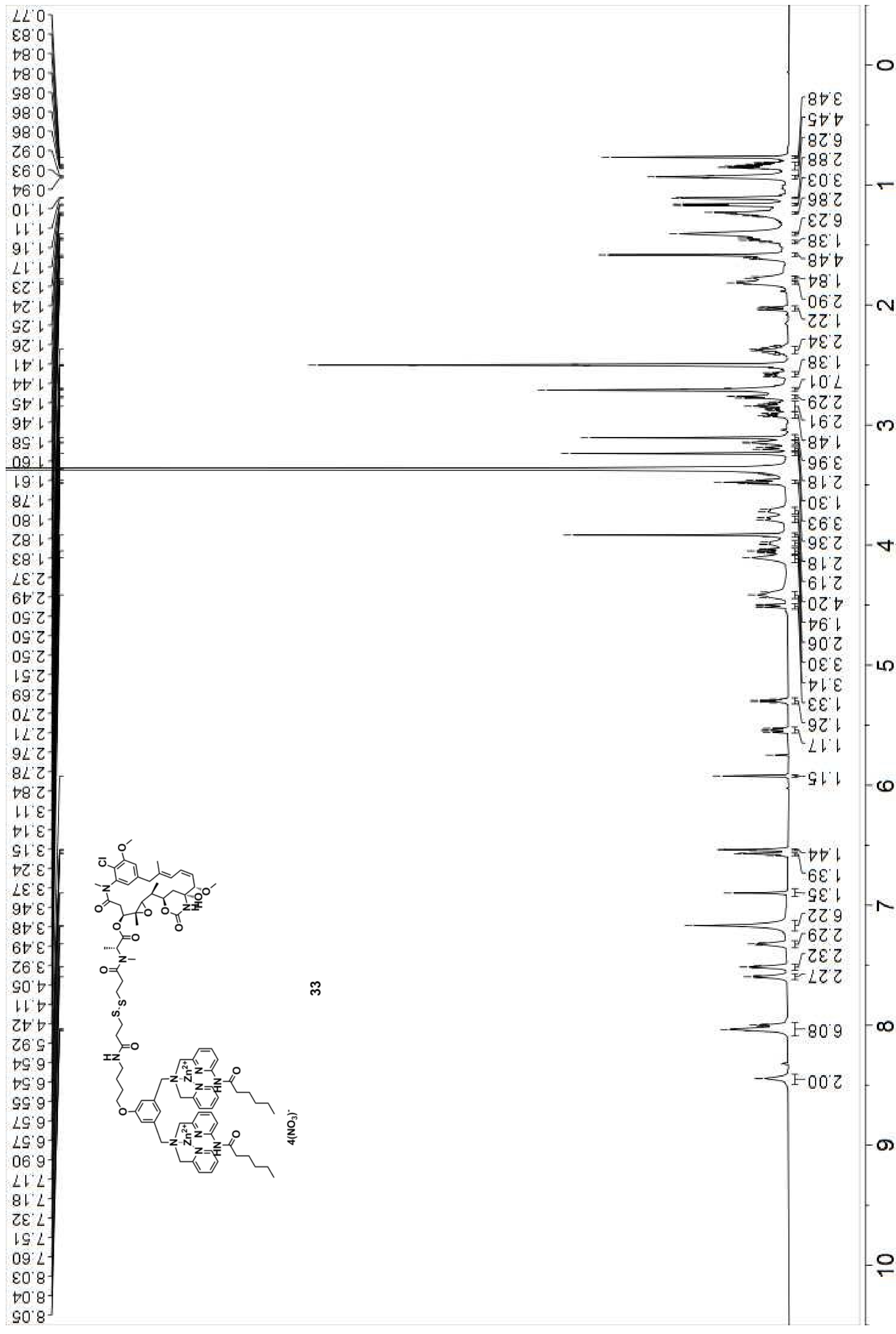
33
ppm

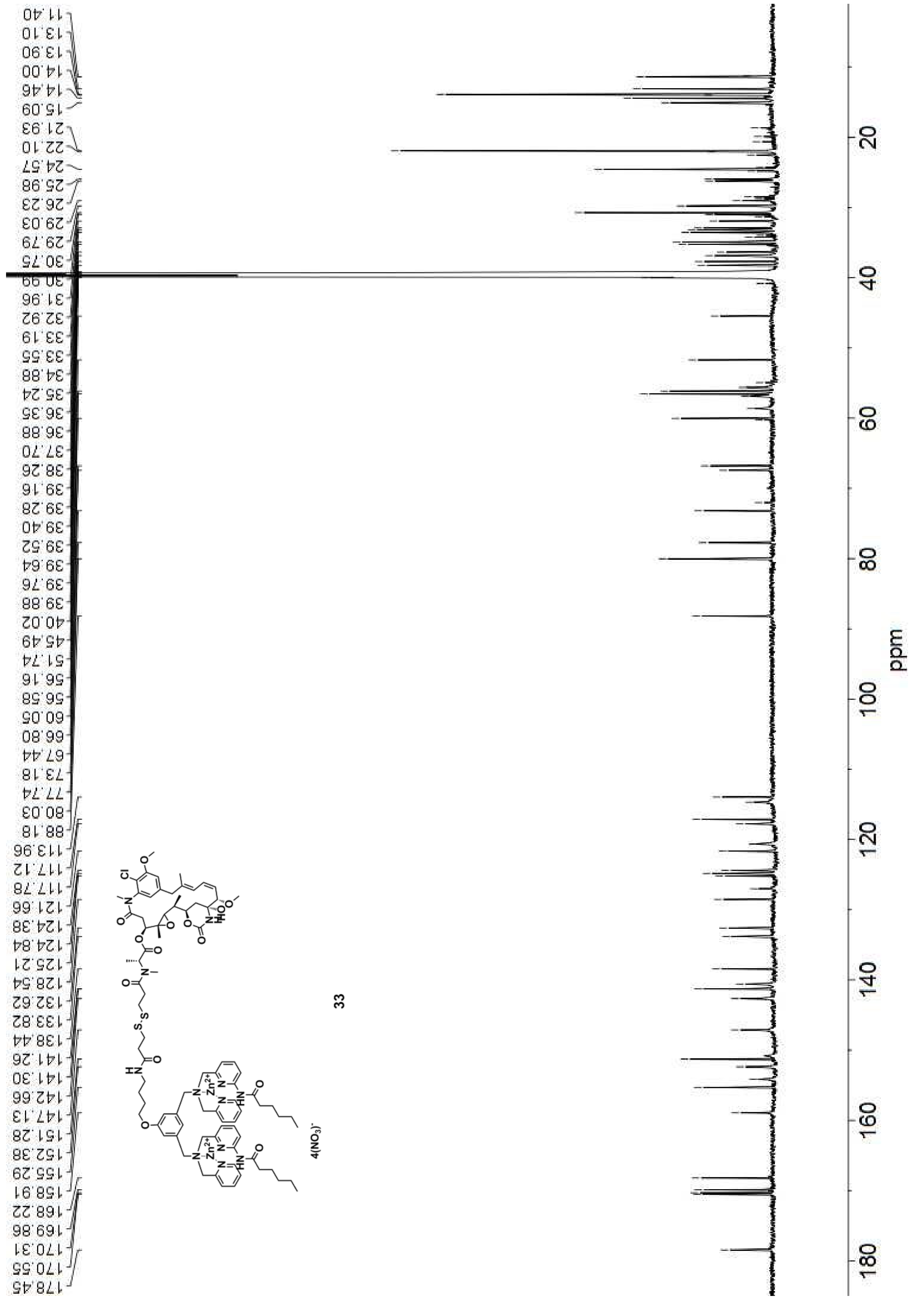
11.40
13.10
13.90
14.00
14.46
15.09
21.93
22.10
24.57
25.98
26.23
29.03
29.79
30.75
30.99
31.96
32.92
33.19
33.55
34.88
35.24
36.35
36.88
37.70
38.26
39.16
39.28
39.40
39.52
39.64
39.76
39.88
40.02
45.49
51.74
56.16
56.58
60.05
66.80
67.44
73.18
77.74
80.03
88.18
113.96
117.12
117.78
121.66
124.38
124.84
125.21
128.54
132.62
133.82
138.44
141.26
141.30
142.66
147.13
151.28
152.38
155.29
158.91
168.22
169.86
170.31
170.55
178.45
33
ppm
20
40
60
80
100
120
140
160
180

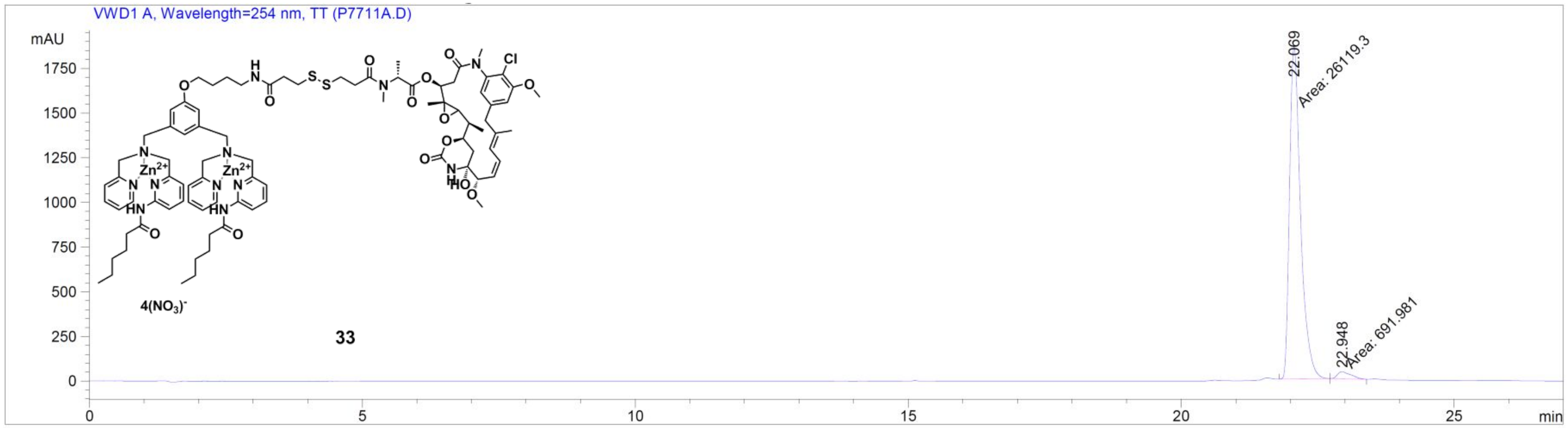

==

Area Percent Report

==

Sorted By : Signal
Multiplier : 1.0000
Dilution : 1.0000
Use Multiplier & Dilution Factor with ISTDs

Signal 1: VWD1 A, Wavelength=254 nm, TT

Peak #	RetTime [min]	Type	Width [min]	Area mAU *s	Height [mAU]	Area %
1	22.069	MM	0.2342	2.61193e4	1858.54565	97.4191
2	22.948	MM	0.2871	691.98083	40.17679	2.5809

Totals : 2.68113e4 1898.72245

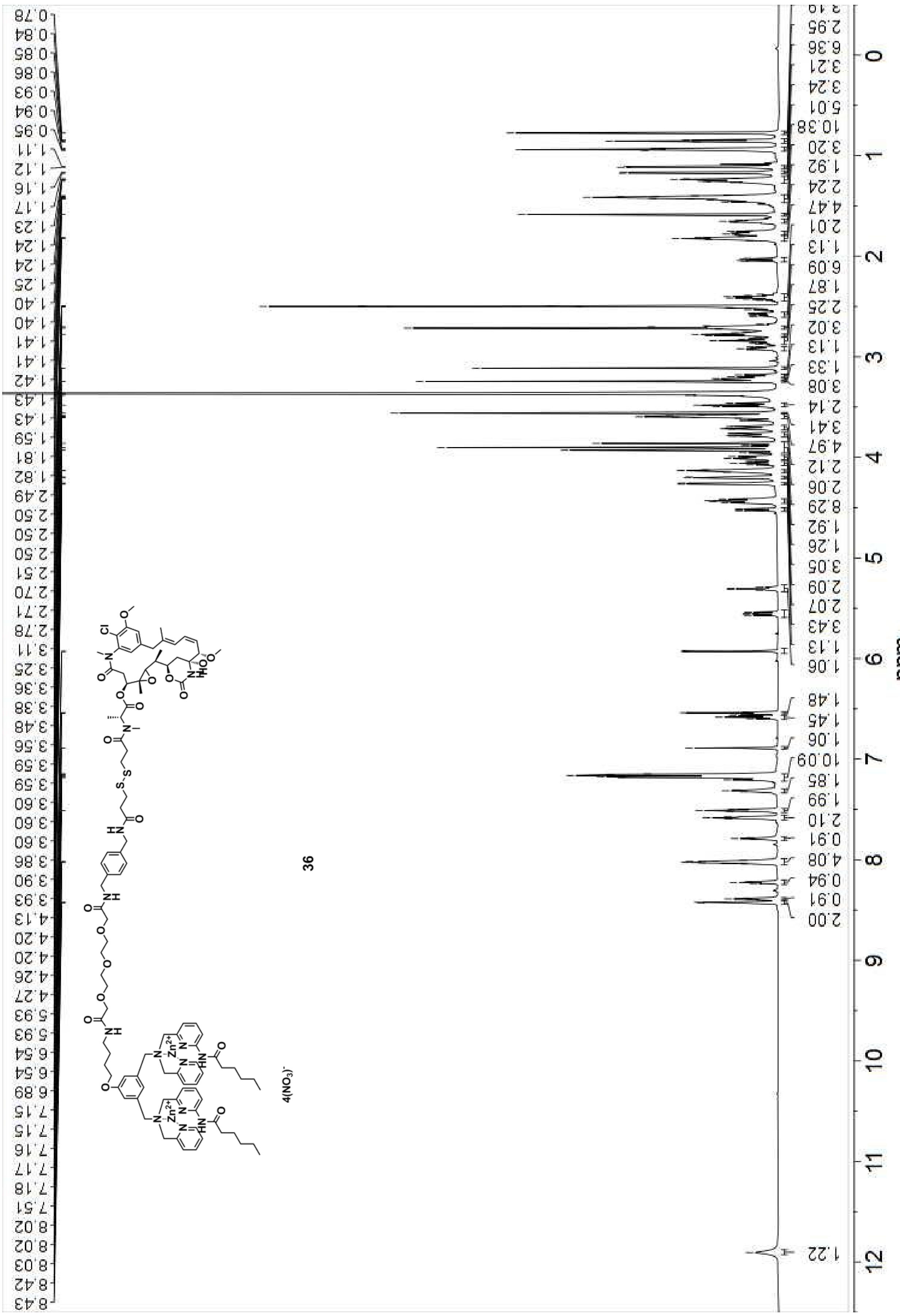
36
ppm
0
1
2
3
4
5
6
7
8
9
10
11
12

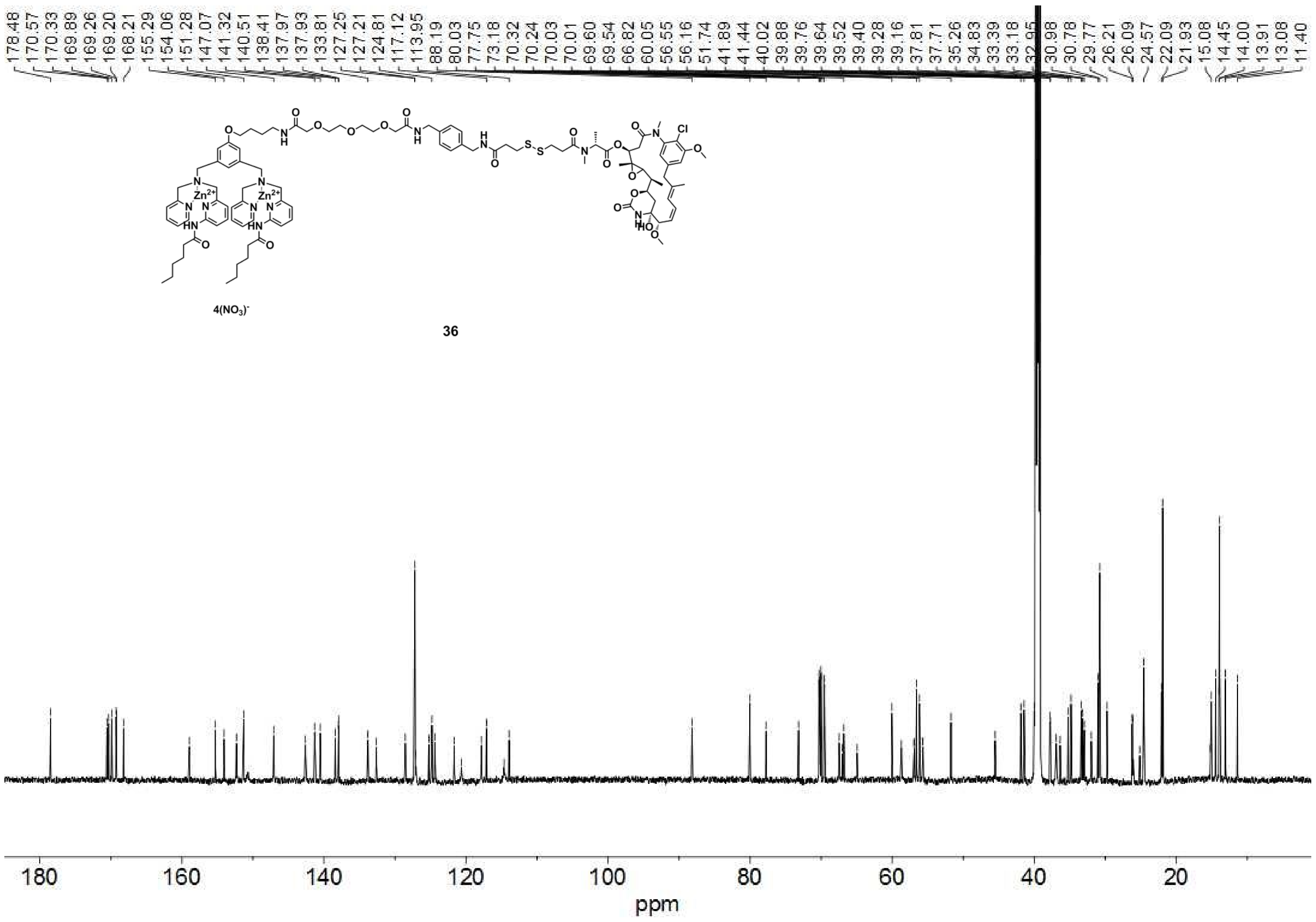
178.48
170.57
170.33
169.89
169.26
169.20
168.21
155.29
154.06
151.28
147.07
141.32
140.51
138.41
137.97
137.93
133.81
127.25
127.21
124.81
117.12
113.95
88.19
80.03
77.75
73.18
70.32
70.24
70.03
70.01
69.60
69.54
66.82
60.05
56.55
56.16
51.74
41.89
41.44
40.02
39.88
39.76
39.64
39.52
39.40
39.28
39.16
37.81
37.71
35.26
34.83
33.39
33.18
32.95
30.98
30.78
29.77
26.21
26.09
24.57
22.09
21.93
15.08
14.45
14.00
13.91
13.08
11.40
4(NO3)-
36
180
160
140
120
100
80
60
40
20
ppm

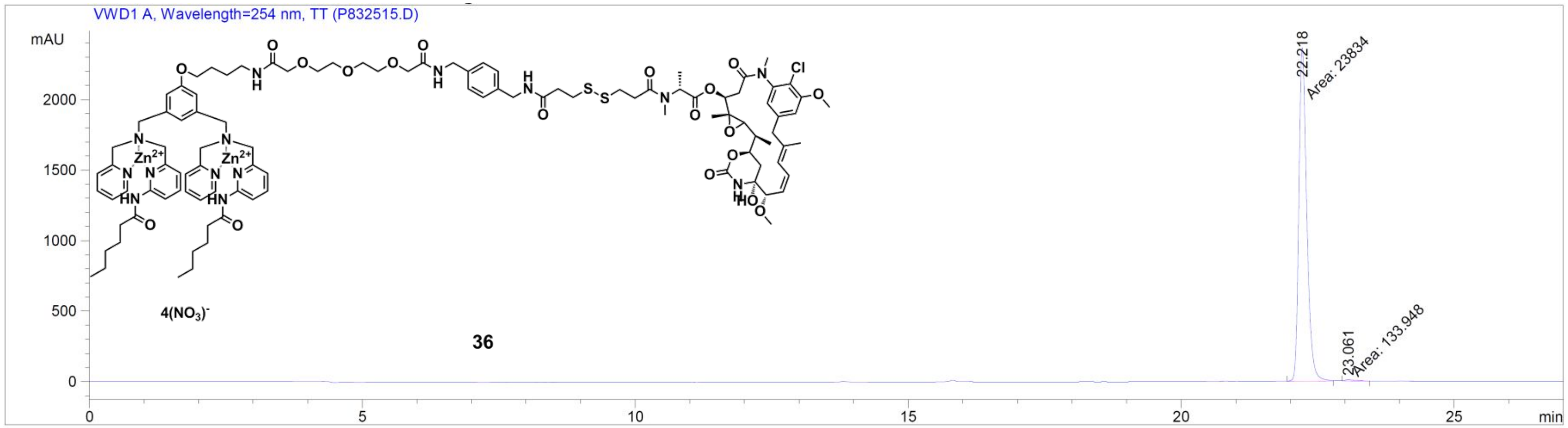


```
==================================================================
                        Area Percent Report
==================================================================

Sorted By             :      Signal
Multiplier            :      1.0000
Dilution              :      1.0000
Use Multiplier & Dilution Factor with ISTDs


Signal 1: VWD1 A, Wavelength=254 nm, TT

Peak RetTime Type  Width     Area      Height     Area
  #   [min]        [min]  mAU   *s   [mAU   ]       %
----|-------|----|-------|----------|----------|--------|
   1  22.218 MM    0.1677 2.38340e4  2368.13452  99.4411
   2  23.061 MM    0.2624  133.94795    8.50651   0.5589

Totals :                  2.39679e4  2376.64103
```

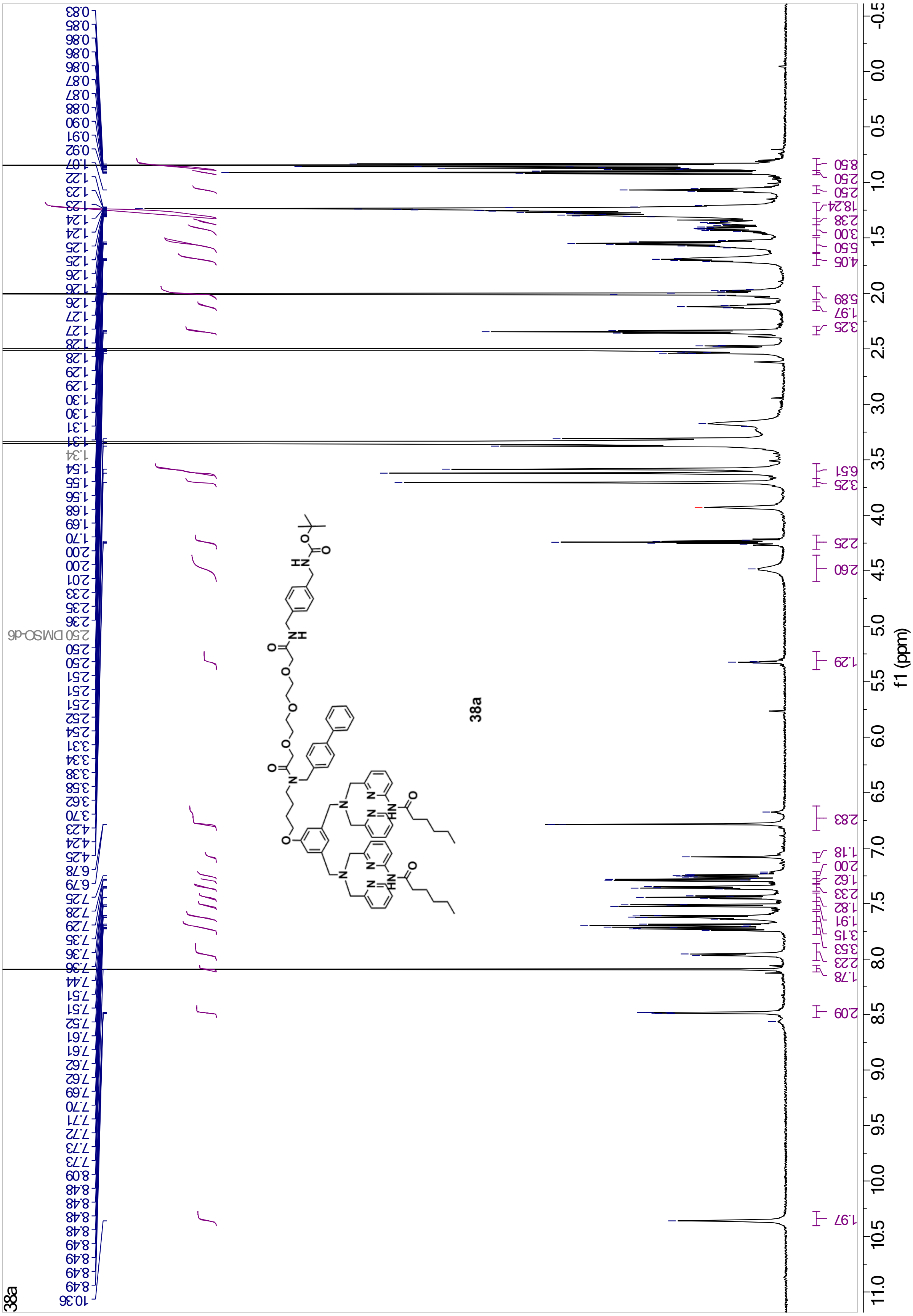
38a
2.50 DMSO-d6
f1 (ppm)
38a

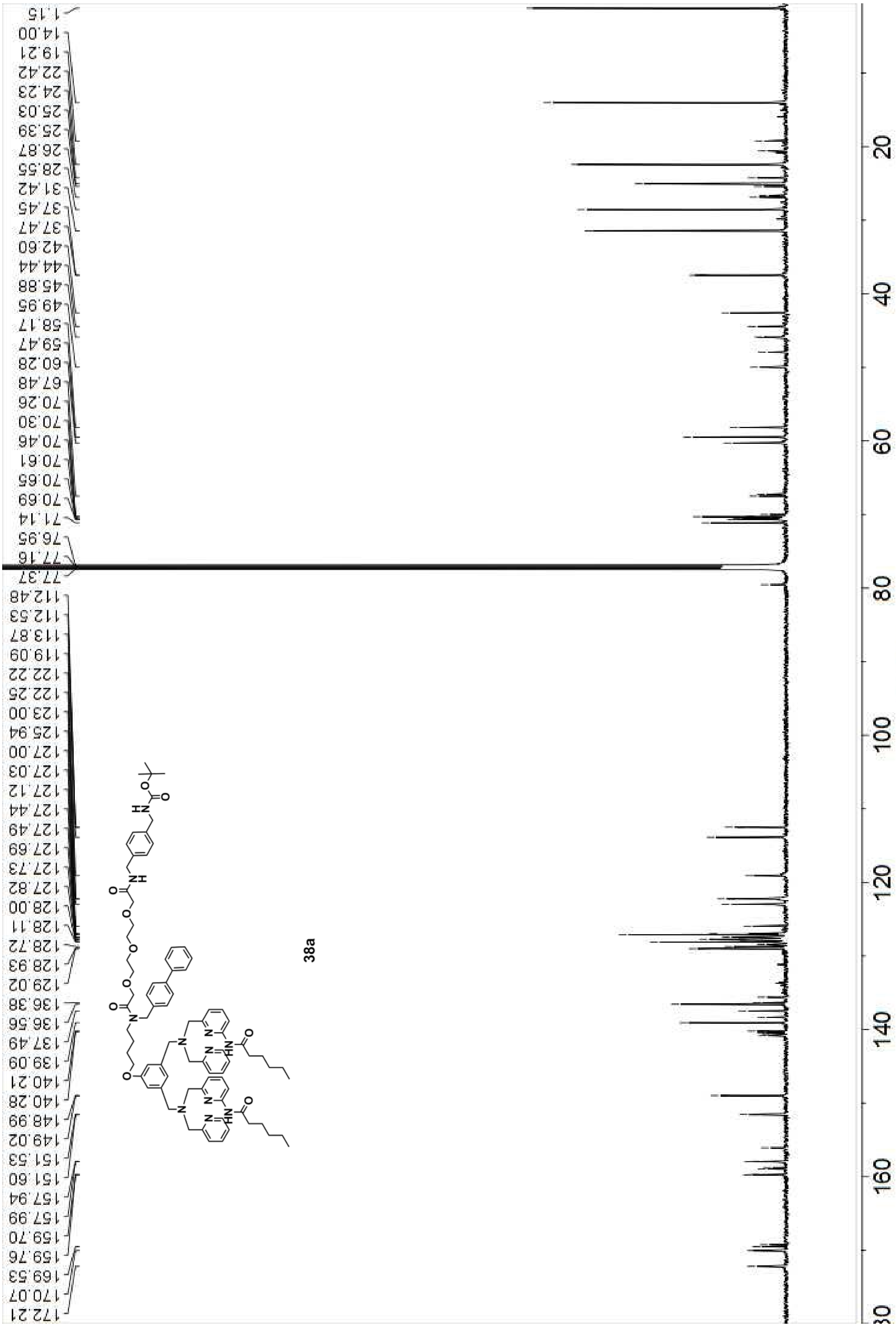
1.15
14.00
19.21
22.42
24.23
25.03
25.39
26.87
28.55
31.42
37.45
37.47
42.60
44.44
45.88
49.95
58.17
59.47
60.28
67.48
70.26
70.30
70.46
70.61
70.65
70.69
71.14
76.95
77.16
77.37
112.48
112.53
113.87
119.09
122.22
122.25
123.00
125.94
127.00
127.03
127.12
127.44
127.49
127.69
127.73
127.82
128.00
128.11
128.72
128.93
129.02
136.38
136.56
137.49
139.09
140.21
140.28
148.99
149.02
151.53
151.60
157.94
157.99
159.70
159.76
169.53
170.07
172.21
38a
20
40
60
80
100
120
140
160
ppm

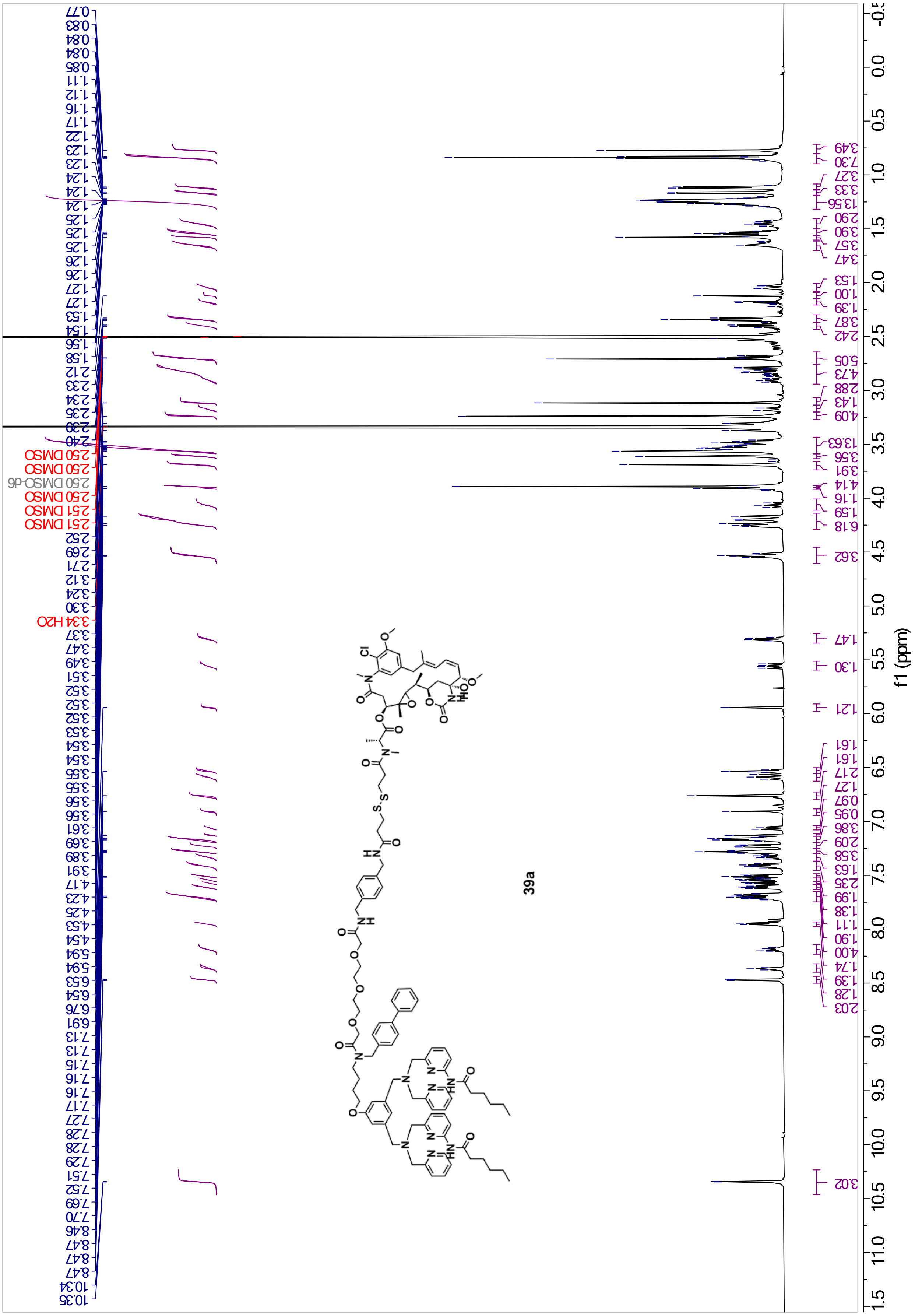
39a
f1 (ppm)

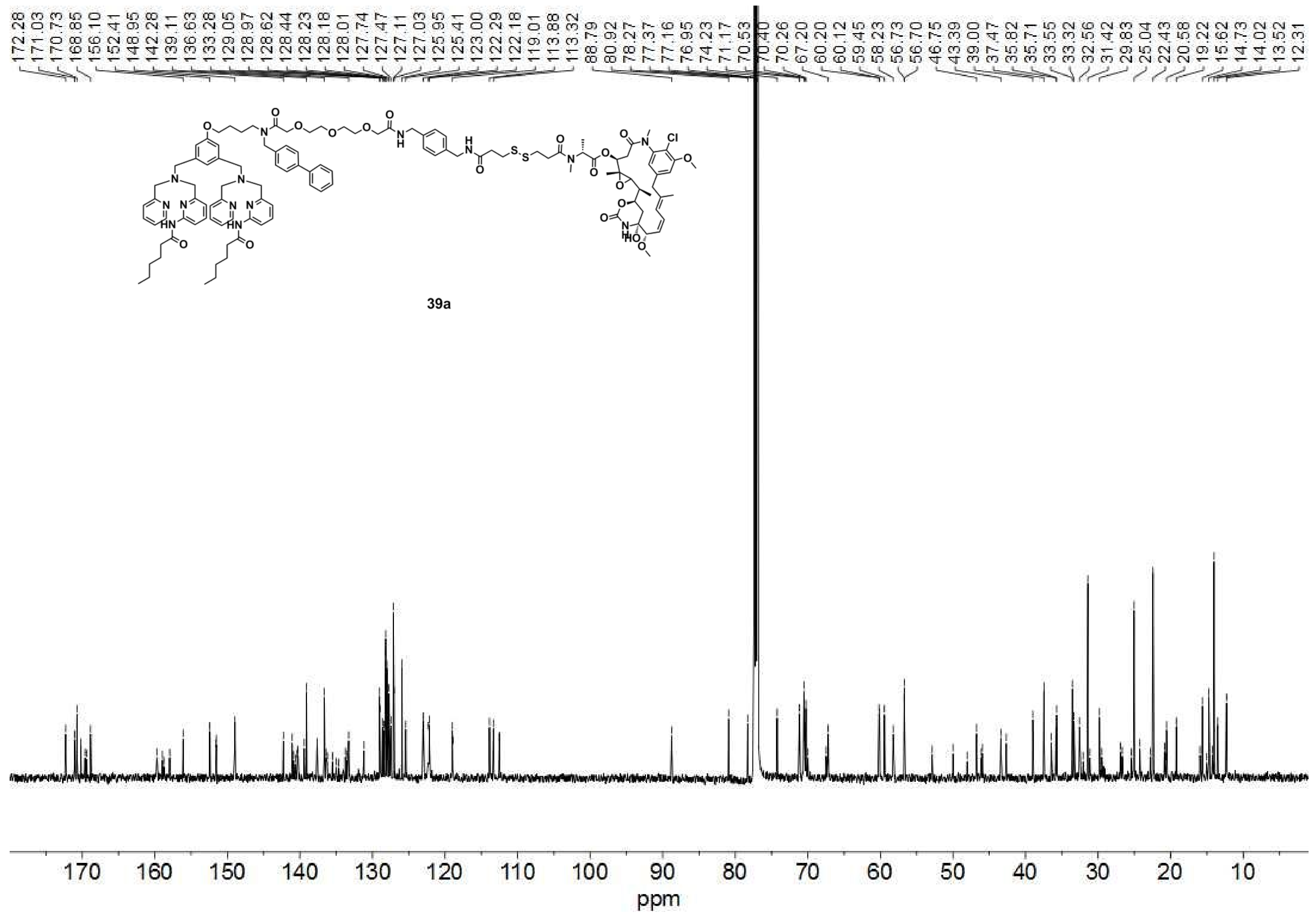
172.28
171.03
170.73
168.85
156.10
152.41
148.95
142.28
139.11
136.63
133.28
129.05
128.97
128.62
128.44
128.23
128.18
128.01
127.74
127.47
127.11
127.03
125.95
125.41
123.00
122.29
122.18
119.01
113.88
113.32
88.79
80.92
78.27
77.37
77.16
76.95
74.23
71.17
70.53
70.40
70.26
67.20
60.20
60.12
59.45
58.23
56.73
56.70
46.75
43.39
39.00
37.47
35.82
35.71
33.55
33.32
32.56
31.42
29.83
25.04
22.43
20.58
19.22
15.62
14.73
14.02
13.52
12.31
O
N
H
HN
S
Cl
HO
170
160
150
140
130
120
110
100
90
80
70
60
50
40
30
20
10
ppm

39a

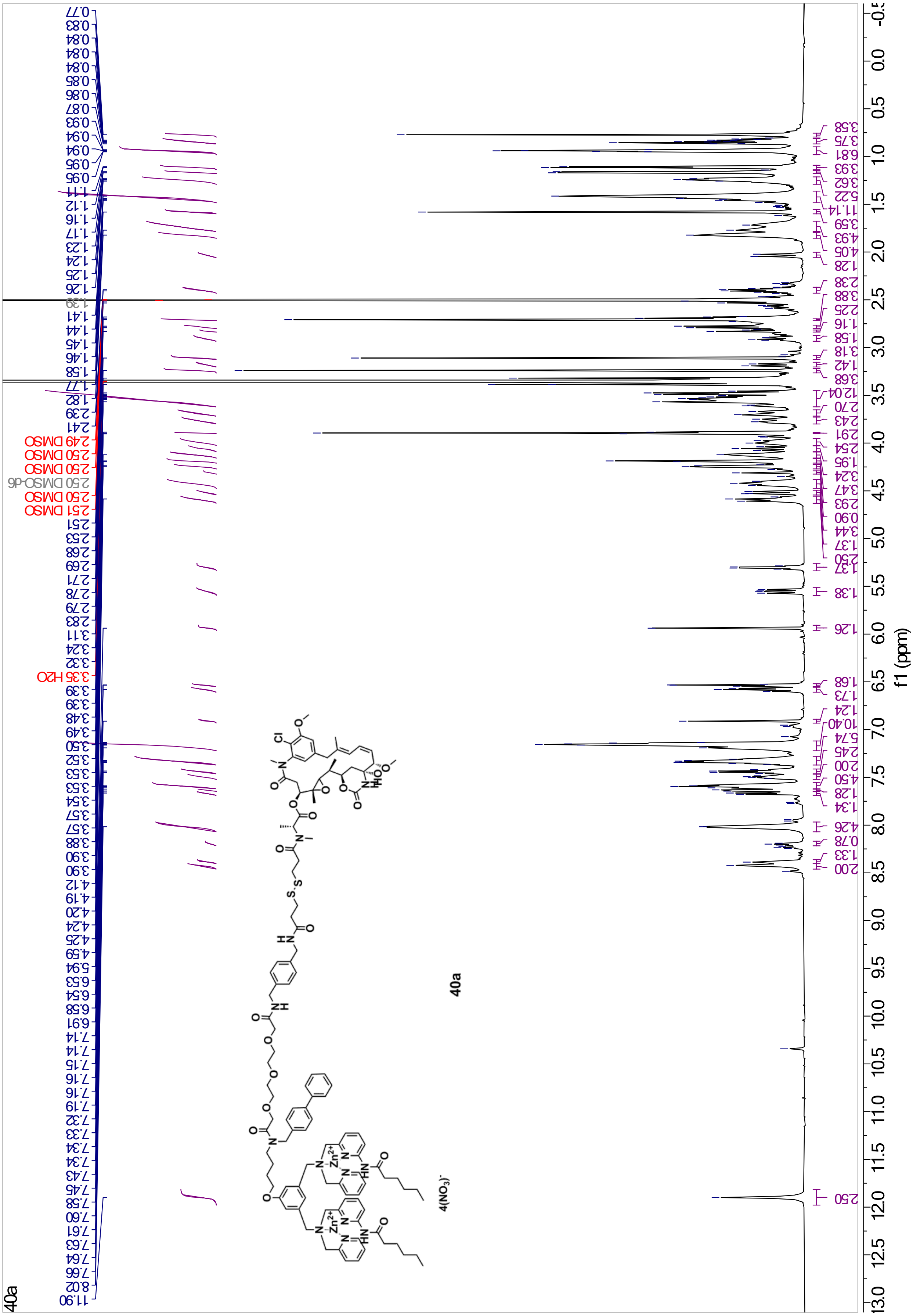
40a
f1 (ppm)

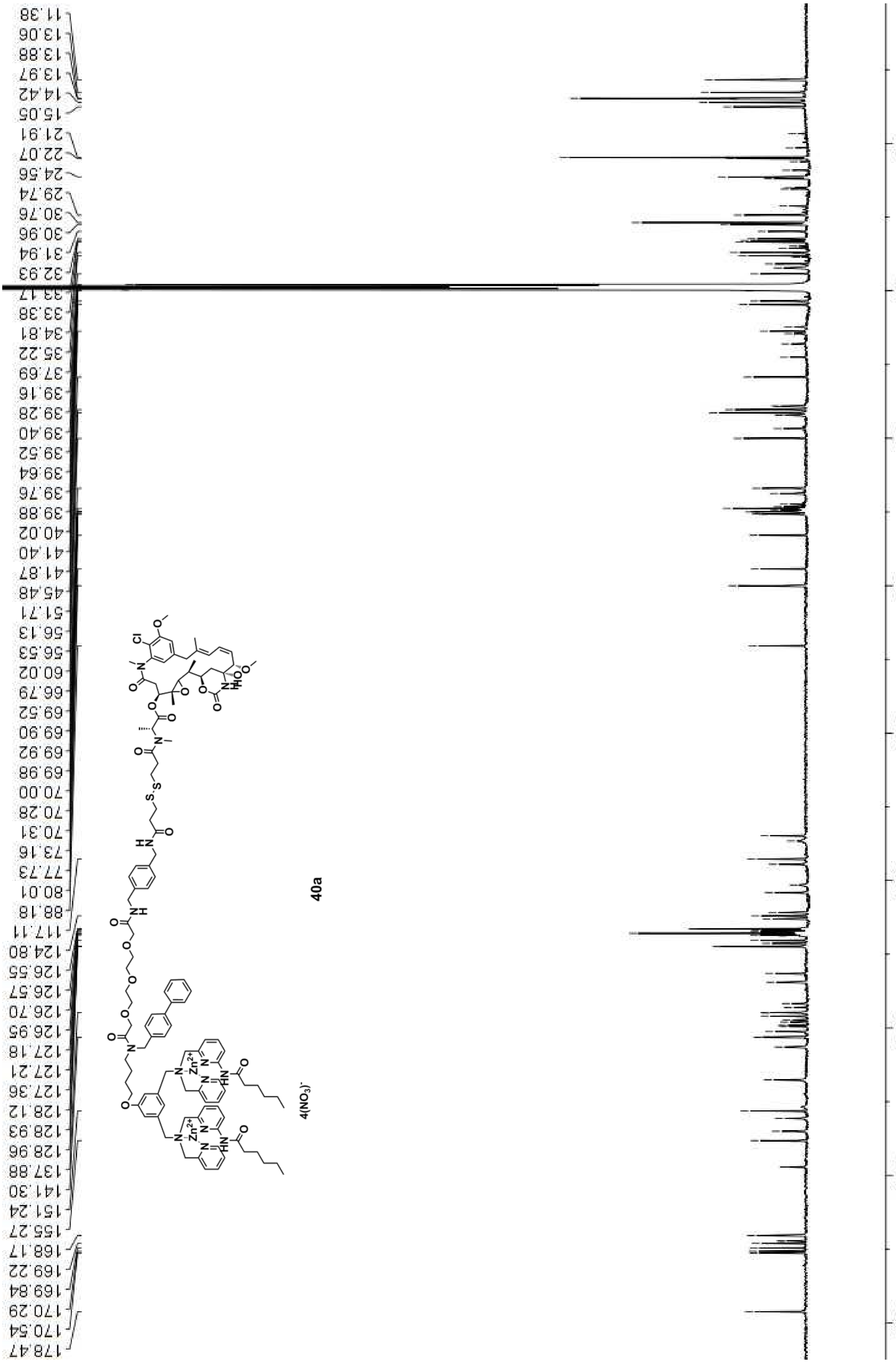
11.38
13.06
13.88
13.97
14.42
15.05
21.91
22.07
24.56
29.74
30.76
30.96
31.94
32.93
33.17
33.38
34.81
35.22
37.69
39.16
39.28
39.40
39.52
39.64
39.76
39.88
40.02
41.40
41.87
45.48
51.71
56.13
56.53
60.02
66.79
69.52
69.90
69.92
69.98
70.00
70.28
70.31
73.16
77.73
80.01
88.18
117.11
124.80
126.55
126.57
126.70
126.95
127.18
127.21
127.36
128.12
128.93
128.96
137.88
141.30
151.24
155.27
168.17
169.22
169.84
170.29
170.54
178.47
40a
4(NO3)-
ppm
20
40
60
80
100
120
140
160
180

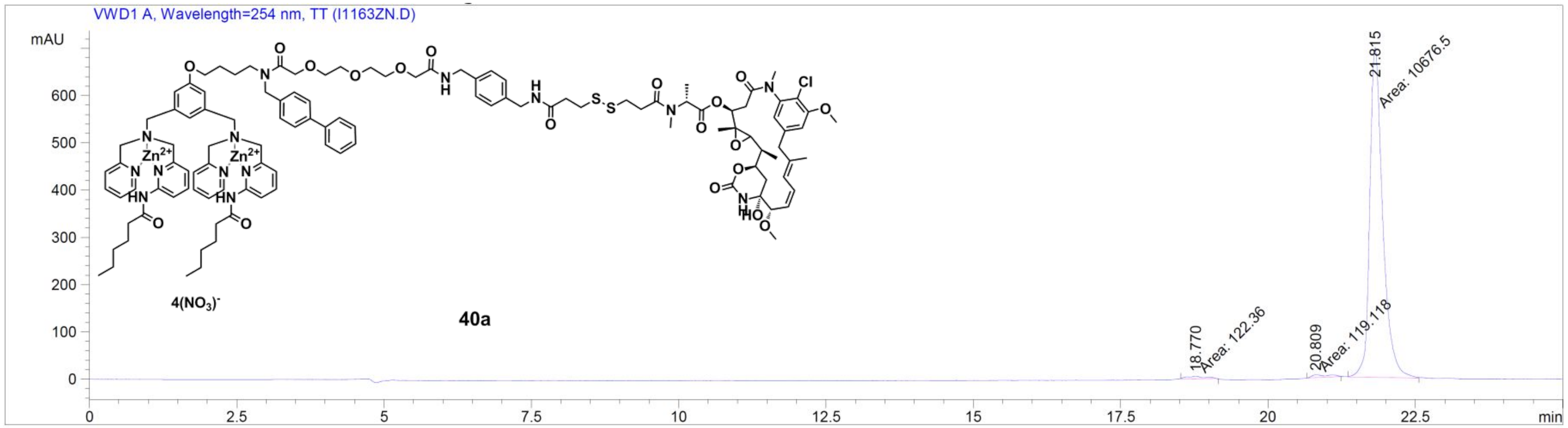


```
=====================================================================
                         Area Percent Report
=====================================================================

Sorted By             :      Signal
Multiplier            :      1.0000
Dilution              :      1.0000
Use Multiplier & Dilution Factor with ISTDs


Signal 1: VWD1 A, Wavelength=254 nm, TT

Peak RetTime Type  Width     Area      Height     Area
  #   [min]        [min]  mAU    *s   [mAU    ]     %
----|-------|----|-------|----------|----------|--------|
   1  18.770 MM    0.3429  122.35990    5.94789   1.1207
   2  20.809 MM    0.3258  119.11810    6.09356   1.0910
   3  21.815 MM    0.2549 1.06765e4   698.05780  97.7883
```

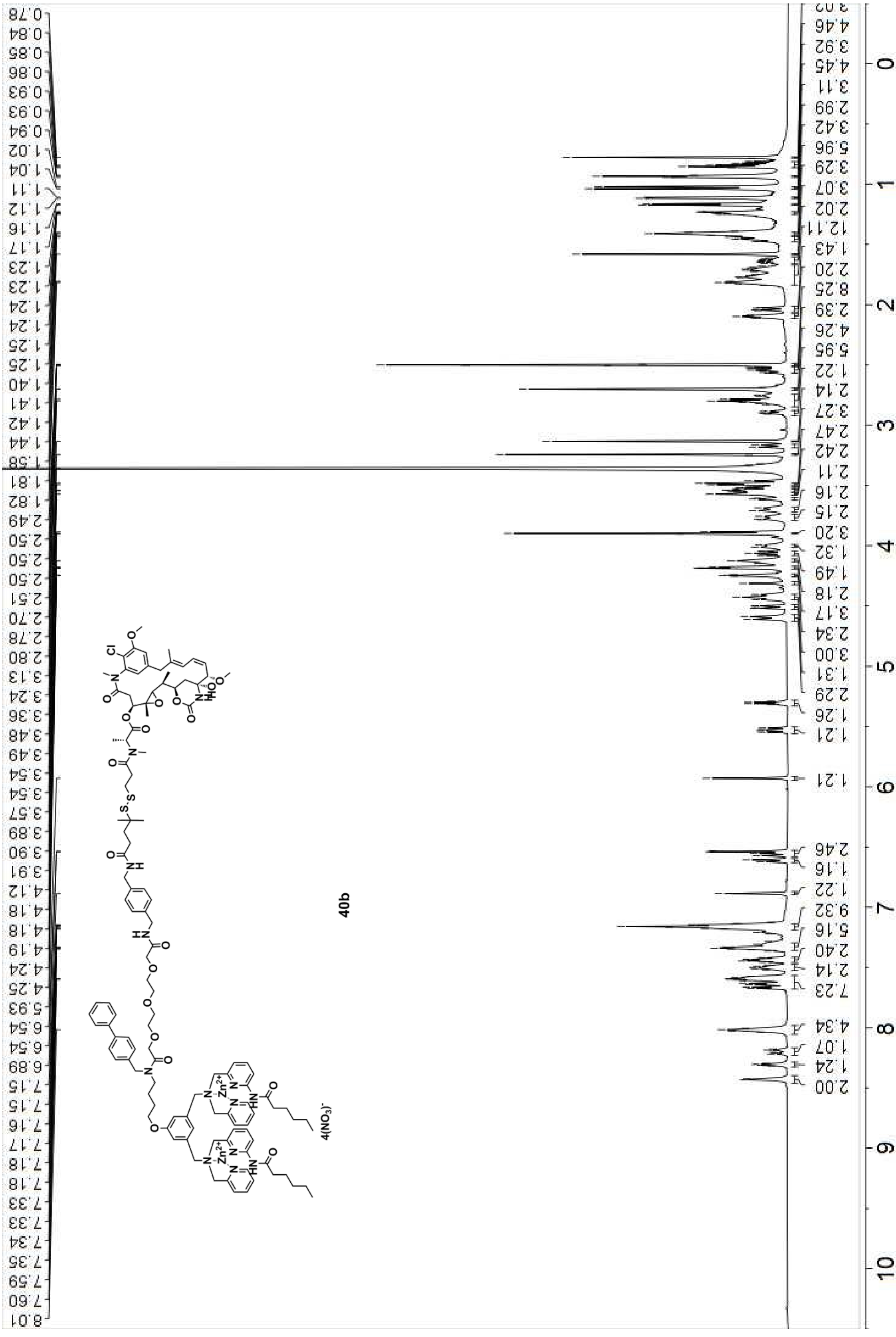
40b
ppm

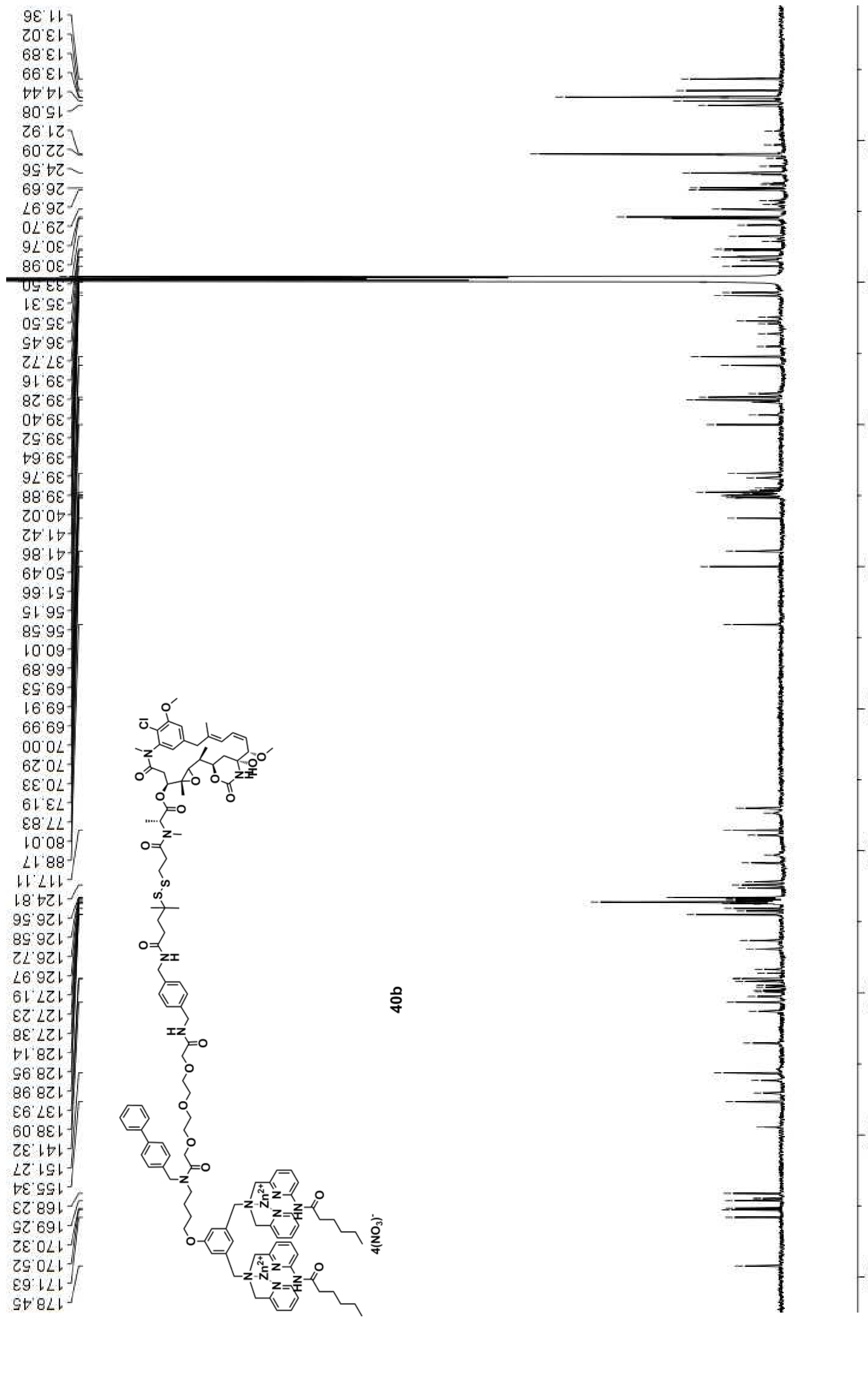
40b
ppm

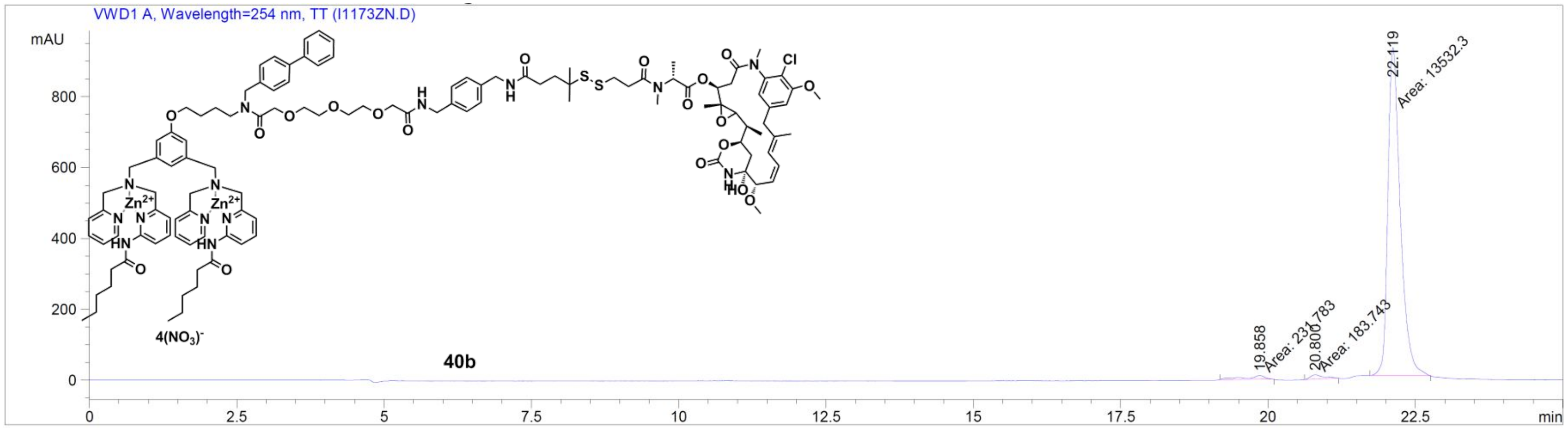


```
=====================================================================
                     Area Percent Report
=====================================================================

Sorted By             :      Signal
Multiplier            :      1.0000
Dilution              :      1.0000
Use Multiplier & Dilution Factor with ISTDs


Signal 1: VWD1 A, Wavelength=254 nm, TT

Peak RetTime Type  Width     Area      Height     Area
  #   [min]        [min]  mAU    *s   [mAU   ]      %
----|-------|----|-------|----------|----------|--------|
   1  19.858 MM    0.3713  231.78299   10.40514   1.6618
   2  20.800 MM    0.2534  183.74336   12.08529   1.3174
   3  22.119 MM    0.2437 1.35323e4   925.30743  97.0209
```

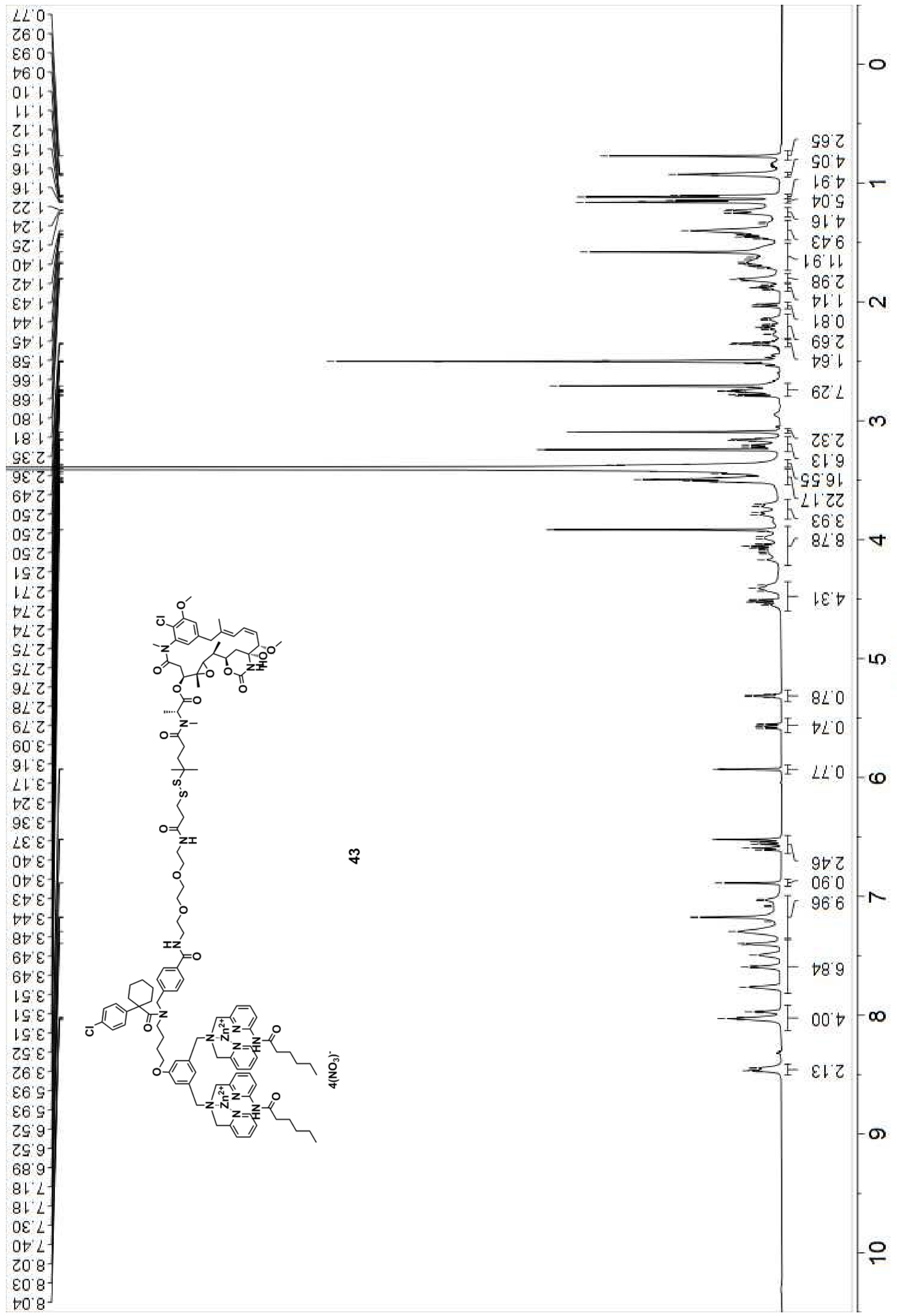

43

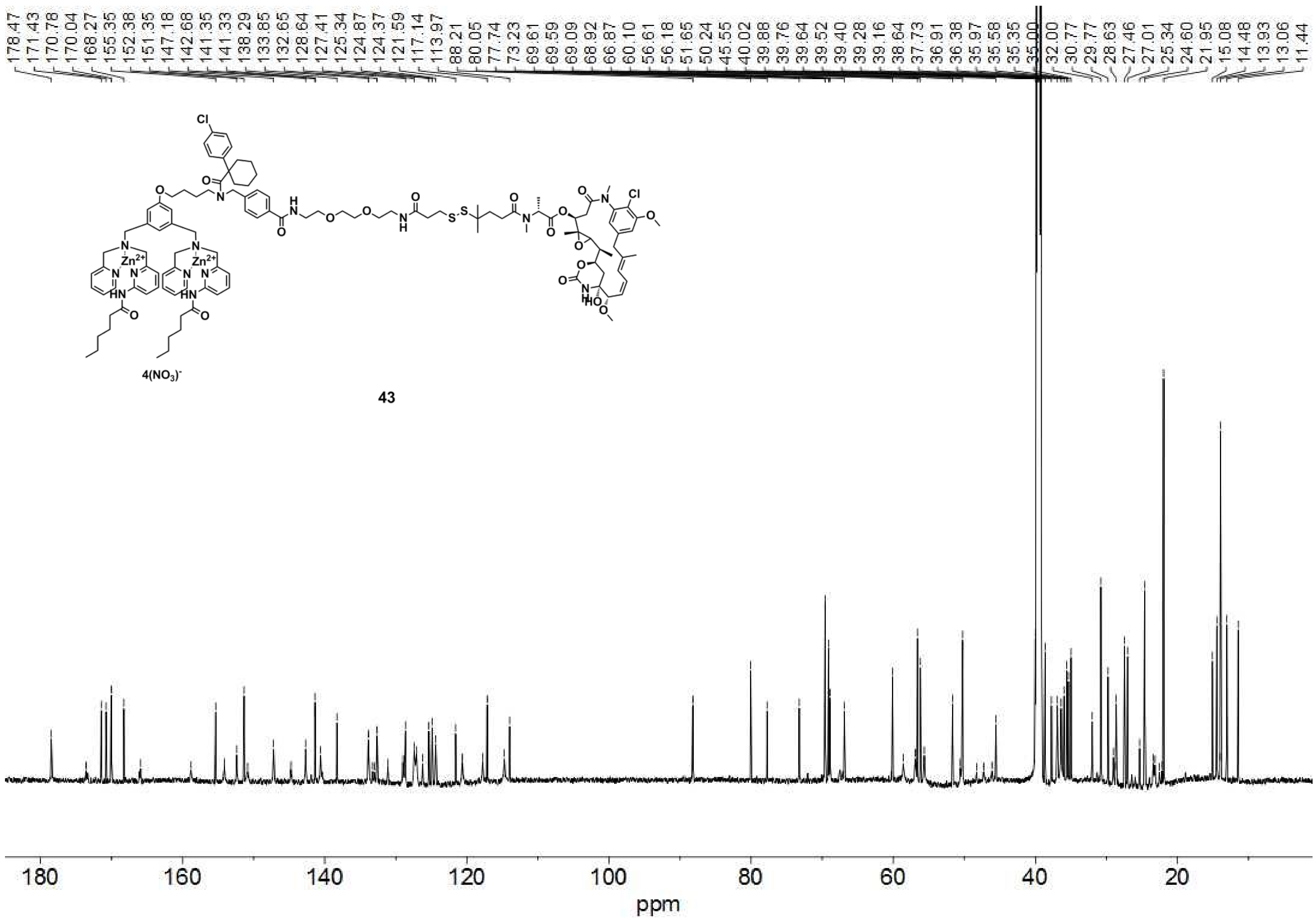
178.47
171.43
170.78
170.04
168.27
155.35
152.38
151.35
147.18
142.68
141.35
141.33
138.29
133.85
132.65
128.64
127.41
125.34
124.87
124.37
121.59
117.14
113.97
88.21
80.05
77.74
73.23
69.61
69.59
69.09
68.92
66.87
60.10
56.61
56.18
51.65
50.24
45.55
40.02
39.88
39.76
39.64
39.52
39.40
39.28
39.16
38.64
37.73
36.91
36.38
35.97
35.58
35.35
35.00
32.00
30.77
29.77
28.63
27.46
27.01
25.34
24.60
21.95
15.08
14.48
13.93
13.06
11.44
Cl
Zn2+
Zn2+
4(NO3)-
180
160
140
120
100
80
60
40
20
ppm

43

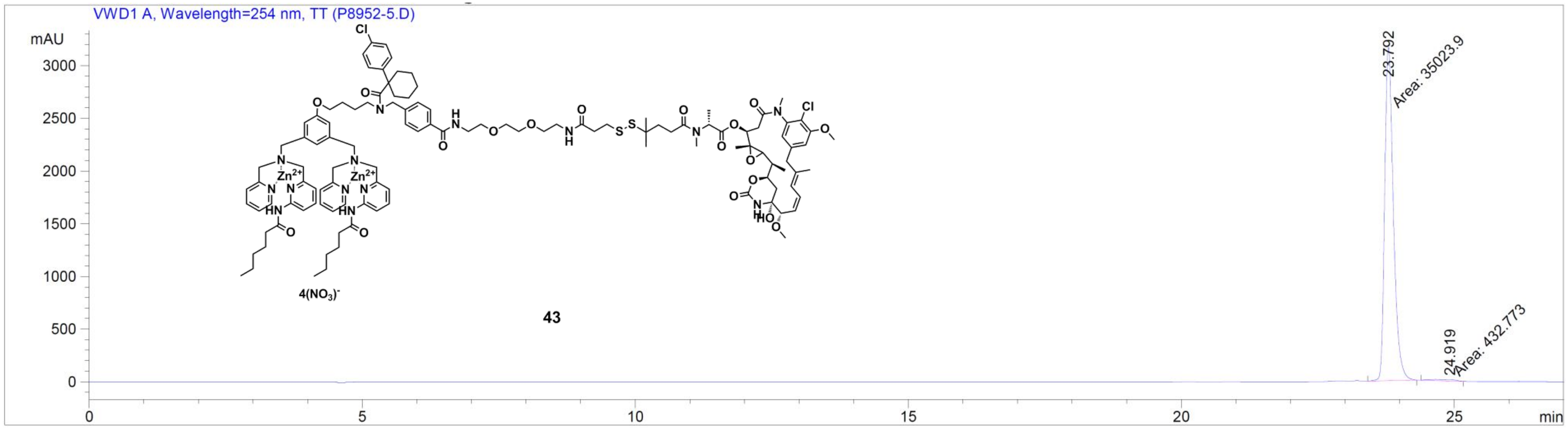


```
=====================================================================
                          Area Percent Report
=====================================================================

Sorted By             :      Signal
Multiplier            :      1.0000
Dilution              :      1.0000
Use Multiplier & Dilution Factor with ISTDs


Signal 1: VWD1 A, Wavelength=254 nm, TT
```

Peak #	RetTime [min]	Type	Width [min]	Area mAU *s	Height [mAU]	Area %
1	23.792	MM	0.1845	3.50239e4	3163.94995	98.7794
2	24.919	MM	0.4992	432.77347	14.44762	1.2206

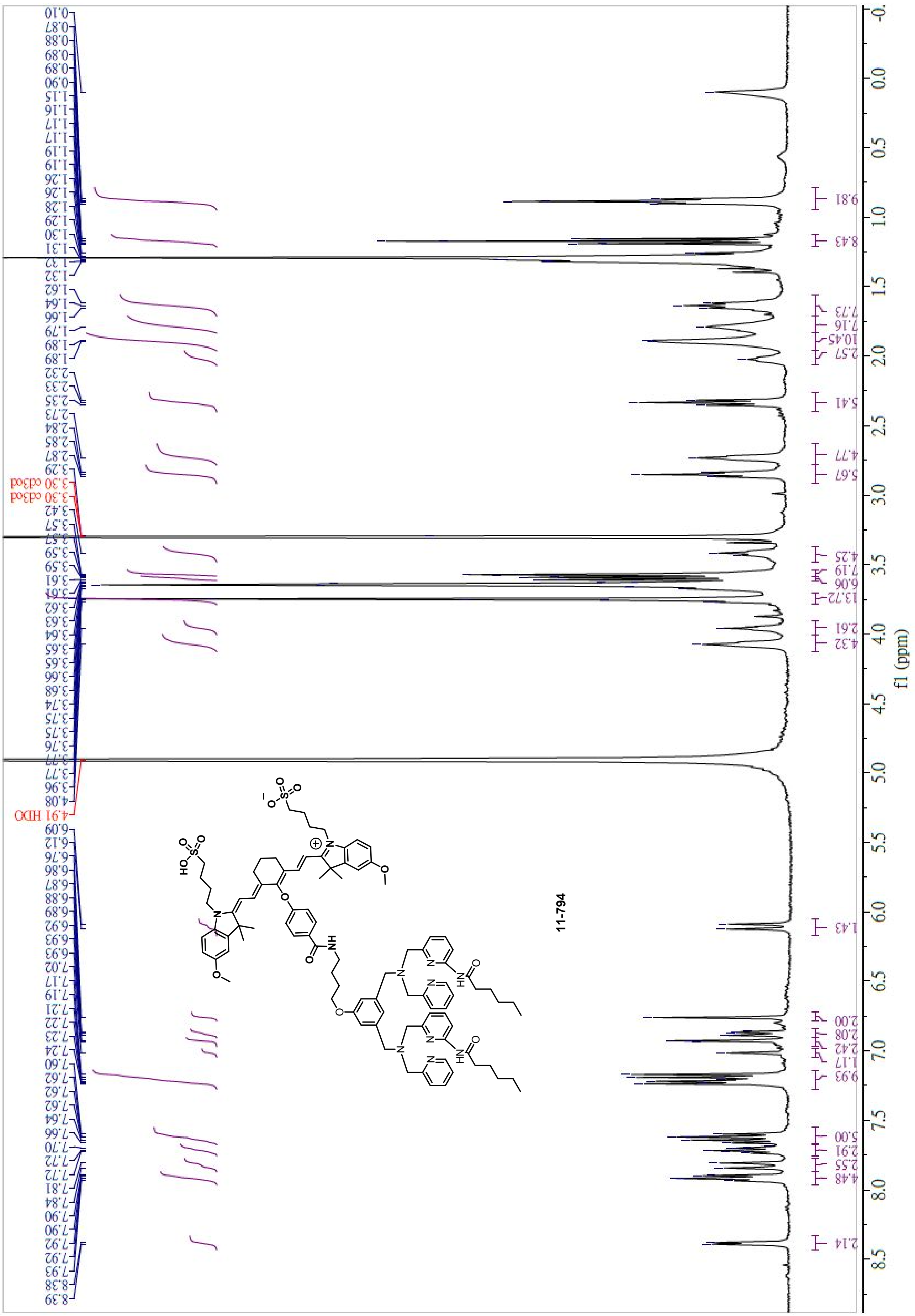
11-794
f1 (ppm)

Acquisition Parameter

Source Type	ESI	Ion Polarity	Positive	Set Nebulizer	1.0 Bar
Focus	Active	Set Capillary	4500 V	Set Dry Heater	200 °C
Scan Begin	50 m/z	Set End Plate Offset	-500 V	Set Dry Gas	6.0 l/min
Scan End	3000 m/z	Set Charging Voltage	2000 V	Set Divert Valve	Waste
		Set Corona	0 nA	Set APCI Heater	0 °C

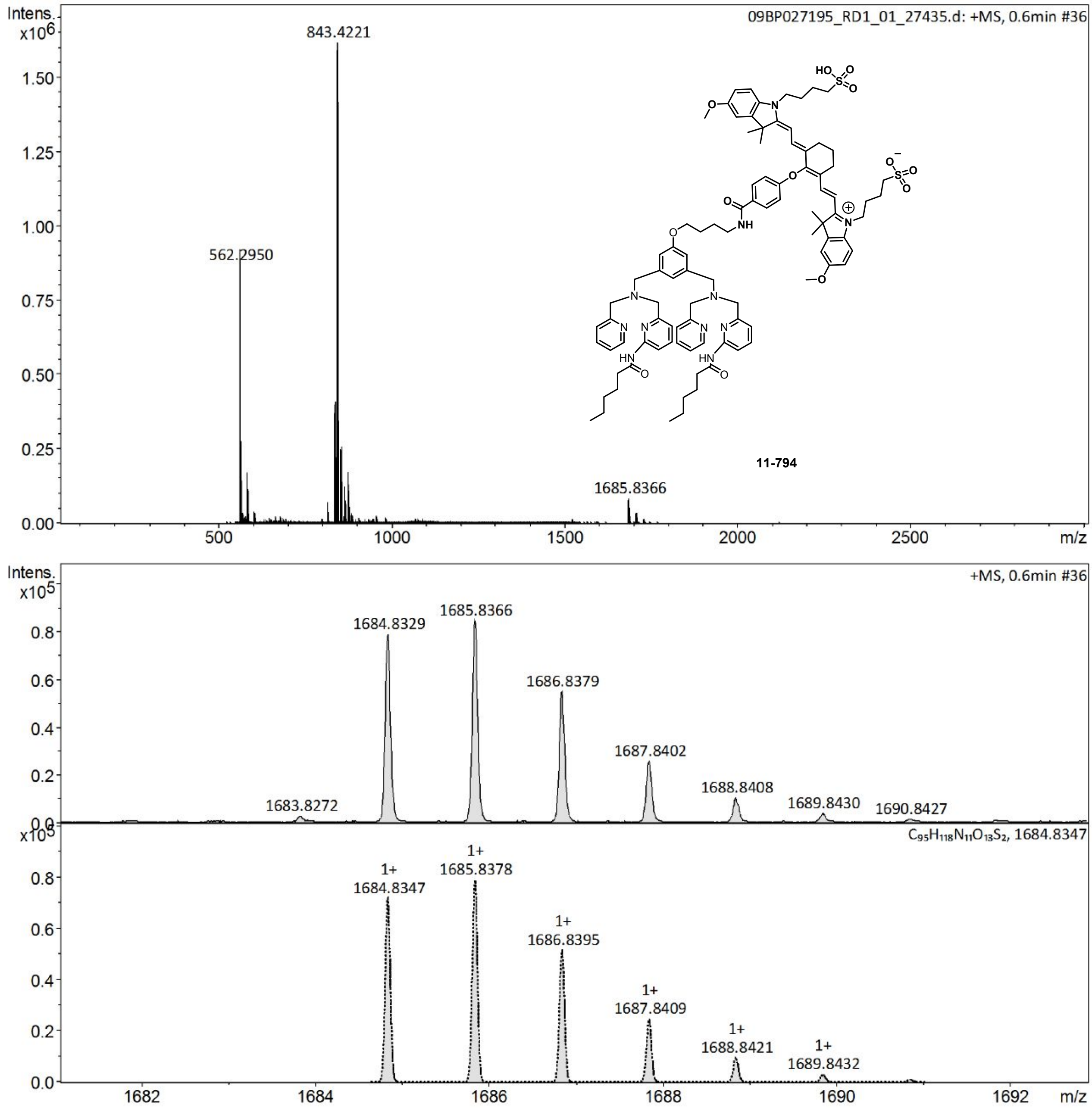

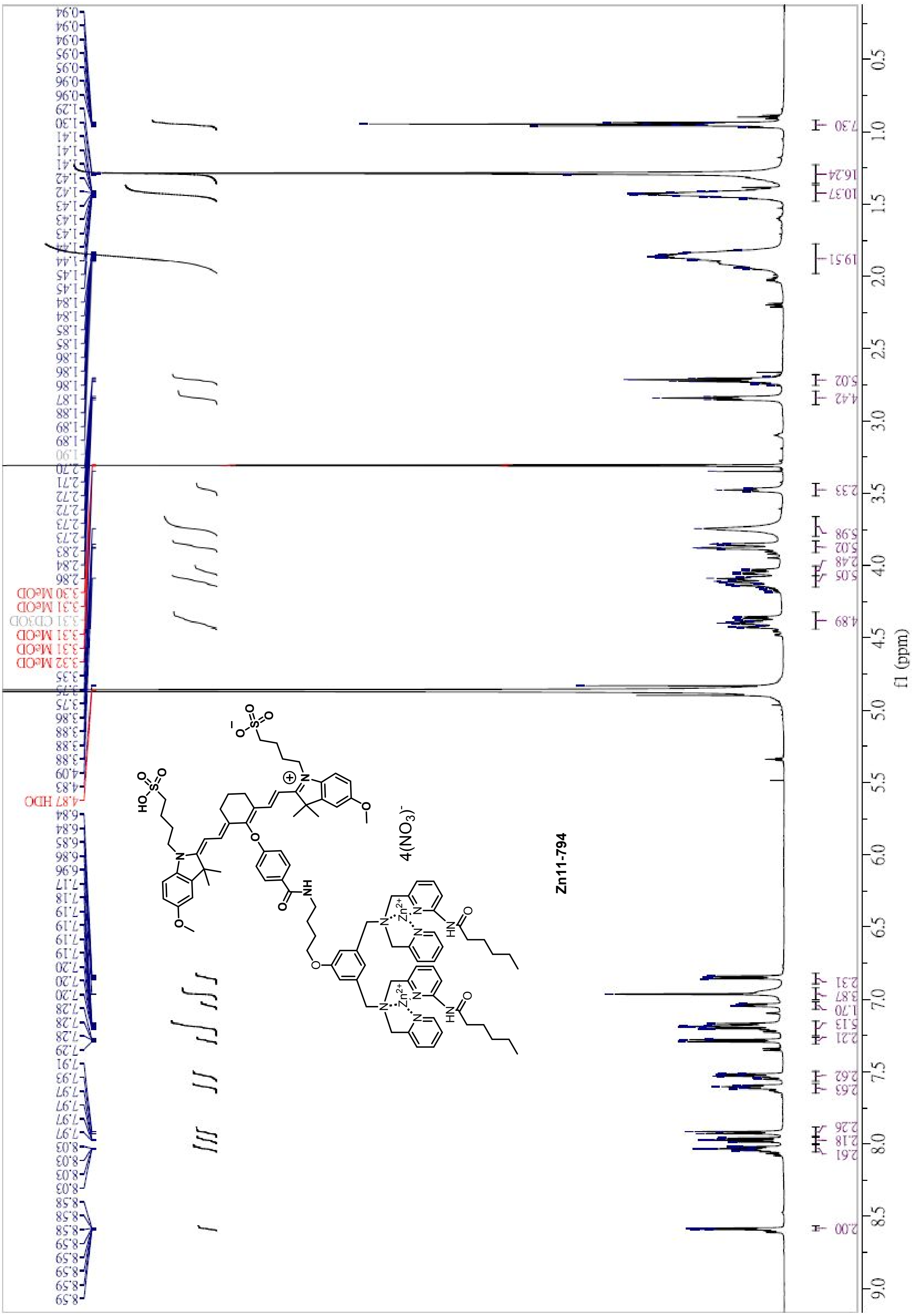
Zn11-794
4(NO3)-
f1 (ppm)

Acquisition Parameter

Source Type	ESI	Ion Polarity	Positive	Set Nebulizer	1.0 Bar
Focus	Active	Set Capillary	4500 V	Set Dry Heater	200 °C
Scan Begin	50 m/z	Set End Plate Offset	-500 V	Set Dry Gas	6.0 l/min
Scan End	3000 m/z	Set Charging Voltage	2000 V	Set Divert Valve	Waste
		Set Corona	0 nA	Set APCI Heater	0 °C

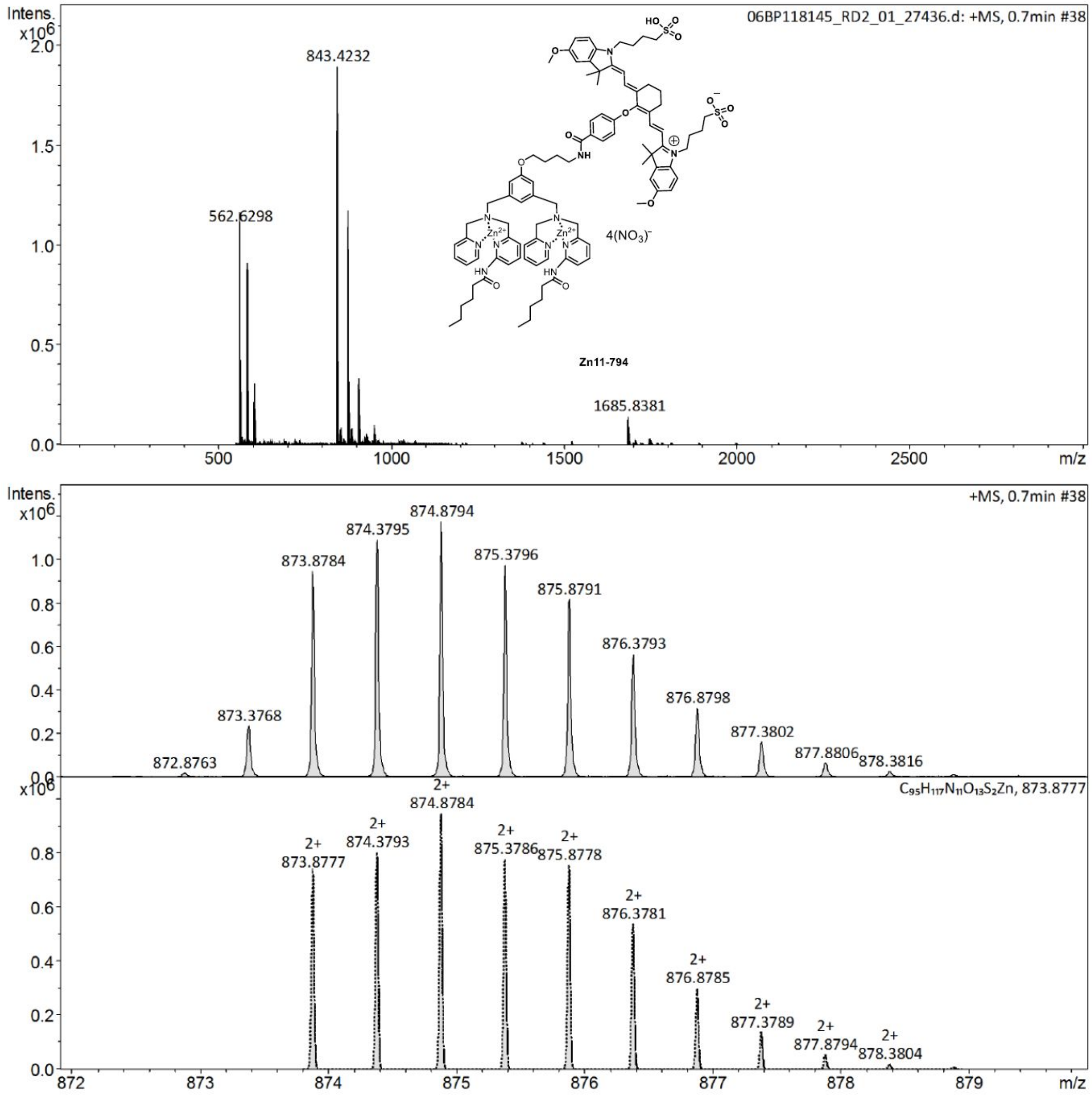

Meas. m/z	#	Ion Formula	m/z	err [ppm]	mSigma	# Sigma	Score	rdb	e⁻ Conf	N-Rule	Adduct
873.8784	1	C95H117N11O13S2Zn	873.8777	-0.8	51.2	1	100.00	43.0	even	ok	M

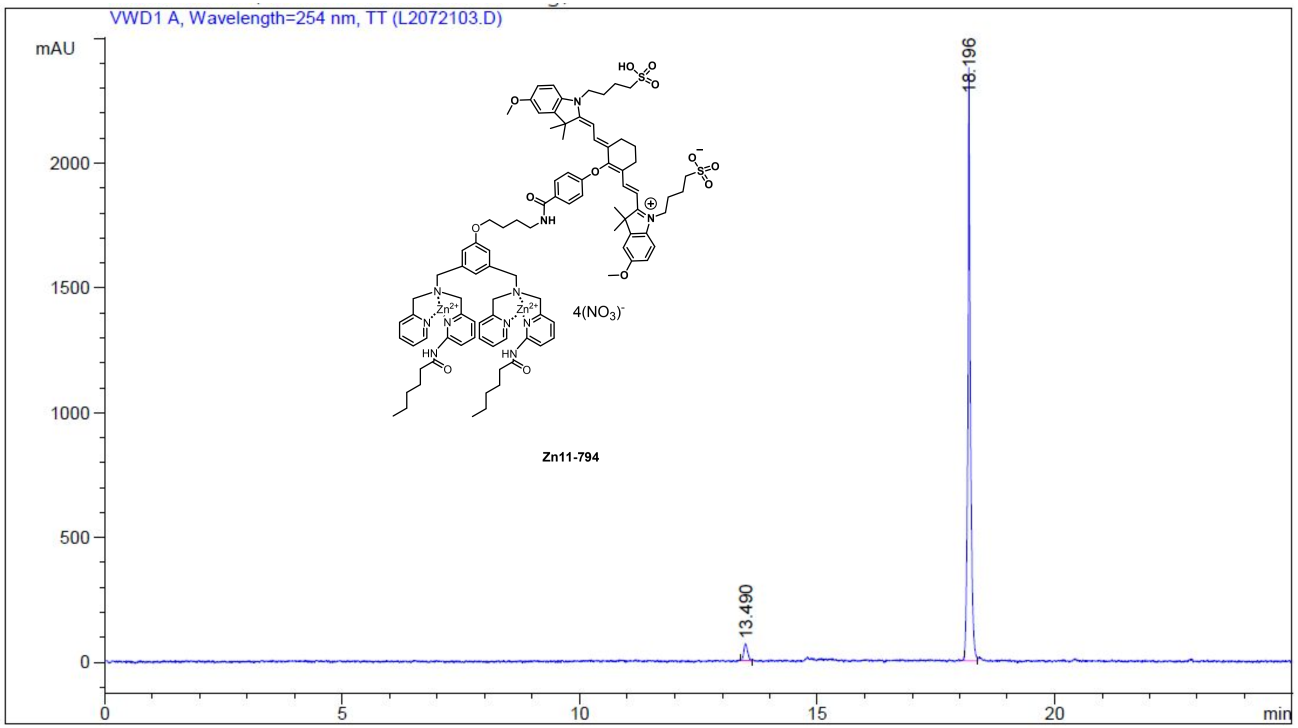


```
=====================================================================
                        Area Percent Report
=====================================================================

Sorted By             :      Signal
Multiplier            :      1.0000
Dilution              :      1.0000
Use Multiplier & Dilution Factor with ISTDs


Signal 1: VWD1 A, Wavelength=254 nm, TT
```

Peak #	RetTime [min]	Type	Width [min]	Area mAU *s	Height [mAU]	Area %
1	13.490	BB	0.0862	388.56964	68.84739	4.2353
2	18.196	BB	0.0533	8786.04004	2301.62500	95.7647
Totals :				9174.60968	2370.47239	
